# *In situ* identifying sennoside A-reducing bacteria guilds in human gut microbiota via enzymatic activity visualization

**DOI:** 10.1080/19490976.2025.2560598

**Published:** 2025-09-25

**Authors:** Chuanjia Zhai, Xinyue Liu, Zhen Liu, Huilin Ma, Huajinzi Li, Yingxi Gong, Xiang Li, Yingyue Wang, Na Zhang, Han Zhang, Gan Luo, Ying Wang, Xiaoyan Gao

**Affiliations:** School of Chinese Materia Medica, Beijing University of Chinese Medicine, Beijing, China

**Keywords:** Functional guilds, enzymatic activity visualization, substrate-based probes, FACS, sennoside A, nitroreductase

## Abstract

The intestinal environment determines the biochemical activity of individual microbial strains. The functions of microbial strains in their native environments often cannot be accurately predicted based solely on genomic information or the biochemical properties of cultivated isolates. In some cases, even the activity of intracellular enzymes does not correlate with the functional activity of bacteria. To address this challenge, we developed an enzymatic activity visualization platform that uses active intracellular enzymes as molecular baits to capture specific substrate probes. This platform links enzyme-mediated biochemical activity to their *in situ* localization, isolation, phylogenetic identification, and functional validation. This platform uses substrate-based probes designed to target isozymes, namely enzymes with different sequences but capable of catalyzing the same chemical reactions on a given substrate. Combined with a highly specific alkyne-azide cycloaddition reaction, these probes enable fluorescent visualization of strains harboring isozymes in human gut microbiota under anaerobic conditions. In addition, the platform can be integrated with fluorescence-activated cell sorting and 16S rRNA amplicon sequencing to isolate and taxonomically identify functional guilds containing isozymes physically. By performing metabolic capacity tests, fluorescence imaging, and proteomic analyses on four positive and three negative reference strains, we validated that our probes exhibit selectivity at the bacterial cell level. Using the platform, we successfully identified functional guilds involved in the reduction of sennoside A, a widely used laxative prodrug activated by gut bacteria. Importantly, we found that phylogenetically distinct bacteria perform similar metabolic activities toward sennoside A and discovered a novel sennoside A-reducing enzyme, StNfrA, from these functional guilds. The mechanistic study on StNfrA proved that our platform distinguished sennoside A-reducing bacteria species from microbiota by the enzymatic activity. Overall, these findings demonstrate that this enzymatic activity visualization platform is a powerful tool for the reliable localization, isolation, and identification of functional guilds in complex microbial communities.

## 1. Introduction

The human gut microbiome plays a critical role in human health, influencing both disease progression and therapeutic outcomes. Within this complex ecosystem, microorganisms do not act independently but instead form spatially organized, taxonomically diverse, and metabolically interdependent functional guilds that regulate microbial community functions.^1^ Studies have shown that these functional guilds are closely linked to various host phenotypes, such as obesity and type 2 diabetes.^2^ Furthermore, they are directly involved in the transformation of xenobiotics, altering their bioavailability, bioactivity, and toxicity.^3–5^ Identifying functional guilds associated with specific disease phenotypes or microbial drug metabolism is therefore essential for understanding and leveraging microbiota mechanisms in human health. Nonetheless, our knowledge of host−microbiota−xenobiotic interactions remains constrained by the absence of tools that can integrate the microbe−enzyme−level resolution with the ability to detect and quantify biochemical activity.

Advances in sequencing technologies and bioinformatics have established metagenomics as a key tool for investigating the identity and potential functions of microorganisms in complex microbiomes.^6^ Functional annotations of coding sequences are typically inferred by comparing them to reference databases. However, approximately 50% of genes in current genetic databases remain uncharacterized, and many annotations are incomplete or inaccurate.^7,8^ Moreover, the presence of a gene does not guarantee its functional activity, as gene abundance does not always correlate with functional expression.^9^ Microorganisms with nearly identical 16S ribosomal RNA (rRNA) gene sequences can exhibit significantly different *in situ* phenotypes.^10^ Thus, genomic data alone cannot reliably predict microbial functions. Traditional cultivation-based methods offer an alternative by directly isolating microorganisms performing specific functions.^11^ However, many gut microbial species require the gut environment to perform their functions and are difficult to cultivate in laboratory conditions.^12^ To date, a large proportion of gut microorganisms remain uncultured.^13^ This highlights the need for approaches that study the *in situ* functions of microorganisms to gain more accurate and detailed insights into microbial activity.

The human gut microbiome encodes a genetic repertoire 150 times larger than the human genome, representing a vast enzyme reservoir that expands the range of biotransformation reactions possible in the host.^14,15^ Enzymatic activity encoded by the microbiome plays a key role in microbial functions.^16^ We propose that measuring enzyme activity *in situ* has great potential for discovering functional microorganisms in complex communities. Activity-based probes (ABPs), derived from substrates, can interact with enzymes by mimicking their natural substrates. The binding of ABPs to proteins is based on enzyme – substrate interactions followed by covalent linkage. Therefore, ABPs exhibit good selectivity toward their target enzymes and the corresponding host cells. These probes, when combined with mass spectrometry, have been widely used to identify new enzymes in cultured microorganisms.^17–19^ By coupling ABPs with bioorthogonal fluorescence labeling, enzyme activity can be tracked in live cells under native conditions.^20,21^ This approach provides direct functional insights by linking fluorescence signals to microorganisms with specific enzymatic activities, offering an efficient pathway for discovering functional microorganisms.^22^

The ABPs enable nondestructive analysis of microbial functions at the single-cell level in microbiomes. This capability allows the physical separation of individual cells based on their enzymatic activity using fluorescence-activated cell sorting (FACS).^23^ The isolated cells can then undergo downstream analyses, such as genome sequencing, to identify their taxonomic and functional profiles.^24,25^ By combining ABPs with FACS, microorganisms with active enzymes can be sorted in a function-dependent manner and identified through sequencing. The Wright group developed an approach called “Probe Enabled Viable Cell Isolation” by which chemical probes that select for β-glucuronidase activities were applied to isolate functionally active cells from gut microbiota.^26,27^ This enzyme activity-dependent approach has significant potential to facilitate the discovery of functional microorganisms.

Sennoside A (SA), a well-known laxative prodrug, is activated by gut bacteria.^28^ Previous research has shown that sennoside A is degraded by bacterial nitroreductases (NTRs), and multiple strains are known to metabolize sennoside A.^29^ However, a comprehensive understanding of the functional guilds harboring this class of NTRs is still lacking. In this study, we employed ABPs to localize and identify drug-metabolizing guilds with isozymes *in situ*. The ABPs were constructed and the selectivity of probe – target interactions at the cellular level was validated. Then, by integrating this approach with FACS and 16S rRNA sequencing, we were able to physically isolate and taxonomically identify these guilds within the human gut microbiome. By integrating this approach with FACS and 16S rRNA sequencing, we were able to physically isolate and taxonomically identify these guilds within the human gut microbiome. We further demonstrated the sennoside A-reducing capabilities of the isolated microorganisms and identified a novel sennoside A-reducing enzyme, StNfrA, from these functional guilds. The enzymes identified starting from the model enzymes were shown, as expected, to metabolize sennoside A, strongly demonstrating the molecular-level contribution of NTRs to the intestinal metabolism of sennoside A.

Overall, the enzymatic activity visualization platform developed in this study provides a robust strategy for accurately identifying drug-metabolizing guilds within the human gut microbiome. This platform holds great promise for advancing our understanding of microbiota-driven drug metabolism and its implications for human health.

## 2. Methods

### 2.1. Bacterial culture

*Clostridium butyricum* ATCC 19398, *Lactobacillus brevis* CICC 6239, *Escherichia coli* MG1655, *Akkermansia muciniphila* DSM 22959, *Bifidobacterium pseudocatenulatum* ATCC 27919, *Bifidobacterium breve* ATCC 15700, *Clostridium perfringens* ATCC 13124, *Lactobacillus fermentium* CICC 22827, *Bacteroides plebeius* DSM 17135, *Ruminococcus gnavus* VPI C7–9, and *Bacteroides ovatus* ATCC 8483 were used in this study. These strains were bought from BeNa Culture collection center. *Streptococcus thermophilus* CICC 6222 and *Limosilactobacillus reuteri* CICC 6132 were obtained from China Center of Industrial Culture Collection. All bacteria were cultured anaerobically in an anerobic chamber at 37°C overnight in Gifu Anaerobic Medium (GAM). Cultures were grown to an OD_600_ of 1.3–1.5 prior to use.

### 2.2. Isolation of bacterial cells from human faeces

The deposited fecal samples were thawed at room temperature for 3–5 min. To separate microbial aggregates and individual cells from fibrous debris, cecal contents were diluted with Phosphate Buffer solution (PBS) in a 50 mL tube. Samples were then vortexed for 10 s. The samples were transferred to an anaerobic chamber and added 25 mL of GAM medium and incubate for 5 min. After the incubation, the samples were vortexed for 10 s and stood for 5 min to precipitate the fragments. The cells were harvested by centrifugation at 700 × *g* for 15 min at room temperature and supernatants were transferred to a new tube. The cells were harvested by centrifugation at 8000 × *g* for 15 min and supernatants were removed.

### 2.3. Investigation of sennoside A-reducing activity of several strains

The blank control group without bacteria and the bacteria-drug co-incubation group (*n* = 3) were prepared. The incubation mixture (100 μM sennoside A) was prepared using 900 μL of bacterial culture (initial OD₆₀₀ = 0.2) and 40 μL of sennoside A (2 mg/mL, dissolved in 0.5% NaHCO₃ buffer).

All incubation mixtures were cultured in an anaerobic chamber (37°C, N₂ > 97%) for 24 h. After the incubation was completed, 100 μL of each sample was acidified with 10 μL acetic acid and extracted with 100 μL ethyl acetate. Then, the sample was derivatized with 10 μL of 1% DMNA solution, vortexed, and incubated for 10 min. 100 μL of 200 ng/mL emodin was added as internal standard. The mixture was centrifuged at 12,000 rpm for 10 min, the supernatant was transferred to a new tube and recentrifuged at 13,000 rpm for 15 min. The final supernatant was analyzed by UHPLC-HRMS in negative ion mode. The *m/z* values of compounds are as follows: 861.1884 (sennoside A, [M-H]^−^), 283.0248 (rhein, [M-H]^−^), 445.0776 (rhein-8-*O-β*-D-glucoside, [M-H]^−^), 563.1671 (rheinanthrone-8-*O-β*-D-glucoside derivative, [M-H]^−^), 401.1143 (rheinanthrone derivative, [M-H]^−^), 269.0456 (emodin, [M-H]^−^). The quantification of each compound was achieved using the peak area of characteristic ions-based Xcalibur software. The peak area ratio between the target compound and the internal standard is used as the quantitative basis.

### 2.4. Molecular dynamics (MD) simulations

The structures of BpNfrA and StNfrA were predicted by AlphaFold.^30^ The molecular docking was performed by AutoDock Vina v1.2.5.^31–33^ After docking, the binding pose with the highest docking scores was measured for stability and flexibility using molecular dynamics simulations. Molecular dynamics simulations were done using the GROMACS 2020.6 software package, the aber99SB force field and the SPC water model, and the force field parameters of the ligands were obtained from GAFF. A suitable size of solvent box was constructed and the corresponding number of sodium ions were added to make the total charge of the system neutral; further, the periodic boundary condition (PBC) was used in three directions of the system and the steepest descent method was used for energy minimization (EM); then the system was equilibrated with the 200 ps by using the normal-variable-temperature (NVT) system; after the system was equilibrated, the molecular dynamics simulations were performed at 310.15 K under the isothermal and isobaric system (NPT) for 100 ns by using V-rescale method and Berendsen method to control temperature and pressure, respectively. The cofactors in the proteins were restricted to the spatial positions in the X, Y, and Z directions in the simulation system mentioned above. Finally, the root-mean-square deviation (RMSD), Solvent Accessible Surface Area (SASA), Radius of Gyration of the atomic positions of the system were analyzed. The binding energies and key residues of the proteins and drug molecules were calculated and analyzed in combination with the gmx_MMPBSA 1.6.4 program.

### 2.5. Bio-layer interferometry

All bio-layer interferometry (BLI) experiments were conducted using an Octet R8 instrument (Sartorius, Germany) under consistent conditions: a shaking speed of 1000 rpm and a plate temperature of 30°C. Phosphate buffer (100 mM Na_2_HPO_4_, 100 mM NaH_2_PO_4_, pH 8.0) was used as the kinetics buffer throughout. Super Streptavidin (SSA) optical fiber probes were first equilibrated in phosphate buffer for 60 seconds, then loaded with 200 μL of biotinylated BpNfrA or StNfrA solution (50 μg/mL) for 600 seconds, followed by another 60-second baseline step in phosphate buffer. For binding kinetics assays, six serially diluted concentrations of sennoside A dissolved in phosphate buffer were added to a black polypropylene 96-well microplate (Greiner Bio-one, Germany), with phosphate buffer used to fill the remaining wells. One row was designated as a buffer-only negative control. Each well contained a total volume of 200 μL. The assay cycle for each concentration and probe type (enzyme-loaded or blank) included 60 seconds of baseline incubation in phosphate buffer, 120 seconds of association with the compound solution, and 60 seconds of dissociation in phosphate buffer. Data analysis was performed using Octet Analysis Studio. To correct for nonspecific binding, signals from blank probes and buffer-only negative controls were subtracted using the “Double References” mode. A 1:1 binding model was assumed for kinetics analysis.

### 2.6. Synthesis of probes

The synthetic route of SAP1 and SAP2 is shown in Fig. S1. For SAP1, sennoside A (32.24 mg) and compound 6 (7.31 mg) were dissolved in DMSO-EtOAc mixed solvents (DMSO: EtOAc = 1:1). The reaction mixture was then irradiated under UV light (302 nm) for 3 h. The reaction mixture was subsequently purified by flash column chromatography on C18 to yield SAP1 (8.33 mg, 20.21%). The synthetic route of compound 6 is shown in Supplementary information. For SAP2, sennoside A (25.02 mg), *N,N*’-dicyclohexylcarbodiimide (DCC, 5.98 mg) and *N*-hydroxysuccinimide (NHS, 6.67 mg), were dissolved in dry DMF (1 mL) under N_2_ environment. The reaction mixture was stirred for 12 h at room temperature in darkness. Then 2-(3-(but-3-yn-1-yl)-3 H-diazirin-3-yl)ethan-1-amine (1.99 mg, 0.0145 mmol) and *N,N*’-diisopropylethylamine (15.15 μL, 0.087 mmol) were added to the system and allowed to react for 2 h. The mixture was purified by column chromatography on silica gel eluted with methanol:2-propanol:0.1% ammonium hydroxide to give SAP2 as red solid in 18% yield. The ^1^H NMR and ^13^C NMR spectra of the products were shown in Supplementary information.

### 2.7. Probe labeling of BpNfrA and in-gel fluorescence imaging

For the substrated competition of SAP1, the reaction mixture consisted of 45 μL of BpNfrA (0.5 mg/mL) and sennoside A stock (200 mM) to make the final concentration of sennoside A 0 mM or 5 mM. After 1.5 h of incubation at 37°C, SAP1 was added to 50 μM. Each group of samples was incubated at room temperature in the absence of light and gently shaken for 20 min.

For SAP2, certain aliqouts of SAP2 stock (100 mM) were added to 45 μL of BpNfrA (0.5 mg/mL) and the final concentrations of sennoside A were 0 μM, 10 μM, 20 μM, 50 μM, 100 μM, and 200 μM. The reaction was incubated at 37°C for 1 h. After incubation, irradiate with a handheld UV lamp (wavelength: 365 nm) on ice for 10 min (for irradiating time experiment: 0/5/10/20 min).

After the labeling was completed, pre-mixed solutions of Cyanine5 (Cy5) azide, Tris (2-carboxyethyl) phosphine hydrochloride (TCEP), Tris (benzyltriazolylmethyl) amine (TBTA), and CuSO_4_ were added for click chemistry reaction, so that the final concentrations of Cy5 azide, TCEP, TBTA, and CuSO_4_ were 100 μM, 1 mM, 100 μM, and 1 mM, respectively. The click chemistry reaction was incubated at 37°C in the dark for 1 h. After click chemistry reaction, each group of samples was transferred to a new 1.5 mL EP tube and subjected to SDS-PAGE. The separation gels were stained with Coomassie Blue to show the load of proteins in each lane, and imaged the fluorescence of Cy5.

### 2.8. Probe targeting and labeling of sennoside A-reducing enzyme in bacteria

The blank control group without bacteria, the bacteria-SAP2 co-incubation group and the 3×sennoside A competition group (*n* = 3) was prepared.

The bacteria-SAP2 co-incubation group (100 μM SAP2, final concentration) was prepared by mixing 450 μL of secondary subculture bacteria (initial OD₆₀₀ = 0.2) with 50 μL of SAP2 solution (1 mM in dimethyl sulfoxide, DMSO). The bacteria-SAP2–3×SA co-incubation group (100 μM SAP2 and 300 μM sennoside A) was prepared by mixing 450 μL of bacteria from the second subculture with an initial OD₆₀₀ of 0.2 with 25 μL of SAP2 solution (2 mM in DMSO) and 25 μL of sennoside A solution (6 mM in DMSO).

The incubation and sample processing were performed as described in Section 2.3. The *m/z* values of target compounds were as follows: 980.2731 (SAP2, [M-H]^−^), 565.1696 (rhein-8-*O-β*-D-glucoside-probe, [M-H]^−^), 861.1884 (sennoside A, [M-H]^−^)，269.0456 (emodin, [M-H]^−^). The quantification of each compound was achieved using the peak area of characteristic ions. The peak area ratio between the target compound and the internal standard is used as the quantitative basis.

### 2.9. Gel electrophoresis experiment for probe-labeled strains

#### 2.9.1. Sennoside A competition experiment

With the same SAP2 concentration, we added 25× and 50× molar excess of sennoside A to compete with SAP2. The groups for sample pre-treatment of bacterial strains were prepared according to the following procedure:

a Control group: 25 μL DMSO was mixed with 450 μL of bacterial suspension (resuspend with PBS).

b. SAP2 group:25 μL DMSO was mixed with 450 μL of bacterial suspension (resuspend with PBS).

c. 25×SAP2 sennoside A group: 25 μL sennoside A solution (50 mM) was mixed with 450 μL of bacterial suspension (resuspend with PBS).

d. 50×SAP2 sennoside A: 25 μL sennoside A solution (100 mM) was mixed with 450 μL of bacterial suspension (resuspend with PBS).

Each group was incubated anaerobically at 37°C in the dark for 1 h After incubation completed, 25 μL DMSO was added to the solution of the control group and 25 μL SAP2 (2 mM) was added to the solution of the other groups respectively. Then, each group was incubated anaerobically at 37°C in the dark for 1 h.

#### 2.9.2. Dicoumarol inhibition experiment

Dicoumarol has been confirmed as an inhibitor of nitroreductase. We hypothesize that metabolically active bacterial strains primarily rely on nitroreductase to metabolize sennoside A. To validate this, we designed a dicoumarol inhibition experiment. The groups for sample pre-treatment of bacterial strains were prepared according to the following procedure:

a. Control group: 25 μL DMSO was mixed with 450 μL of bacterial suspension (resuspend with PBS).

b. SAP2 group:25 μL DMSO was mixed with 450 μL of bacterial suspension (resuspend with PBS).

c. 2.5×SAP2 dicoumarol: 25 μL dicoumarol stock (5 mM) was mixed with 450 μL of bacterial suspension (resuspend with PBS).

d. 5×SAP2 dicoumarol: 25 μL dicoumarol stock (10 mM) was mixed with 450 μL of bacterial suspension (resuspend with PBS).

Each group was incubated anaerobically at 37°C in the dark for 1 h. After incubation completed, 25 μL DMSO was added to the solution of the control group and 25 μL SAP2 (2 mM) was added to the solution of the other groups respectively. Then each group was incubated anaerobically at 37°C in the dark for 1 h.

#### 2.9.3. Gel electrophoresis experiment

After incubation completed, the above samples were irradiated with a handheld UV lamp (wavelength: 365 nm) on ice for 10 min. Then, bacterial lysis solution was added to each group of bacteria. The samples were ultrasonically disrupted on ice and centrifuged to obtain the supernatant soluble proteins. The protein concentration was determined by bicinchoninic acid assay. Each group of proteins was adjusted to 100 μg. And the protein solution was diluted with PBS to a total volume of 97.8 μL. Then, 2.2 μL pre-mixed solutions of Cy5 azide, TCEP, TBTA, and CuSO_4_ were added for click chemistry reaction. The click chemistry reaction was performed at 37°C in the dark for 1 h. After click chemistry reaction was completed, each group of samples was transferred to a new 2 mL centrifuge tube and the remaining click chemical reagents were washed. These samples were used for SDS-PAGE analysis. The separation gels were stained with Coomassie Blue to show the load of proteins in each lane, and imaged the fluorescence of Cy5.

### 2.10. Proteomics experimental methods

The bacterial cells were treated with 100 µM SAP2 probe for 1 h and irradiated with UV light for 10 mins on ice for photo-crosslinking. The control group was treated with an equivalent volume of dimethyl sulfoxide (DMSO) for 1 h and irradiated with UV light for 10 mins on ice. After probe labeling reaction was completed, the cells were washed with 0.1% DMSO/PBS to remove excess probe and collected. The cells were lysed by sonication on ice in cell lysis buffer. The protein concentration was measured. The cell lysates were conjugated to a biotin enrichment tag via click chemistry (100 µM Biotin-PEG3-N_3_, 100 µM TBTA, 1 mM CuSO_4_, and 1 mM TCEP, incubated at 37°C in the dark for 1 h). After the reaction completed, proteins were precipitated by adding four volumes of cold acetone and centrifugated at 10,000 × g for 10 min at 4°C. The protein precipitation were washed twice with cold methanol and redissolved in 0.1% SDS/PBS. The protein solution was diluted to a final concentration containing 0.04% SDS. Streptavidin magnetic beads were pre-washed with 0.05% Tween-20/PBS. The protein sample was incubated with streptavidin magnetic beads for 1 h to enrich biotinylated proteins. The beads were washed repeatedly with 0.05% Tween-20/PBS to remove nonspecifically bound proteins. The beads were collected and proteins were eluted by heating at 95°C for 3 min in 0.1% SDS/PBS. The eluted proteins were digested by adding 200 µL of 2 mM TCEP in 50 mM ammonium bicarbonate and incubated at 67°C for 10 min to breakage disulfide bonds. After TCEP was removed, alkylation was performed with iodoacetamide in the dark for 30 min. Trypsin was added at a 1:50 (w/w) enzyme-to-protein ratio, incubated at 4°C for 1 h, followed by addition of 100 µL of 50 mM ammonium bicarbonate and digestion overnight at 37°C. Finally, the digested peptides were vacuum-dried and prepared for nano LC-LTQ-Orbitrap-MS/MS analysis.

### 2.11. Probe labeling of bacterial cells

After 24 h of culturing, the bacteria were harvested by centrifugation at 10,000 g for 10 min at room temperature. Cell pellets were washed with PBS and then harvested by centrifugation as above mentioned.

For SAP1, the bacteria were cultured with either SAP1 (50 μM) or DMSO (negative control) anaerobically at 37°C for 30 min. The cells were washed with PBS and were harvested by centrifugation at 18,000 × *g* for 10 min at room temperature. After labeling with probe, Cy5 was attached via copper catalyzed azide-alkyne cycloaddition (CuAAC).

For SAP2, the bacteria were cultured with either SAP2 (10, 50, 100 μM) or DMSO (negative control) anaerobically at 37°C for 1 h, followed by UV irradiation (365 nm) for 10 min on ice. Cell pellets were fixed with 4% paraformaldehyde in PBS for 15 min. After fixing, the cells were washed three times with 1% DMSO/PBS and were harvested by centrifugation at 18,000 × *g* for 10 min at room temperature. After labeling with probe, Cy5 was attached via copper catalyzed azide-alkyne cycloaddition (CuAAC).

### 2.12. Cu(I)-catalyzed azide-alkyne cycloaddition staining of bacterial cells

Briefly, Cyanine5 azide (5 or 2.5 μM Cy5 in DMSO), TBTA (0.5 mM TBTA in tBuOH/DMSO (1:4, V/V)), TCEP (1 mM TCEP in H_2_O) and CuSO_4_ (1 mM CuSO_4_ in ddH_2_O) solutions were mixed in advance. The mixed solution was added to each bacterial cells group. The reaction was incubated at room temperature in the dark shaking on a vortexer for 1 h. The cells were washed 6 times with 2% BSA/PBS to remove any nonspecific fluorescent signals before fluorescence imaging and flow cytometry analysis. The control incubations were also treated with CuAAC reaction as mentioned before.

### 2.13. Confocal fluorescence imaging of probe labeled cells

For SAP1, the cells were washed with PBS and were harvested by centrifugation at 18,000 × *g* for 10 min at room temperature. Resuspended cells were mounted onto glass slides with agarose media. For SAP2, the cells were stained with 1 μg/mL Hoechst 33,342 (Solarbio) for 15 min and then washed and resuspended with PBS. Resuspended cells were mounted onto glass slides with agarose media.

All bacteria were analyzed using a Leica SP8 laser confocal microscope (Leica, Buffalo Grove, IL), all the images were analyzed using Fiji Image J software. Samples were imaged by using two color filter cubes: UV (359 nm/461 nm) for Hoechst to localize cells and Cy5 (650 nm/670 nm) to detect the presence of Probe labeled cells.

### 2.14. FACS gate optimization

Fluorescence-activated cell sorting to identity gut functional guilds. A BD Aria SORP (BD Biosciences, San Jose, CA) was sterilized by subsequent runs of 70% ethanol and autoclaved distilled water. The instrument was kept under sterile conditions during analysis and sorting by use of sheath fluid prepared using sterile 1×PBS. Cells were resuspended in 2 mM Na_2_EDTA/PBS before sorting. The Cy5 signal was excited using a 650 nm red laser, and fluorescence was captured with a 660 nm/20 nm filter. To remove potential cell debris (debris exclusion) and false positive signals detected from clumped cells (doublet exclusion) in the cecal content sample, the gating was further optimized using combinations of forward scatter (FSC) and side scatter (SSC) parameters. We used the control incubations (bacteria with Cy5 dye but without SAP2) to determine a gating strategy for artifact removal, thereby excluding populations exhibiting off-target labeling or passive dye accumulation. We defined the area of 95% bacteria in control incubations as the Cy5^−^ fraction and others were Cy5^+^ fraction. The gating strategy applied to control group fluorescence signals aimed to set a discrimination threshold that excludes background fluorescence attributable to nonspecific Cy5 dye adsorption. After establishing this gating strategy, the Cy5^−^ fraction and Cy5^+^ fraction was sorted in two separate 5 mL round-bottom tubes (BD Biosciences, San Jose, CA) containing PBS for imaging or 16S rRNA gene sequencing.

### 2.15. Sorting of SAP2 labeled cells from gut bacteria

After 24 h of culturing, the gut bacteria were harvested by centrifugation at 10,000 × *g* for 10 min at room temperature. SAP2 labeling was performed as described previously. Cells were resuspended in 2 mM Na_2_EDTA/PBS before sorting. FACS was performed as described. We defined the area of 95% bacteria in control incubations as the P4 (Cy5^−^) fraction. Then defined Cy5 signal greater than 120 fluorescent intensity as P6 gate (potential strong-metabolizers) or 0–120 fluorescent intensity as P5 gate (potential weak-metabolizers). Then about 60,000 events in each gate (P4, P5, and P6) were captured and segregated into two separate 5 mL round-bottom tubes (BD Biosciences) containing 1% sterile BSA/PBS. After sorting, cells were transferred to 1.5 mL tubes and centrifuged at 12,000 × *g* for 10 min at 4°C. The supernatant was discarded, and the sorted cells were collected for sequencing.

### 2.16. 16S rRNA gene amplification by PCR

Total community genomic DNA extraction was performed using a E.Z.N.A™ Mag Bind Soil DNA Kit (Omega, M5635–02, USA), following the manufacturer’s instructions. We measured the concentration of the DNA using a Qubit 4.0 (Thermo, USA) to ensure that adequate amounts of high-quality genomic DNA had been extracted. Our target was the V3-V4 hypervariable region of the bacterial 16S rRNA gene. PCR was started immediately after the DNA was extracted. The 16S rRNA V3-V4 amplicon was amplified using 2×Hieff Robust PCR Master Mix (Yeasen, 10105ES03, China). Two universal bacterial 16S rRNA gene amplicon PCR primers (PAGE purified) were used: the amplicon PCR forward primer (CCTACGGGNGGCWGCAG) and amplicon PCR reverse primer (GACTACHVGGGTATCTAATCC). Samples were amplified in duplicate with the following thermocycler protocol: 1 cycle of denaturing at 95°C for 3 min, first 5 cycles of denaturing at 95°C for 30 s, annealing at 45°C for 30 s, elongation at 72°C for 30 s, then 20 cycles of denaturing at 95°C for 30 s, annealing at 55°C for 30 s, elongation at 72°C for 30 s and a final extension at 72°C for 5 min. We used Hieff NGS DNA Selection Beads (Yeasen, 10105ES03, China) to purify the free primers and primer dimer species in the amplicon product. Samples were delivered to Sangon BioTech, Shanghai, for library construction using universal Illumina adaptor and index. Before sequencing, the DNA concentration of each PCR product was determined using a Qubit 4.0 Green double-stranded DNA assay and it was quality controlled using a bioanalyzer (Agilent 2100, USA). Depending on coverage needs, all libraries can be pooled for one run. The amplicons from each reaction mixture were pooled in equimolar ratios based on their concentration. Sequencing was performed using the Illumina MiSeq system (Illumina MiSeq, USA), according to the manufacturer’s instructions.

### 2.17. LC-MS/MS analysis

LC-HRMS analysis was performed on a Vanquish UPLC instrument coupled with a Q Exactive Orbitrap high-resolution mass spectrometer (Waltham, MA, USA). The chromatographic analysis was carried out on a Waters ACQUITY™ BEH C18 column (2.1 mm × 100 mm, 1.8 μm) under a 300 μL/min flow rate. Mobile phases A and B were H_2_O with 0.1% (v/v) formic acid and acetonitrile, respectively. A 22-min gradient was established as follows: 0–13 min, 20%B; 13–16 min, 20–100%B; 16–18 min. 100%B; 18–18.1 min, 100–20%B; 8.5–10 min, 5%B. The column temperature was maintained at 38°C. The samples were maintained at 4°C, and the injection volume was 2 μL. The mass spectrometer was equipped with an electrospray ionization source, and mass detection was operated in negative mode. The parameters were set as follows: spray voltage, 3.5 kV (+); capillary temperature, 350°C; sheath gas velocity, 35 arb; auxiliary gas velocity, 15 arb; sweep gas pressure, 0 arb; and S-lens RF level, 50 V. PRM was used to acquire targeted data.

### 2.18. Nano LC-LTQ-Orbitrap-MS/MS analysis

The samples were injected into a pre-column (Easy-column C18-A1, 100 μm × 20 mm, 5 μm, Thermo Fisher Scientific, USA), and peptide separation was performed on a reversed-phase C18 analytical column (Easy-column C18-A2, 75 μm × 100 mm, 3 μm, Thermo Fisher Scientific, USA) with a mobile phase composed of solvent A (0.1% formic acid in water) and solvent B (0.1% formic acid in acetonitrile) at a flow rate of 300 nL/min. The gradient elution program was applied as follows: 0–70 min, 2%–40% B; 70–75 min, 40%–95% B; 75–95 min, 95% B. MS analysis was conducted in positive ion mode under data-dependent acquisition (DDA). Full-scan MS spectra (*m/z* range: 350–2000 Da) were acquired in the Orbitrap with a resolution of 60,000 (*m/z* value: 400 Da). The top 15 most intense precursor ions were selected for MS/MS fragmentation via collision-induced dissociation (CID) with a normalized collision energy of 35 eV.

Raw data were processed using Proteome Discoverer 1.4 (Thermo Fisher Scientific, USA) with the Sequest HT algorithm against the *Ruminococcus gnavus* /*Clostridium butyricum* protein sequence database from Uniprot. Parameters included trypsin digestion (maximum two missed cleavages), carbamidomethylation of cysteine as a fixed modification, and methionine oxidation as a variable modification were searched. Precursor and fragment mass tolerances were set to 10 ppm and 0.8 Da, respectively. Identifications of protein and peptide was filtered at a 1% false discovery rate (FDR).

### 2.19. Expression of BpNfrA and StNfrA

The gene sequence of BpNfrA (Accession: CUO08416) and StNfrA (Accession: WP_084830667) was retrieved from the NCBI database and codon optimization was performed. The optimized gene was synthesized using the genomic DNA of *Bifidobacterium pseudocatenulatum* or *Streptococcus thermophilus* as a template, followed by restriction enzyme digestion and ligation into the pET-28a(+) expression vector to construct the recombinant plasmid. The recombinant plasmid was then transformed into *E. coli* BL21 (DE3) competent cells to generate recombinant strains. Antibiotic selection was performed by supplementing with kanamycin (50 μg/mL) under incubation in a shaking incubator at 37°C with 200 rpm agitation. Kanamycin-resistant colonies were expanded in fresh medium until the optical density (OD_600_) reached 0.6–0.8, at which point protein expression was induced by adding 0.5 mM isopropyl-β-D-thiogalactopyranoside (IPTG), followed by incubation at 16°C and 180 rpm for 20 hours. The bacterial cells were harvested by centrifugation at 4,000 rpm and 4°C for 30 minutes, resuspended in ice-cold protein-binding buffer (50 mM Tris, 0.5 M NaCl, pH 8.0), and lysed via sonication on ice. The lysate was centrifuged at 16,000 rpm and 4°C for 30 minutes, and the supernatant containing soluble proteins was collected for further purification.

The pre-packed affinity chromatography column (Ni column) was processed as follows: The Ni column was initially washed with 0.1 M EDTA (pH 8.0) to remove nickel ions bound to the agarose gel via chelation, followed by rinsing with 5 column volumes (CV) of double-distilled water (ddH₂O). Subsequently, the column was regenerated by flushing with 0.1 M nickel sulfate solution to allow re-adsorption of nickel ions onto the agarose gel, and then rinsed again with 5 CV of ddH₂O. Finally, the Ni column was equilibrated with 5 CV of protein binding buffer and pre-cooled at 4°C for subsequent use.

The supernatant was loaded onto the pre-cooled Ni column via a peristaltic pump to allow the His-tagged target protein to bind to the nickel-charged agarose gel resin. The column was then washed with five column volumes of protein-binding buffer and the flow-through collected. Small aliquots of the supernatant, centrifugation pellet, and flow-through were mixed with protein loading dye, denatured at 100°C, and prepared as samples for subsequent SDS-PAGE analysis. The protein-bound Ni column was then connected to an ÄKTA protein purification system for washing and elution. Competitive elution was performed using imidazole-containing buffers: Pump A delivered protein-binding buffer, while Pump B supplied elution buffer (1 M imidazole, 50 mM Tris, 0.5 M NaCl, pH 8.0). The eluted fractions were collected and then subjected to SDS-PAGE analysis to preliminarily assess target protein expression and purity.

### 2.20. Enzymatic kinetic assays

To determine the kinetic values of BpNfrA and StNfrA, reactions were performed with sennoside A concentrations ranging from 1.0 to 100 μM and 500 μM NADPH at PBS buffer (pH 5.0) in a total volume of 200 μl that containing 0.56 μM enzyme. A series of standard solutions of sennoside A ranging from 0.1 to 50 μM were prepared to calculate the consumption ratio of substrate and the enzymatic kinetic parameters. The reactions were conducted at 37°C for 10 min under anaerobic conditions. And then 4 times the volume of methanol was added to terminate the reaction and centrifuged at 12,000 rpm/min for 10 min. The supernatants were analyzed by high-performance liquid chromatography (λ = 260 nm). Data fitting was performed using GraphPad Prism 8, and *K*_m_ and *k*_cat_ values represent the mean of three independent replicates.

### 2.21. *Distribution of sennoside A in the co-incubation system of sennoside A and* E. coli

A blank control group without bacteria and an *E. coli*-sennoside A co-incubation group were set up, each with three biological replicates. A total of 900 μL of bacterial culture (with an initial OD₆₀₀ nm of 0.2) was mixed with 40 μL of a 2 mg/mL sennoside A solution in a 0.5% NaHCO₃ buffer solution, yielding a final sennoside A concentration of 100 μM.

The incubation system described above was cultured in an anaerobic incubator at 37°C with a nitrogen concentration exceeding 97% for 24 h. After incubation, 100 μL of the total bacterial culture was transferred to a new Eppendorf tube. Another 100 μL of the total bacterial culture was centrifuged at 8,000 rpm for 10 minutes to separate the supernatant and bacterial cells. The bacterial cells were washed with 500 μL of 0.1% DMSO-PBS. Subsequently, 100 μL of bacterial lysis buffer was added to both the total bacterial culture and the washed bacterial cells. The mixtures were vortexed to homogeneity and then sonicated in an ice-water bath to disrupt the cells. The resulting lysates were used for subsequent analyses.

The incubation and sample processing procedures, targeted *m/z* values, and mass spectrometry data analysis methods were all identical to those in Section 2.3.

## 3. Results

### 3.1. Sennoside A-reducing activity characterization of representative strains

**Figure 1.**
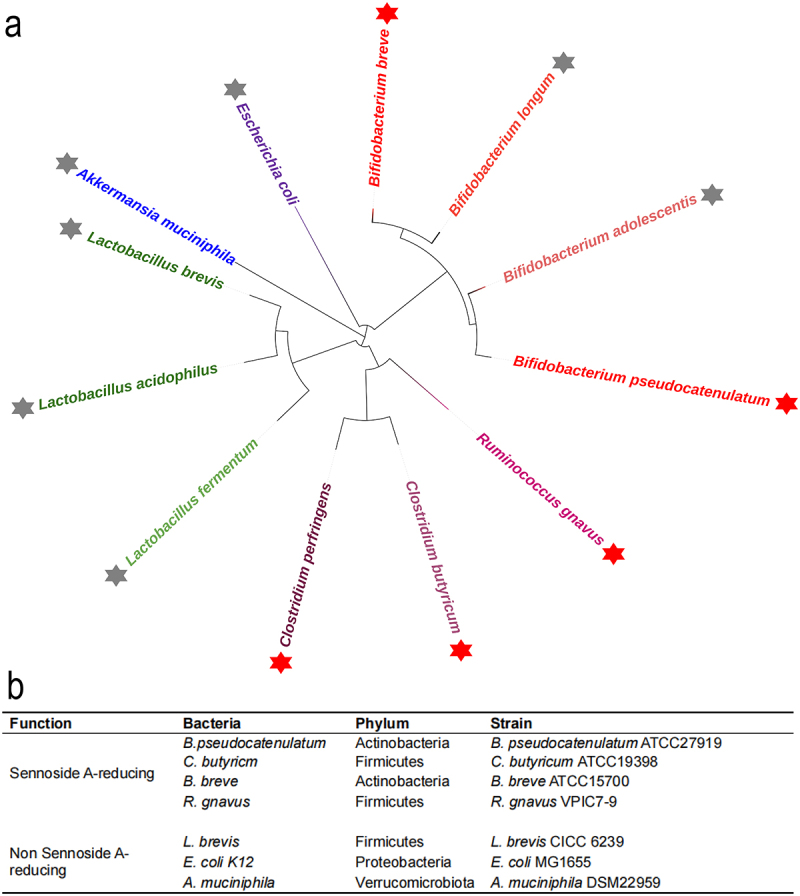
Selection of representative and model bacterial strains. (a) Phylogenetic tree of 12 representative gut bacterial strains tested for sennoside A metabolic activity. Five sennoside A-positive strains (red asterisks) and seven negative strains (gray asterisks) span multiple genera, including *Lactobacillus* (green), *Bifidobacterium* (red), *Clostridium* (purple), *Escherichia* (dark blue), *Akkermansia* (blue), and *Ruminococcus* (magenta). (b) The selected model bacterial strains. Abbreviations: *B. pseudocatenulatum*, *Bifidobacterium pseudocatenulatum*; *C. butyricum*, *Clostridium butyricum*; *B. breve*, *Bifidobacterium breve*; *R. gnavus*, *Ruminococcus gnavus*; *L. brevis*, *Lactobacillus brevis*; *E. coli*, *Escherichia coli*; *A. muciniphila*, *Akkermansia muciniphila*.

To investigate the relationship between the genetic background and metabolic activity of sennoside A-metabolizing strains, we established a phylogenetically diverse strain library comprising 12 representative gut microbes across six genera (Figure 1(a)). We applied LC-MS/MS to evaluate the sennoside A-reducing activity of these representative strains. The results showed that the five strains were capable of reducing sennoside A with high activity and defined as sennoside A-positive strains including *Bifidobacterium pseudocatenulatum*, *Bifidobacterium breve*, *Clostridium butyricum*, *Clostridium perfringens*, *Ruminococcus gnavus*, and the other seven strains were defined as sennoside A-negative strains with low activity (Figure S1-S3).^29^ Based on the phylogenetic analysis, seven strains were found to represent distinct clades or taxonomic units within the tree. Then, we selected above seven strains including four positive activity strains and three negative activity strains as the model bacterial strains (Figure 1(b)).

### 3.2. Biochemical characterization of sennoside A-reducing enzyme: BpNfrA as a case

**Figure 2.**
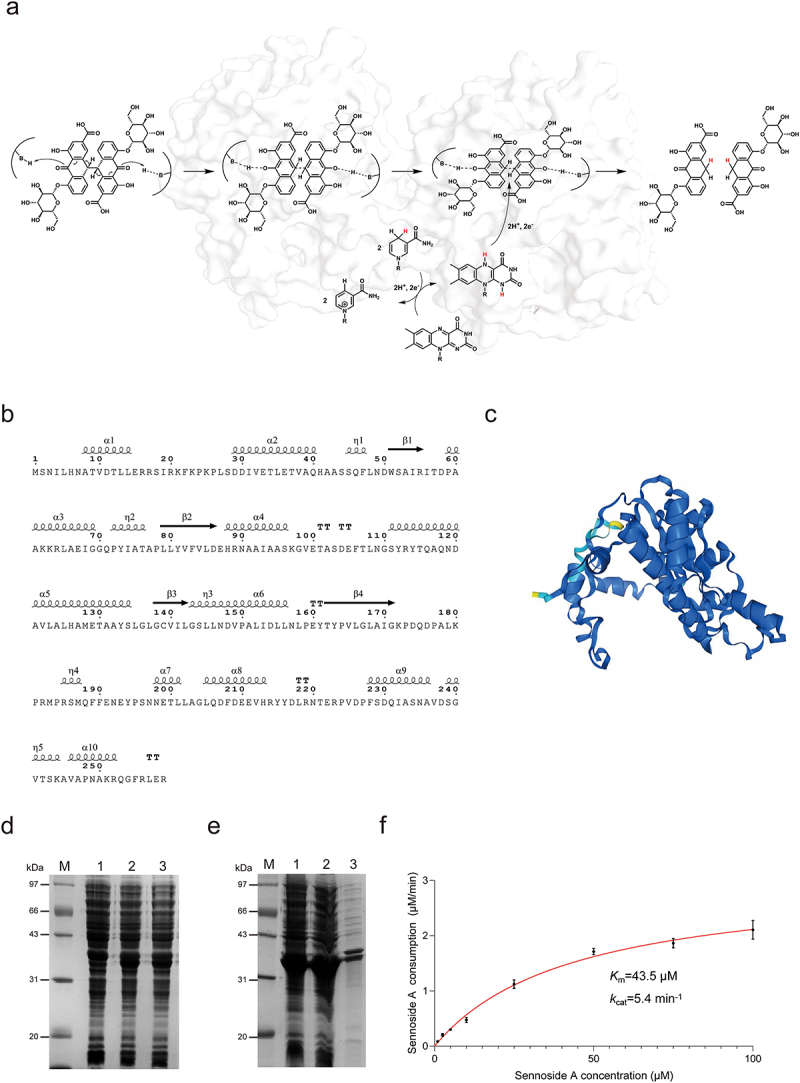
BpNfrA as a model enzyme for sennoside A reduction. (a) Proposed mechanism of the reductive cleavage of the C10–C10′ bond in sennoside A. (b) Amino acid sequence and secondary structure of BpNfrA. (c) Predicted 3D structure of BpNfrA by AlphaFold3. (d) Heterologous expression of BpNfrA by IPTG induction in *E. coli* BL21(DE3) cells. M, marker; 1, no IPTG; 2–3, IPTG added. (e) Soluble expression of BpNfrA. M, marker; 1, recombinant cells; 2, supernatant; 3, pellet. (f) Kinetics of BpNfrA-mediated reduction of sennoside A. *K*_m_ and *k*_cat_ were calculated from the mean ± s.D. Of three independent replicates (*n* = 3). Abbreviations: BpNfrA, NADPH-dependent nitroreductase from *Bifidobacterium pseudocatenulatum*; IPTG, isopropyl β-D-thiogalactoside.

**Figure 3.**
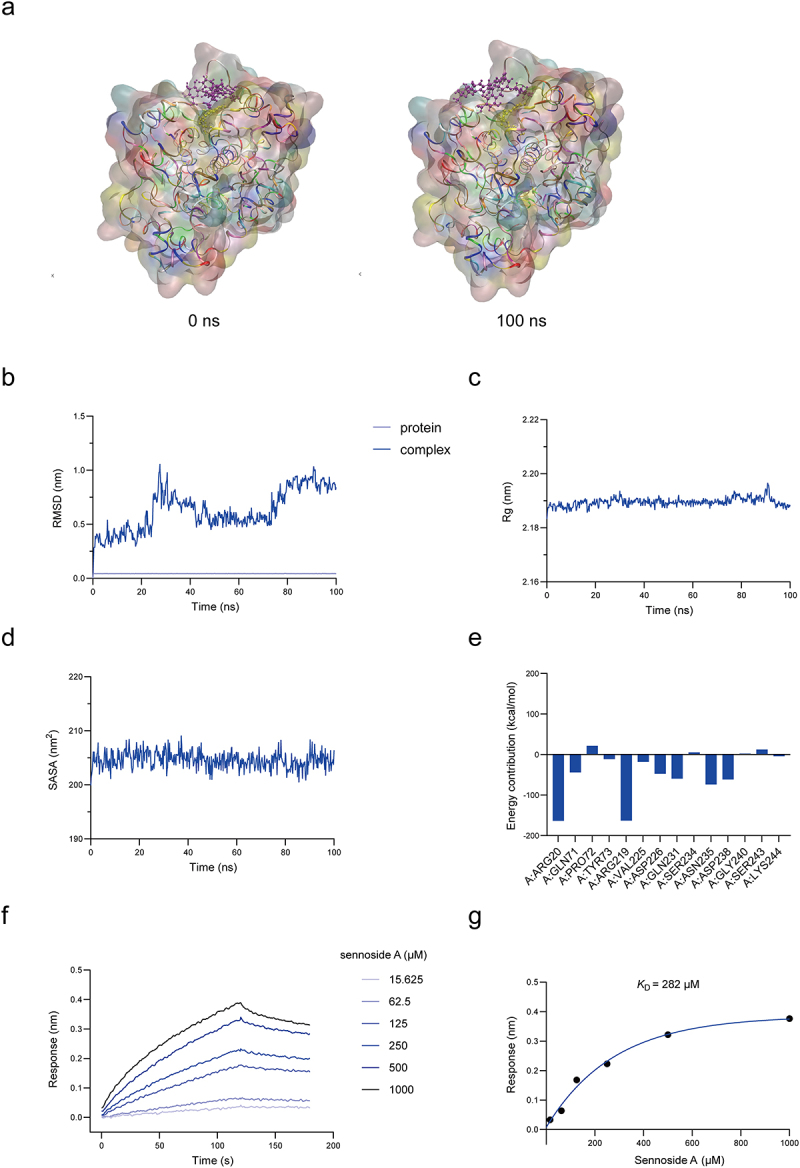
Characterization of direct binding between BpNfrA and sennoside A. (a) Conformation of sennoside A (purple) at 0 ns and 100 ns in MD simulations. (b) RMSD of the protein backbone and the complex. (c) Rg plot. (d) SASA plot. (e) Residue energy decomposition by the MM/GBSA method. (f) Sennoside A binding to BpNfrA detected by BLI. (g) *K*_D_ of sennoside A binding to BpNfrA. Abbreviations: BpNfrA, NADPH-dependent nitroreductase from *Bifidobacterium pseudocatenulatum*; MD, molecular dynamics; RMSD, root mean square deviation; Rg, radius of gyration; SASA, average solvent accessible surface area; MM/GBSA, molecular mechanics generalized born surface area; BLI, bio-layer interferometry; *K*_D_, equilibrium dissociation constant.

**Figure 4.**
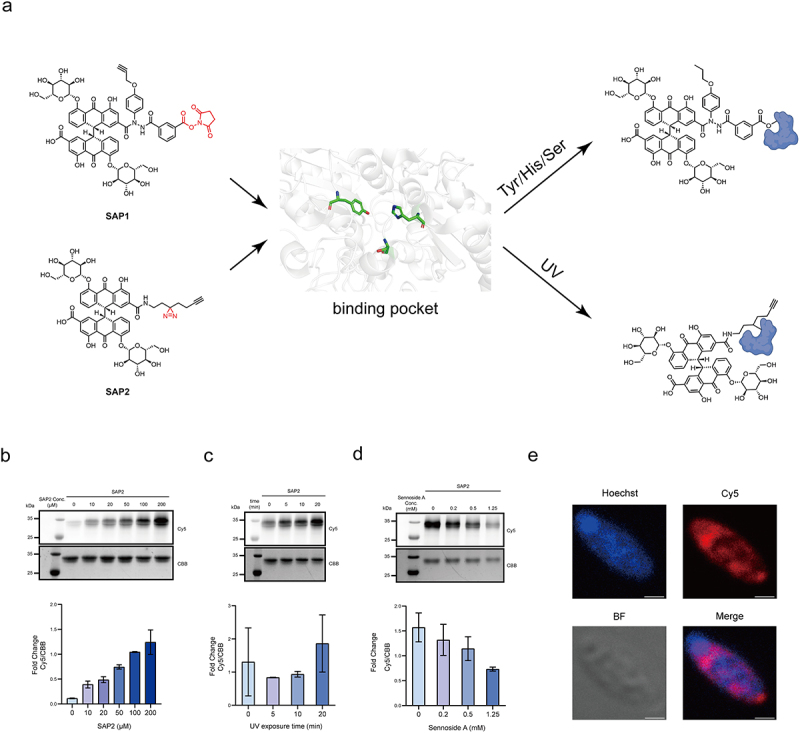
Design and validation of SAP probes for labeling model enzymes and bacteria. (a) Design strategy of SAP1 and SAP2. Schematic shows the binding pocket between SAP probes and the sennoside A-reducing enzyme. (b) Concentration-dependent labeling of BpNfrA by SAP2. (c) UV irradiation time-dependent labeling of BpNfrA by SAP2. (d) Substrate competition of SAP2. In b – d, labelling was analyzed by SDS-PAGE and fluorescence imaging. Fluorescent band intensity was quantified by ImageJ and normalized to CBB bands. Data represent mean ± s.D. (*n* = 3) (e) Confocal fluorescence imaging of *Clostridium butyricum* labeled with SAP2 (red) and Hoechst 33342 (blue). Scale bar, 1 μm. Abbreviations: tyr, tyrosine; his, histidine; ser, serine; UV, ultraviolet; CBB, Coomassie Brilliant Blue.

To investigate the specific mechanism of sennoside A metabolism by gut microbiota, we applied activity-based proteomic profiling to identify sennoside A-reducing enzyme in gut bacteria. Followed by protein purification, heterologous expression and reducing activity assays, we preliminary demonstrated that NfrA serves as the key enzyme responsible for sennoside A reduction in gut microbiota. To demonstrate that the function of sennoside A reduction by gut microbes is enzyme-dependent, we first reconfirmed the sennoside A-reducing activity of a previously identified nitroreductase, BpNfrA. The catalytic mechanism of BpNfrA cleaving the C10-C10’ bond of sennoside A was proposed based on the “ping-pong” model of flavin-dependent oxidoreductases (Figure 2(a)). The structure modeled by AlphaFold suggested that BpNfrA showed a characteristic pattern of flavin-dependent oxidoreductase, namely a Rossmann fold motif (Figure 2(b,c)). With purified recombinant BpNfrA in hand (Figure 2(d,e)), we found that BpNfrA reduced sennoside A to the product, rhein-8-*O-β*-D-glucoside (8GR), in an enzymatic manner, as *K*_m_ was 43.5 μM and *k*_cat_ was 5.4 min^−1^ (Figure 2(f)). With purified recombinant BpNfrA in hand (Figure 2(d,e)), MD simulations were performed to evaluate the structural stability and binding interactions of the protein-ligand complex. RMSD analysis revealed that the overall system stabilized after 40 ns, plus the Rg curve and the SASA curve, indicating a stable conformation during the remainder of the simulation (Figure 4(b–d)). Residue energy decomposition further identified key residues at the binding interface, including Arg20 and Arg219, which contributed strongly to the interaction energy, with binding free energy contributions of −163.43 kcal/mol and −73.97 kcal/mol, respectively (Figure 3(e)). These residues predominantly mediated electrostatic interactions with sennoside A. Bio-layer interferometry (BLI) analysis indicated that sennoside A directly bound to BpNfrA with a moderate affinity (*K*_D_ = 282 µM, Figure 3(g)). Collectively, these results demonstrated that BpNfrA can serve as a model enzyme for the reduction of sennoside A. Nitroreductase is widely present in gut microbes, and we hypothesize that NfrA has multiple isozymes expressed in different strains, and new methods are still required to search for the isozymes of BpNfrA and the functional bacteria.

### 3.3. Establishment and optimization of ABPs-FACS platform

#### 3.3.1. Design, synthesis, and characterization of ABPs

To identify sennoside A-reducing bacterial guilds in human gut microbiota *in situ*, we designed two sennoside A-based probes (SAP1 and SAP2) containing different reactive moieties. Previous studies have shown that there are serine and tyrosine distributed around the sennoside A binding site, so we designed SAP1, which introduces NHS groups that can covalently react with serine and tyrosine to achieve labeling (Figure 4(a)). Because of the risk of hydrolysis of NHS by intracellular esterases, we also designed SAP2, a simpler photoaffinity probe, which can cross-link to the C-H bonds of amino acid residues near the active site of sennoside A reductase through the photo-reactivity of diazirine (Figure 4(a)). In addition to the reactive group and sennoside A scaffold, both probes have a bioorthogonal group tags (alkyl handle) that allow the attachment of a reporter group (e.g., fluorophores or biotin) to the enzyme via a Cu(I)-catalyzed alkyne azide cycloaddition (CuAAC) reaction.

SAP1 was synthesized through a seven-step reaction route (Figure S4a). To introduce the reactive groups and alkyne group onto sennoside A, a compound containing a tetrazole group (compound 5) was first synthesized. The alkyne group and *N*-hydroxysuccinimide (NHS) moiety were then modified onto the tetrazole compound (compound 6). Finally, through a photocrosslinking reaction between the tetrazole group and the carboxyl group, the alkyne group and *N*-hydroxysuccinimide ester were attached to sennoside A. For SAP2, a one-step amide reaction between sennoside A and the amino group of 2-(3-(but-3-yn-1-yl)-3 H-diazirin-3-yl) ethan-1-amine was carried out to add azide and alkyne side chain onto the warhead via its carboxyl group (Figure S4b). SAP1 and SAP2 were characterized through ^1^H-NMR and HRMS (Supplementary information). Taken together, sufficient amount of SAP1 and SAP2 was prepared for further functional test.

#### 3.3.2. Investigation on the activity of SAP for labeling sennoside A-reducing bacteria guilds

In the design of the probes, the carboxyl group of sennoside A was chemically modified (Figure S5a). Before the labeling test, we first performed bacterial metabolic test and the result demonstrated the functional modification on the probe does not adversely affect their properties of substrate binding compared to the unmodified sennoside A (Figure S5b-e).

In order to confirm whether the probes could covalently label proteins within the sennoside A-reducing bacteria, we chose BpNfrA as model enzyme and *B. pseudocatenulatum* as model strain to exam the reactivity and selectivity of SAP1 and SAP2. First, the chemical labeling of SAP1 and SAP2 for BpNfrA was tested *in vitro*. In-gel fluorescence showed that SAP2 labels BpNfrA with UV- and dose-dependent manners while the labeling could be competed by sennoside A (Figure 4(b–d)). It demonstrated that SAP2 covalently labeled the model enzyme.

In order to visualize sennoside A-reducing bacteria, we attached fluorophores via CuAAC reaction at the end of the labeling reaction. We tested the selectivity and comprehensive application properties of SAP1 and SAP2 with a selection of different bacterial strains by laser confocal scanning microscopy. Both SAP1 and SAP2 could label active strain *B. pseudocatenulatum* (Figure S6, S7). However, when labeling inactive strain *L. brevis*, false positives were detected for SAP1, but not for SAP2 (Figure S8). The imaging of *C. butyricum* using SAP2 clearly marked the location of sennoside A at the scale of single cell (Figure 4(e)). Since SAP2 showed better imaging performance in live bacteria, SAP2 was selected for subsequent flow cytometry sorting.

To prove that SAP2 could specifically label the sennoside A reductase, we performed competition experiments in which *C. butyricum* was co-incubated with SAP2 and sennoside A. Relative quantification of SAP2 in the probe and competition groups using LC-HRMS was performed to analyze the consumption of SAP2 and the production of related metabolites over a 24-hour period. The results showed that SAP2 levels decreased in the probe group after 24 h compared with the control group (Figure S9a). The oxidative product rhein-8-*O-β*-D-glucoside-probe was detected in the probe group after incubation (Figure S9c). The results showed that the SAP2 could be metabolized by *C. butyricum*. However, the competition group exhibited higher levels of SAP2 compared to the probe group and revealed reductive metabolites of sennoside A, including rhein-8-*O-β*-D-glucoside and rheinanthrone-8-*O-β*-D-glucoside derivative (Figure S9a, b). The results showed that the addition of sennoside A in the competition group at 24 h inhibited SAP2 depletion. These findings suggested that the probe could specifically label the sennoside A reducing metabolizing enzyme in *C. butyricum*.

**Figure 5.**
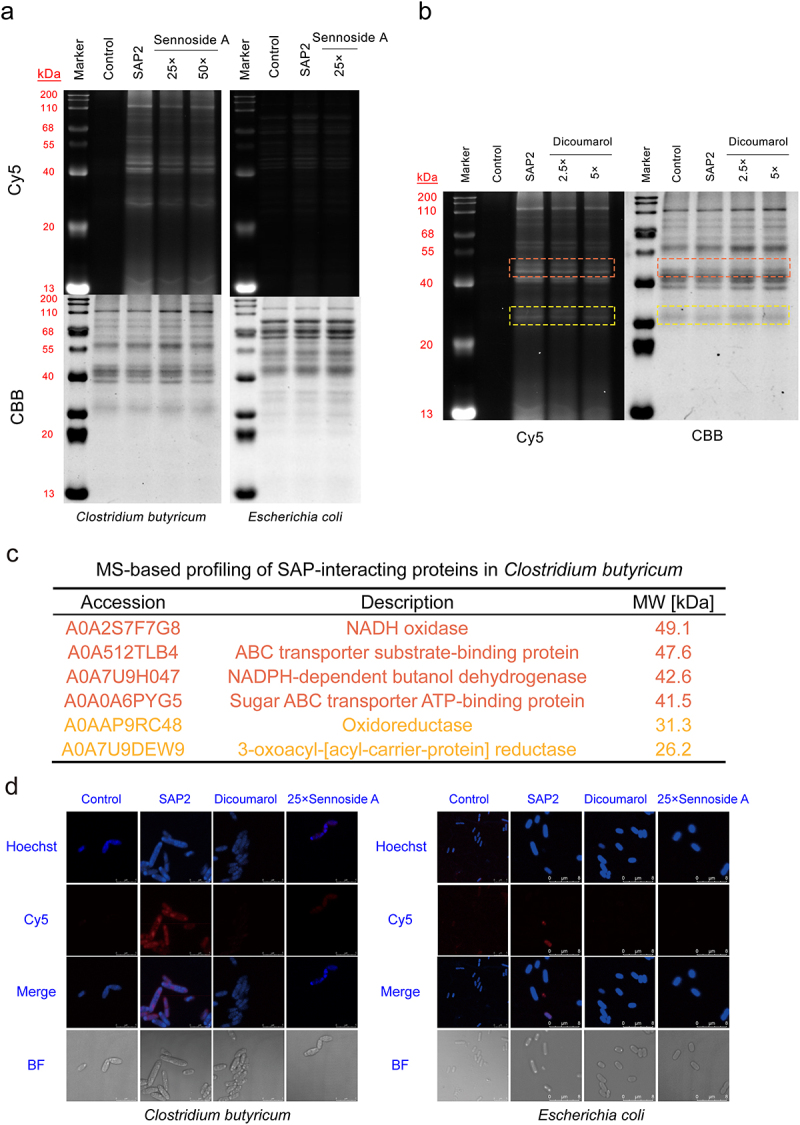
Cell-based validation of SAP2 probe targeting sennoside A reductase in *Clostridium butyricum*. (a) Gel-based profiling of SAP2-interacting proteins in *Clostridium butyricum* and *Escherichia coli* with sennoside A competition. (b) Gel-based profiling with dicoumarol inhibition. For (a) and (b), labeled samples were reacted with Cy5 and visualized by in-gel fluorescence scanning; CBB-stained gels confirm equal loading. Red and yellow boxes indicate protein bands (40–50 kDa and 25–35 kDa) with significant inhibition. (c) MS-based profiling of SAP2-interacting proteins in *Clostridium butyricum* by ABPP. Proteins identified in (a) and (b) were selected from ABPP results. (d) Confocal imaging of *Clostridium butyricum* and *Escherichia coli* labeled with SAP2 after inhibition/competition treatments. Scale bar, 10 μm. Abbreviations: CBB, Coomassie Brilliant Blue; MS, mass spectrometry; ABPP, activity-based protein profiling; BF, bright field.

To demonstrate SAP2 efficacy, we performed a live bacteria labeling experiment on *C. butyricum*. Bacteria were treated with SAP2, tagged with Cyanine 5, and analyzed by SDS-PAGE. Figure 5(a) showed that the fluorescence intensity in the Cy5 fluorescence channel was not proportional to the staining intensity of Coomassie Brilliant Blue, and it preliminarily indicated the selection of SAP2. To investigate the specificity of the SAP2 toward sennoside A reducing enzyme, bacteria were successively treated with sennoside A and SAP2, tagged with Cyanine 5, and analyzed by SDS-PAGE. The results showed that in the molecular weight ranges of 30–35 kDa and 42–55 kDa the protein bands had relatively low protein content (weak Coomassie Brilliant Blue staining) but bright labeling effects (strong fluorescence in the Cy5 channel). This indicates that proteins with these molecular weights might relate to the metabolism of sennoside A and have been labeled by SAP2. We also conducted SDS-PAGE experiments on *E. coli* (a negative control strain) using the same sample processing method. Figure 5(a) showed that the overall fluorescence intensity in *E. coli* was extremely weak.

In order to identify these SAP2 labeled proteins, we performed an activity-based protein profiling experiment. Combined with the results of SDS-PAGE, these SAP2 labeled proteins in *C. butyricum* were speculated to be oxidoreductases or ABC transporters (Figure 5c, Table S1). Among them, those with a molecular weight in the range of 30–35 kDa might be three different FAD-dependent oxidoreductases (uniprot: A0AAP9RI96, A0A2S7F6Y6, A0AAP9RC30), CoA-disulfide reductase (uniprot: A0A2S7F9Y6), and ABC transporter substrate-binding protein (uniprot: A0A512TLB4); those with a molecular weight in the range of 42–55 kDa might be Carbohydrate ABC transporter permease (uniprot: A0A3R9FCA6), Ferredoxin-NADP^+^ reductase subunit alpha (uniprot: A0A512TLG9), Oxidoreductase (uniprot: A0A2S7F8D0), and NAD(P)-dependent oxidoreductase (uniprot: A0A6L9EMM6). It is speculated that the oxidoreductases might be related to sennoside A metabolism and the ABC transporters might be related to the process of sennoside A entering the bacteria.

Since most of the speculated proteins are classified as flavin-dependent oxidoreductases, we conducted a dicoumarol-inhibited SDS-PAGE experiment to further confirm the above experimental results. Bacteria were successively treated with dicoumarol (flavin-dependent enzymes inhibitor) and SAP2 and tagged with Cyanine 5. Figure 5(b) showed that the corresponding protein bands of the relevant flavin-dependent enzymes were obviously dark. This result further confirmed that SAP2 is a selective probe. In addition, the results of laser scanning confocal microscopy experiment (Figure 5(d)) showed that SAP2 could enter *C. butyricum* to achieve the labeling of intracellular enzymes, while SAP2 could hardly enter *E. coli*. This also corroborated our previous experimental results. We also analyzed *R. gnavus* using the same method as that for *C. butyricum*, and similar experimental results were obtained (Figure S10, Table S2). The result of *R. gnavus* also confirmed that SAP2 is a selective probe.

Gene knockout mutants are the most direct approach to validate enzyme specificity. However, the strains we identified as metabolically active are currently not genetically tractable, making mutant construction technically challenging. Therefore, we selected representative strains listed in Figure 1(b) to validate probe metabolism through multiple complementary approaches, including gel electrophoresis, ABPP, and confocal fluorescence imaging. By analyzing the metabolic enzymes present in probe-positive strains, we demonstrated that the presence of the enzyme is essential for probe metabolism, thereby providing indirect evidence supporting the enzyme’s specificity.

In order to quantify the functional activity of sennoside A reductase in sennoside A-reducing bacteria guild, we optimized the labeling conditions to make the fluorescence intensity of the probe a real reflection of the *in situ* activity of the enzyme. The optimal labeling conditions were determined as follows: washing solvent, 2% BSA/PBS; probe concentration, 100 μM; incubation time, 1 h; Cy5 concentration, 5 μM (Figure S11).

### 3.4. Investigation on the selectivity of SAP2 for sennoside A-reducing bacteria in fluorescence-activated cell sorting (FACS)

To determine whether SAP2 could effectively distinguish sennoside A-reducing bacteria from bulk gut microbiota, we first performed ABP labeling followed by FACS on *B. pseudocatenulatum*. Bacterial isolates were incubated anaerobically at 37°C with or without 100 µM of SAP2 for 1 h. The bacterial cells were then fixed and underwent the click reaction with Cy5-azide. After conjugated to Cy5-azide, the bacterial cells were then subjected to FACS. In order to improve the signal-to-noise ratio, duplicates of each isolate incubated without probe were used as controls to determine the false-positive signals. The gating was set to exclude debris and doublets, which also contribute to false-positive signals. Compared to normal *B. pseudocatenulatum*, heat-treated *B. pseudocatenulatum* which underwent the labeling procedure showed lower fluorescence, demonstrating that only active bacteria are labeled using SAP2 (Figure S12).

**Figure 6.**
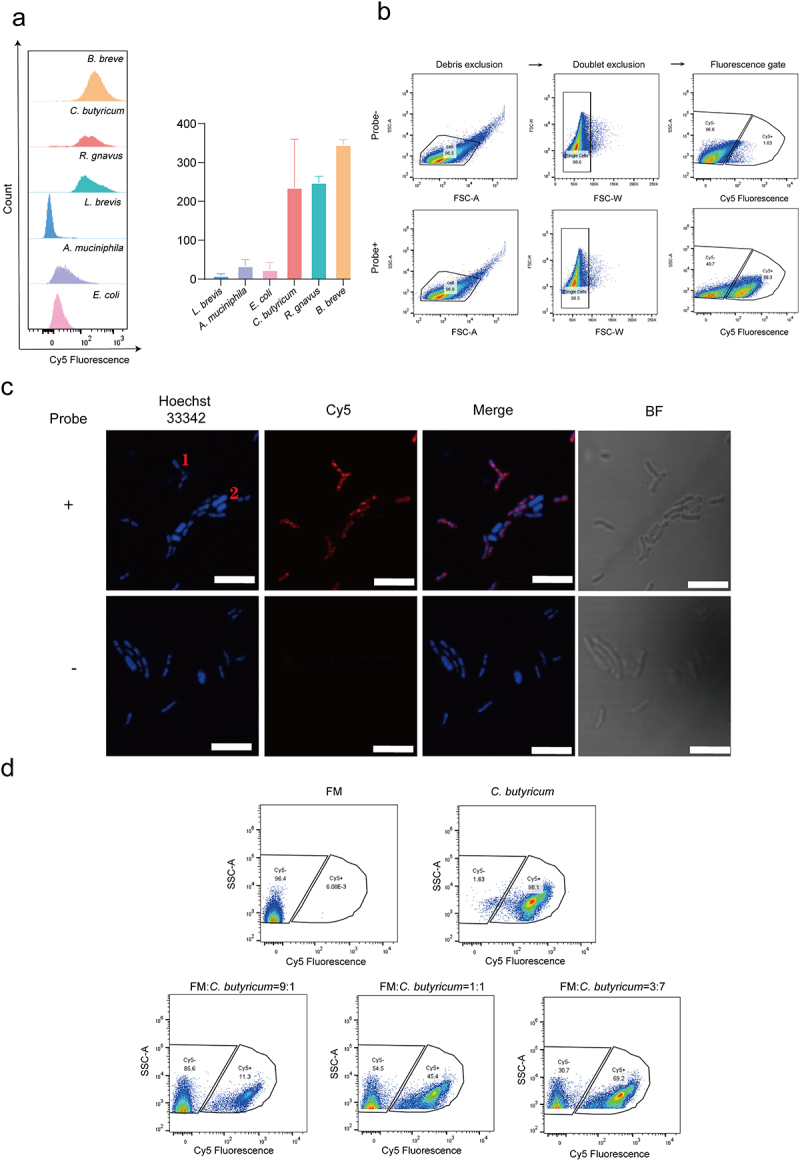
Flow cytometric and imaging analysis of SAP2 labeling in selected strains. (a) Flow cytometry of selected strains labeled with SAP2 (bars represent mean ± s.D., *n* = 2 biological replicates). (b) Flow cytogram of *Bifidobacterium breve* and *Escherichia coli* (1:1) with or without SAP2. Debris and doublets were excluded; Cy5^−^ and Cy5^+^ fractions were defined based on control incubations. (c) Confocal imaging of *Bifidobacterium breve* and *Escherichia coli* (1:1) labeled with SAP2 (red) and Hoechst 33342 (blue). 1: *Bifidobacterium breve*; 2: *Escherichia coli*. Scale bar, 5 μm. (d) Flow cytogram of various ratios of spiked-in *Clostridium butyricum* labeled with SAP2 in inactive fecal microbiota (FM). Abbreviations: *B. breve*, *Bifidobacterium breve*; *R. gnavus*, *Ruminococcus gnavus*; *L. brevis*, *Lactobacillus brevis*; *E. coli*, *Escherichia coli*; *C. butyricum*, *Clostridium butyricum*; *A. muciniphila*, *Akkermansia muciniphila*; BF, bright field; FSC-A, forward scatter area; SSC-A, side scatter area; FSC-H, forward scatter height; FSC-W, forward scatter width.

Second, we performed labeling on six bacterial strains, spanning five phyla and two kinds of bacteria cell wall. Individual detection of six bacterial strains revealed that the three positive strains exhibited strong fluorescence, while the three negative strains showed weak fluorescence. The proportion of probes labeled (Cy5^+^) against the three positive references, *C. butyricum*, *B. breve*, and *R. gnavus*, was 95.8%, 99.6%, and 99.2%, respectively; and the proportion of probes labeled against the 3 negative references, *A. muciniphila*, *E. coli*, and *L. brevis*, were labeled at 22.9%, 4.5% and 5.34%, respectively (Figure 6(a) and Figure S13, S14). We deduced that the fluorescence signals detected in non-metabolizing bacteria such as *L. brevis* and *E. coli* were caused by nonspecific labeling of the probes.

We next conducted the validation with a mock community composed of equal amounts of *B. breve* and *E. coli*. As it showed in the FACS plots, compared with the no probe group, after the addition of the probe, the bacterial cells were divided into two groups according to the difference in the strength of Cy5 fluorescence, and the ratio of the two groups of cells was roughly 1:1, which was in line with the initial mixing ratio of the strains, suggesting that probe labeling in conjunction with FACS sorting is capable of the isolation of sennoside A-metabolizing and non-metabolizing bacteria in complex systems (Figure 6(b)). Confocal fluorescence imaging results of the mock community revealed that only *B. breve* exhibited Cy5 fluorescence, while *E. coli* showed no detectable fluorescence, further confirming the labeling capability of SAP2 for SA-metabolizing bacteria (Figure 6(c)). To determine if SAP2 could be applied to human gut microbiota, further validation was performed using inactive fecal microbiota spiked with *C. butyricum*. It turns out that the established gating strategy reliably identified a pure population of sennoside A-reducing bacteria (Figure 6(d)). Collectively, these results verified the accuracy of our sorting procedure.

### 3.5. Identification of sennoside A-reducing bacteria in human gut microbiota

**Figure 7.**
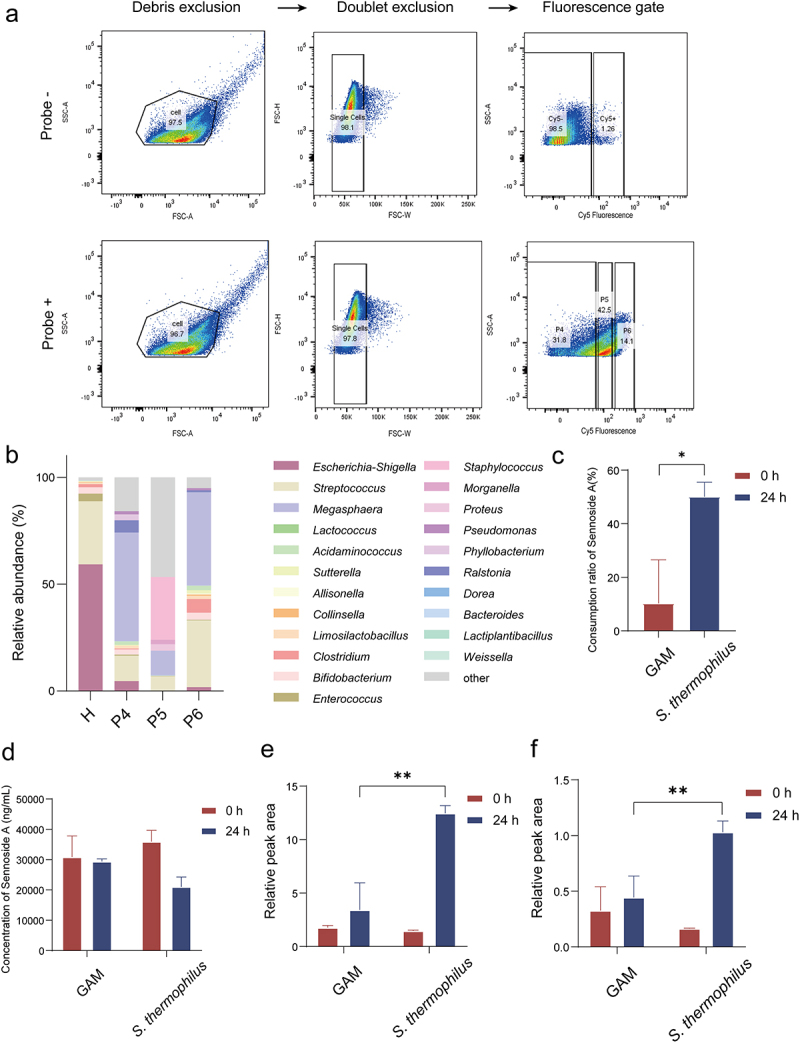
Discovery and validation of functional bacteria, *Streptococcus thermophilus*. (a) Flow cytogram of fecal microbiota with or without SAP2. Cy5^−^ and Cy5^+^ fractions were defined from control incubations; Cy5^+^ populations were further split into P6 (strong metabolizers, Cy5 fluorescence intensities > 120) and P5 (weak metabolizers, Cy5 fluorescence intensities < 120). (b) Relative abundance of each sorted fraction (H, P4, P5, P6) analyzed by 16S rRNA-amplicon sequencing at the genus level. H, initial fecal microbiota. (c) Sennoside A-reducing activity of *Streptococcus thermophilus* by substrate consumption ratio (*n* = 3). (d) Sennoside A-reducing activity of *Streptococcus thermophilus* by sennoside A concentration (*n* = 3). (e) Production of rhein-8-*O-β*-D-glucoside by *Streptococcus thermophilus* (*n* = 3). (f) Production of derivatization product rhein-anthrone-8-*O-β*-D-glucoside by *Streptococcus thermophilus* (*n* = 3). In c – f, data represent mean ± s.D. Abbreviations: FSC-A, forward scatter area; SSC-A, side scatter area; FSC-H, forward scatter height; FSC-W, forward scatter width; *S. thermophilus*, *Streptococcus thermophilus*. Statistical significance: *p < 0.05, **p < 0.01.

We then sought to identify sennoside A-reducing bacteria guilds in human gut microbiota using our method. Microbes isolated from the human feces were incubated with SAP2 or Cy5 only (‘No Probe’) under anaerobic conditions. The bacterial cells were fixed and sorted into Cy5^+^, Cy5^−^, and bulk cell populations. In the previous study, it was found that the fluorescence intensity of *B. breve* was stronger than the rest of the metabolizing bacteria (Figure 6(a)), suggesting that there was correlation between the fluorescence intensities and their ability to metabolize sennoside A. As for the gate-setting, we defined the subsets where 95% of the cells in the Cy5^−^ group presented as P4 gate, and further divided the positive subsets in the Cy5^+^ group into strong-metablizers (P6 gate) and weak-metabolizers (P5 gate) based on fluorescence intensity. Results showed 31.8% of the bacterial cells were in the P4 gate, 42.5% within the P5 gate and 14.1% within the P6 gate (Figure 7(a)).

To identify the sennoside A-metabolizing microbes, community composition was determined for each population by 16S rRNA gene amplicon sequencing, and differentially abundant operational taxonomic units (OTUs) were identified (Figure 7(b)). Increased abundance of Bacilli and decreased abundance of Negativicutes were observed in P5 gate and P6 gate compared to P4 gate. In the P5 gate and P6 gate, the composition of classes was similar, with higher abundance of Cyanobacteria found in P5 gate and higher abundance of Clostridia found in P6 gate. Ten OTUs with statistically enriched abundance in the Cy5^+^ population compared to the bulk population were considered sennoside A-reducing bacteria guild, spanning five phyla: Clostridia (three OTUs), Negativicutes (two OTUs), Gammaproteobacteria (one OTUs), Bacilli (two OTUs), and Bacteroidia (two OTUs). Additionally, OTUs with significantly enriched abundance in the Cy5^−^ compared to the bulk population were considered sennoside A-none-reducing bacteria guild. Seven OTUs were enriched in Cy5^−^, spanning three phyla: Firmicutes (four OTUs), Proteobacteria (two OTUs), and Bacteroidota (one OTUs). The results indicated that probe labels gut bacterial isolates with no bias based on cell wall type.

At genus level, *Clostridium*, *Streptococcus*, *Bacteroides* and *Limosilactobacillus* were of higher abundance in Cy5^+^ (P5 and P6), and *Escherichia-Shigella* was of higher abundance in Cy5^−^ (P4). Interestingly, *C. butyricum* is the model strain of *Clostridium*, which was confirmed to show the sennoside A metabolizing activity through *in vitro* incubation.^29^ In addition to *Clostridium*, *Streptococcus, Bacteroides* and *Limosilactobacillus* were enriched in P6 gate, indicating potential sennoside A-reducing bacteria may be found in these two genera. We analyzed the prevalence of *Bacteroides*, *Streptococcus* and *Limosilactobacillus* in the database and found that *S. thermophilus, L. reuteri, B. plebeius and B. ovatus* showed high prevalence in the human intestinal tract.^34^

We performed anaerobic incubation of pure isolates with sennoside A. The consumption of sennoside A and the production of metabolites were measured by LC-MS/MS. *S. thermophilus* significantly depleted sennoside A after 24 h of incubation compared to the control group without bacteria (Figure 7(c)). Besides, rhein-8-*O-β*-D-glucoside and the derivatization product of rhein-anthrone-8-*O-β*-D-glucoside appeared after incubation, indicating that sennoside A was reductively cleaved by *S. thermophilus* (Figure 7(d–f)). It was shown that sennoside A is converted to its active form rhein anthrone due to *β*-glucosidase activity and C–C reductase activity of gut bacteria.^35^ However, we didn’t detect rhein anthrone and its derivatization product in *S. thermophilus* suggesting the activity of glucosidase was weak.

We conducted experiments in which *L. reuteri, B. plebeius*, and *B. ovatus* were incubated with sennoside A for 24 h. LC-MS/MS was performed to quantify the consumption of sennoside A and the production of related metabolites in the three incubation systems over 24 h. These results showed that all three strains significantly consumed sennoside A after 24 h of incubation compared with the control group, and all of them detected the production of sennoside A metabolites such as rhein, rhein-8-*O-β*-D-glucoside, rheinanthrone derivatives and rheinanthrone-8-*O-β*-D-glucoside derivatives (Figure S15–17). Collectively, these data indicated that the three strains possess the capacity for reductive metabolism of sennoside A.

### 3.6. Validation of the mechanism based on the enzymatic activity: S. thermophilus as a case

**Figure 8.**
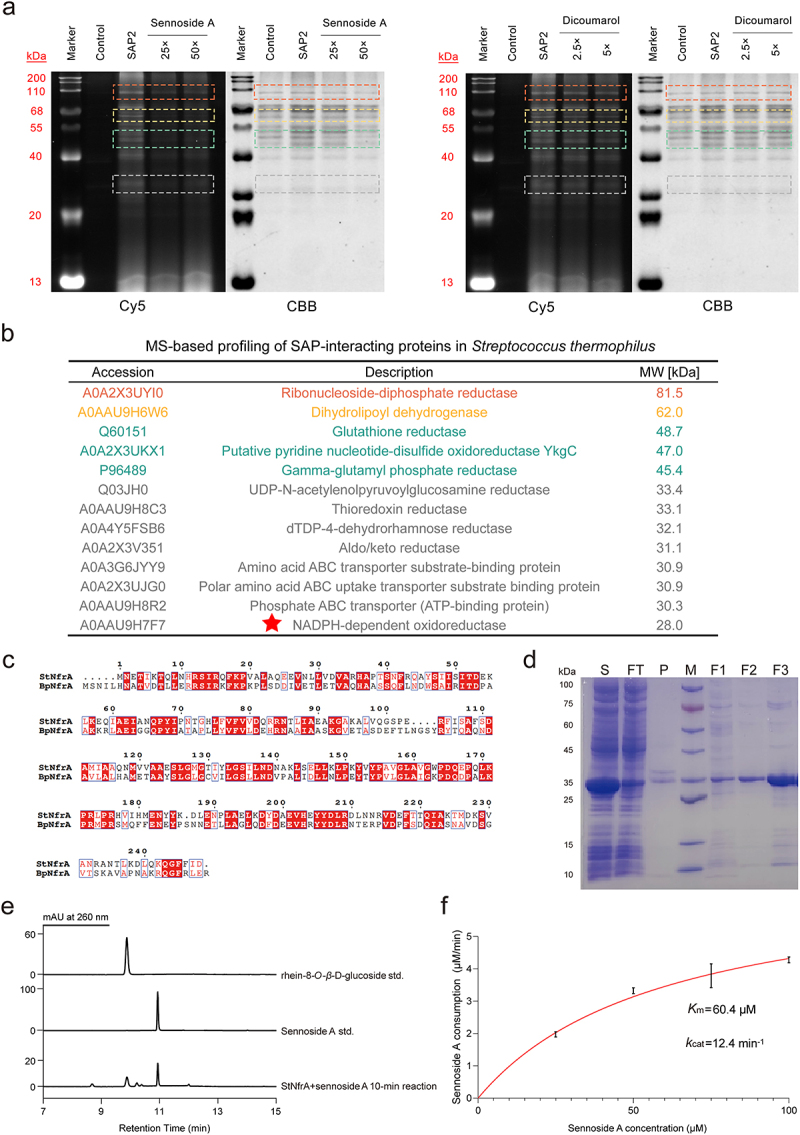
Identification and validation of sennoside A reductase StNfrA in *Streptococcus thermophilus*. (a) Gel-based profiling of SAP2-interacting proteins in *Streptococcus thermophilus* with sennoside A competition and dicoumarol inhibition. CBB-stained gels confirm equal loading. Bands with significant inhibition are highlighted (red: 80–100 kDa; yellow: 60–65 kDa; green: 45–50 kDa and 25–35 kDa). (b) MS-based profiling of SAP2-interacting proteins in *Streptococcus thermophilus* by ABPP. Proteins in four molecular weight ranges were selected based on (a). (c) Sequence alignment of StNfrA and BpNfrA. (d) SDS-PAGE analysis of heterologous expression and purification of StNfrA. S, supernatant; FT, flow-through; P, pellet; M, marker; F1–3, fractions of interest. (e) Representative chromatogram of StNfrA enzymatic assays. Top: standard rhein-8-*O-β*-D-glucoside; middle: standard sennoside A; bottom: reaction products after incubation with StNfrA and sennoside A. (f) Kinetic analysis of StNfrA activity. *K*_m_ and *k*_cat_ values are shown as mean ± s.d. (*n* = 3). Abbreviations: 8GR, rhein-8-*O-β*-D-glucoside.

**Figure 9.**
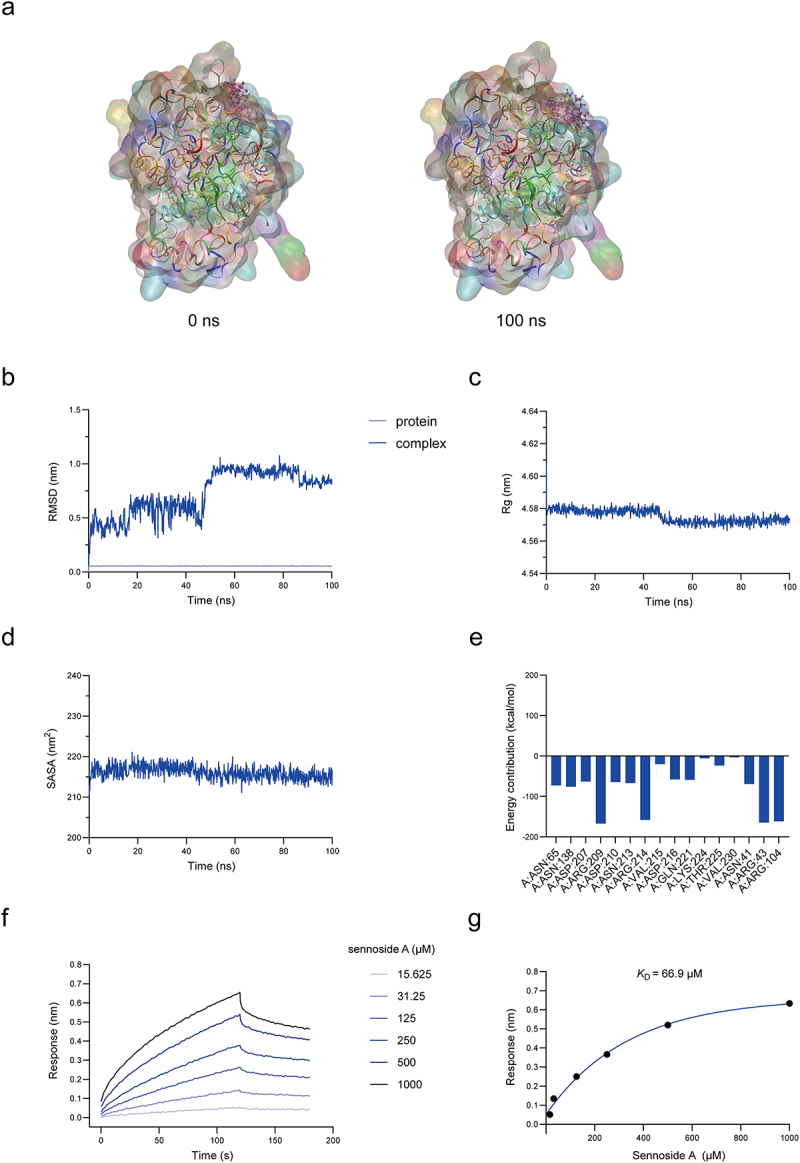
Characterization of direct binding between StNfrA and sennoside A. (a) Conformation of sennoside A (purple) at 0 ns and 100 ns in MD simulations. (b) RMSD for the protein backbone and complex. (c) Rg plot. (d) Average SASA plot. (e) Residue energy decomposition by the MM/GBSA method. (f) Sensorgram of sennoside A binding to StNfrA detected by BLI. (g) *K*_D_ of sennoside A binding to StNfrA. Abbreviations: StNfrA, NADPH-dependent nitroreductase from *Streptococcus thermophilus*; MD, molecular dynamics; RMSD, root mean square deviation; Rg, radius of gyration; SASA, average solvent accessible surface area; MM/GBSA, molecular mechanics generalized born surface area; BLI, bio-layer interferometry; *K*_D_, equilibrium dissociation constant.

To demonstrate that the functional bacteria identified using this method indeed contain sennoside A-reducing enzymes, we selected *S. thermophilus* as a case for validation. We analyzed *S. thermophilus* using the same method as that for *C. butyricum*, and similar experimental results were obtained. Figure 8(a) showed that the fluorescence intensity in the Cy5 fluorescence channel was not proportional to the staining intensity of Coomassie Brilliant Blue. Sennoside A competition SDS-PAGE experiment showed that the proteins with molecular weight ranges of 80–100 kDa, 60–65 kDa, 45–50 kDa, and 25–35 kDa had relatively low protein content but bright labeling effects. Combined with the results of ABPP (Table S3), these SAP2 labeled proteins in *S. thermophilus* were speculated to be oxidoreductases or ABC transporters. The possible information about these labeled proteins has been listed in Figure 8(b), and we found that the selected mode enzyme NfrA was also included. The dicoumarol inhibition SDS-PAGE experiment further showed that the corresponding protein bands of the relevant flavin-dependent enzymes were obviously dark. In addition, to discover the isozymes of BpNfrA in *S. thermophilus*, BLASTp was also performed, and a homolog which had the identity of 45% with BpNfrA was found, which shared structural homology with BpNfrA as well (Figure 8(c), S18). We therefore named it as StNfrA and obtained the recombinant protein (Figure 8(d)). To confirm the reductive activity of StNfrA, we performed anaerobic incubation of StNfrA with sennoside A and NADPH. The consumption of sennoside A and the production of rhein-8-*O-β*-D-glucoside (8GR) were measured by HPLC (Figure 8(e)). StNfrA reduced sennoside A in an enzymatic manner, *K*_m_ was 60.4 μM, *k*_cat_ was 12.4 min^−1^ (Figure 8(f)). MD modeling provided detailed insights into the structural basis for their catalytic mechanisms (Figure 9(a)). The RMSD analysis demonstrated that the system reached equilibrium after approximately 50 ns, maintaining an average RMSD of ~ 1.0 Å for the remainder of the simulation (Figure 9(b)). Together with the Rg curve and the SASA curve, it indicates that the complex retained a stable conformation throughout (Figure 9(c,d)). Residue energy decomposition analysis pinpointed key residues at the binding interface, such as Arg43, Arg104, Arg209, and Arg214, as major contributors to binding affinity. Further BLI analysis validated that sennoside A showed strong binding to StNfrA (*K*_D_ = 66.9 µM, Figure 9(g)). The mechanism by which StNfrA catalyzes sennoside A presented here provides a model for studying the enzymatically reductive cleavage of sennoside A in the gut. Moreover, by confirming the activity of StNfrA, these results further support that the ABP-FACS method we established in this study could efficiently discover functional bacteria.

## 4. Discussion

The metabolic activation of sennoside A, a well-known laxative agent, was conducted by bacterial reductases in the gut. However, current discovery methods like strain screening rely on ex vivo culturing, which is deprived of the symbiosis of the community, rendering the functional guild that responsible for reducing sennoside A difficult to recognize. In our previous study, preliminary processes have been developed whereby nitroreductases contribute to the degradation of sennoside A as C-C reductive cleavage enzymes. Here we proposed and proved that a nitroreductase from *Bifidobacterium pseudocatenulatum*, BpNfrA, exhibited the catalytic activity for sennoside A reduction. This was the first bacterial model enzyme being characterized in the metabolism of sennoside A. The MD results unveiled the catalytic mechanism and identified critical binding residues that could serve as targets for further optimization. Previous research indicated that *E. coli* nitroreductases could transform berberine into dihydroberberine through a reductive mechanism.^36^ Dihydroberberine was proved to exhibit higher lipophilicity and better absorption compared to berberine, which addressed the low bioavailability of the parent compound. In addition to berberine, again, the metabolic conversion of sennoside A highlights the functional importance of nitroreductases in modulating the pharmacokinetics of natural products.

Photoaffinity groups have unparalleled advantages in the discovery studies of functional intestinal bacteria. Photoaffinity groups are potential reactive groups, produces highly active carbene intermediates only upon activation by specific UV light. Then it could covalently react with metabolic enzymes and nearby amino acid residues inside the functional bacteria. The photoaffinity labeling could also be used as a signal amplification strategy. Due to the broad-spectrum nature of the photoaffinity group, it could increase the amount of probe labeling within the bacteria, which in turn increases the fluorescence intensity of the strains. Then we could use the difference of fluorescence intensity to isolate the bacteria. However, due to the low specificity of the photoreactive group, it will be the nonspecific labeled during the reaction. A weak fluorescence signal showed in a few non-metabolizing bacteria such as strains of *L. brevis* and *E. coli* may be due to nonspecific labeling of the probes. The fluorescence intensity of sennoside A-metabolizing bacteria was still significantly different from that of non-metabolizing bacteria, providing a basis for distinguishing based on the fluorescence intensity of the strains. We also found that the Cy5 fluorescence intensities of the four sennoside A-metabolizing bacteria were different, with the strongest fluorescence from *B. breve* followed by *C. butyricum* and *B. pseudocatenulatum*, suggesting that the fluorescence intensity of our probe to the labeled bacteria may be related to the sennoside A metabolism ability of the strains.

In our study, we observed an interesting phenomenon that SAP2 failed to labeling *E. coli* (Figure 5(a,d)), while the canonical nitroreductase NfsB from *E. coli* has been studied extensively. This suggests that despite the presence of nitroreductase in *E. coli*, other factors of bacteria such as physiological or structural characteristics may prevent its metabolic activity toward sennoside A. To investigate this mechanism, we co-incubated *E. coli* with sennoside A and monitored sennoside A consumption. After incubation, no significant reduction of sennoside A was detected in the total culture (Figure S3), which is consistent with its non-metabolizing phenotype as confirmed by our probe-based assays (Figure S19). Subsequent separation of the incubation system into supernatant and total culture revealed that sennoside A mostly remained in the supernatant and displayed minimal intracellular accumulation. This indicates that surface membrane proteins of *E. coli* prevent the sennoside A absorption, thereby leading to the non-metabolizing phenomenon.

These findings demonstrated that the enzyme serves as a necessary but not sufficient element for sennoside A metabolic capability in bacterial strains. Strains lacking the enzyme invariably lose activity, while those harboring the enzyme may still remained inactive (Figure 5(a,d)). Consequently, assessing functional activity based solely on bioinformatic detection of enzymatic presence is unreliable. Our enzymatic activity visualization platform employed the fluorescence intensity of probes to indicate bacterial strain activity, making it a robust tool for screening functional strains. Our probes processed compact size and the functional group modifications do not compromise the substrate’s intrinsic activity. Additionally, the characteristics of *E. coli* suggest that transmembrane transport proteins on the bacterial surface are a prerequisite for a strain to uptake and metabolize compounds to become functional bacteria. Therefore, constructing active probes to identify and study these transmembrane proteins, as well as deciphering their roles, will be critical for study on gut functional bacteria.

For microbiome characterization, probe-assisted fluorescence-activated cell sorting (FACS) is a powerful tool for characterizing the functional capacity of the microbiome at the cell scale. The biggest advantage of this method is that it provides the possibility of *in situ* measurement. To better simulate the natural environment of microbial communities, we created artificial microbiota using model strains, which included a mix of one metabolic bacteria (*B. breve*) and one non-metabolic bacteria (*E. coli*). Furthermore, to tailor our method for application in human gut, we combined inactivated human gut bacterial suspensions with different ratios of probe-labeled *C*. *butyricum*. The results showed that our method can effectively isolate sennoside A metabolic bacteria from a complex background. However, due to the dependence of the probe on the *in vitro* incubation process for microbial community labeling, it may disrupt the original ecological environment of the microbial community. Despite our efforts to replicate the intestinal environment, this incubation step may still lead to changes in the community structure.

The conversion of sennoside A to the laxative component rhein anthrone was carried out by two pathways, with two types of enzymes involved in the metabolic conversion reaction, a reductive metabolism enzyme responsible for breaking the C10-C10’ bond, and a *β-*glucosidase responsible for the hydrolysis reactions to produce rheinanthrone and rhein. Interestingly, either derivative of rheinanthrone or rhein, which are the products of glucosidase, were detected in the 24-h incubation system of *S. thermophilus* and *L. reuter*, indicating that the activity of glucosidase in these strains was weak. The above results indicated that the intestinal flora was a complex community, in which not only one type of intestinal bacteria was involved in the metabolism of exogenous drugs, but also several types of intestinal bacteria metabolized the drugs using multiple metabolic enzymes in them. Therefore, the use of active probes targeting a single metabolic enzyme to identify functional bacteria is not comprehensive, and the broad-spectrum nature of the photoaffinity probes gives them an edge on achieving broad-spectrum detection of functional bacteria.

The ABP-FACS method was further demonstrated by the identification of *S. thermophilus* as a sennoside A-metabolizer and StNfrA as a sennoside A reductase. Interestingly, *S. thermophilus* is a widely used probiotic in the manufacture of yogurts. Mechanistic investigations suggested that sennoside A had better binding effect with StNfrA than with BpNfrA, which consisted with the enzymatic activity experiment results. The discovery of StNfrA as a key enzyme in the metabolism of sennoside A opened new avenues for improving the bioavailability and therapeutic potential of this widely used natural product. Continued research in this area will deepen our understanding of the interplay between sennoside A, gut microbiota, and host pharmacology, paving the way for innovative approaches in drug development.

Overall, the application of *in situ* imaging techniques for gut bacteria significantly improves the accuracy of identifying drug-metabolizing bacteria due to two key advantages. First, *in situ* imaging allows researchers to directly observe bacterial activity at the cellular or subcellular level within their native environment. This is crucial because bacterial behavior, especially in response to drugs, is highly dependent on the physiological conditions and spatial organization of the microbial community. By avoiding artificial culturing or isolation steps, *in situ* imaging ensures that the observed responses to the drug are more representative of the bacteria’s natural state, reducing false positives or negatives in functional screening. This not only broadens the scope of bacteria that can be studied but also ensures that previously overlooked functional bacteria are identified. Moreover, the activity-based probe offers high sensitivity and specificity for detecting drug-metabolizing bacteria. The intensity of fluorescence signals provides quantitative data on the activity of bacteria, which correlates with the level of enzymatic activity, allowing for fast and precise identification without interference from nonfunctional bacteria. In the future, the enzymatic activity visualization method developed in this study can serve as a useful chemical tool for the discovery of drug metabolizing enzymes as well as their host organisms in the gut.

## Supplementary Material

Table S3.xlsx

Table S1.xlsx

Table S2.xlsx

supplementary_material.doc

## Data Availability

The authors confirm that the data supporting the findings of this study are available within the article and its supplementary materials. Source data and reagents are available from the corresponding author on reasonable request.
