# Supplementary material for: In situ identifying sennoside A-reducing bacteria guilds in human gut microbiota via enzymatic activity visualization
Source: Gut Microbes. 2025 Sep 25;17(1):2560598. doi: 10.1080/19490976.2025.2560598 (PMC12477872; doi:10.1080/19490976.2025.2560598)
Supplement: supplementary_material.doc [file KGMI_A_2560598_SM6587.doc]

**Supplementary material**

***In situ* identifying sennoside A-reducing bacteria guilds in human gut microbiota via** **enzymatic activity visualization**

Chuanjia Zhai ^a,#^, Xinyue Liu ^a,#^, Zhen Liu ^a,#^, Huilin Ma ^a^, Huajinzi Li ^a^, Yingxi Gong ^a^, Xiang Li ^a^, Yingyue Wang ^a^, Na Zhang ^a^, Han Zhang ^a^, Gan Luo ^a^, Ying Wang ^a^,*, Xiaoyan Gao ^a^,*

^a^ School of Chinese Materia Medica, Beijing University of Chinese Medicine, Beijing, 102488, China.

^#^ These authors contributed equally to this work.

*Correspondence:

Xiaoyan Gao

School of Chinese Materia Medica, Beijing University of Chinese Medicine, South of Yangguang Street, Fangshan District, Beijing, 102488, P. R. China.

E-mail address: [gaoxiaoyan@bucm.edu.cn](mailto:gaoxiaoyan@bucm.edu.cn)

Ying Wang

School of Chinese Materia Medica, Beijing University of Chinese Medicine, South of Yangguang Street, Fangshan District, Beijing, 102488, P. R. China.

E-mail address: wangy174@126.com


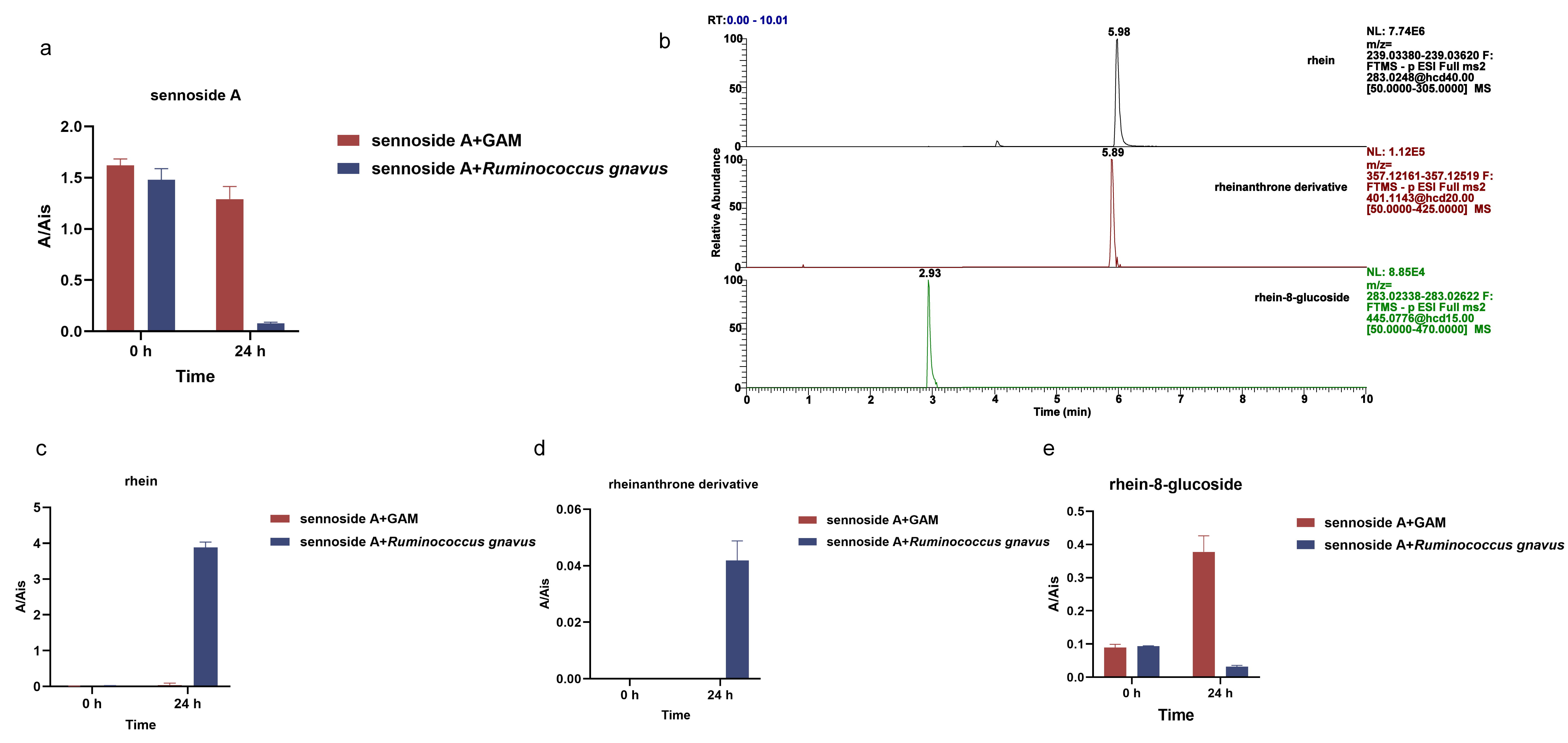


**Figure S1. Identification of sennoside A reduction products by *Ruminococcus gnavus*.**

(a) Sennoside A-reducing activity of *Ruminococcus gnavus* assessed by peak area of sennoside A using LC-MS/MS. (b) Extracted ion chromatograms (EICs) of oxidized and derivative products from sennoside A metabolism. (c–e) Quantification of rhein (c), rheinanthrone derivative (d), and rhein-8-glucoside (e) in the system. In (a) and (c–e), data represent mean ± s.d. (*n =* 3).


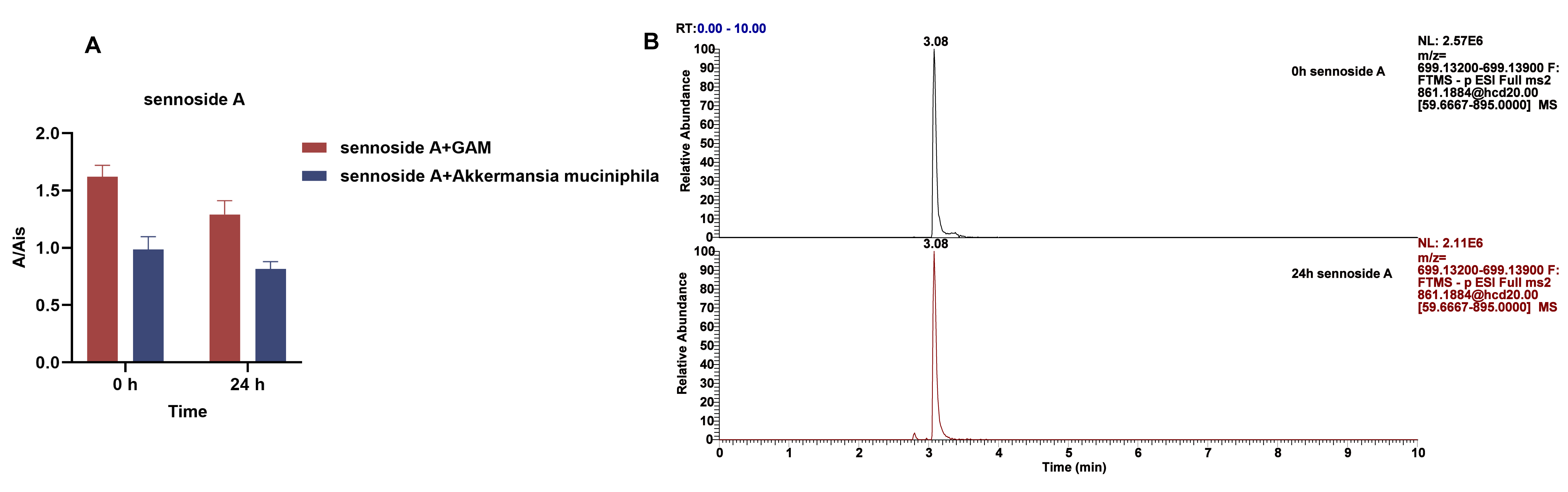


**Figure S2. Sennoside A-reducing activity of *Akkermansia muciniphila*.**

(a) Sennoside A peak area after incubation with *Akkermansia muciniphila*, as determined by LC-MS/MS. (b) EICs of sennoside A in samples incubated with *Akkermansia muciniphila* for 0 h (top) and 24 h (bottom). In (a), data represent mean ± s.d. (*n =* 3).


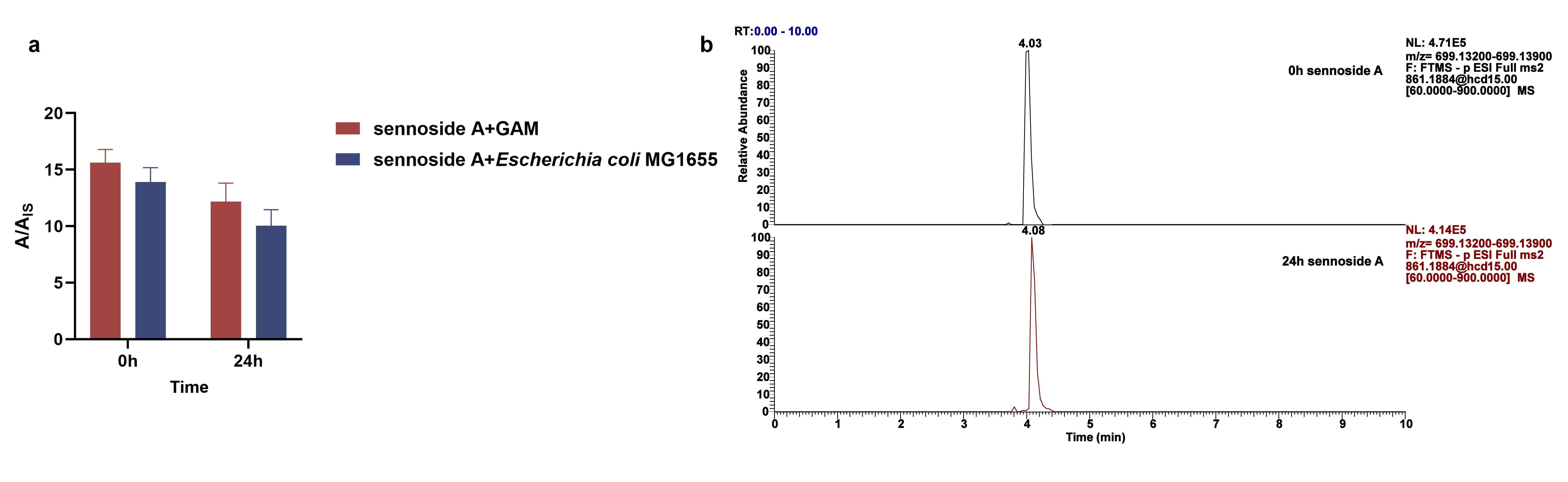


**Figure S3. Metabolism of sennoside A by *Escherichia coli* MG1655.**

Extracted ion chromatograms (EICs) of sennoside A in samples incubated with *Escherichia coli* MG1655 for 0 h (top) and 24 h (bottom), analyzed by LC-MS/MS. Data represent mean ± s.d. (*n =* 3).


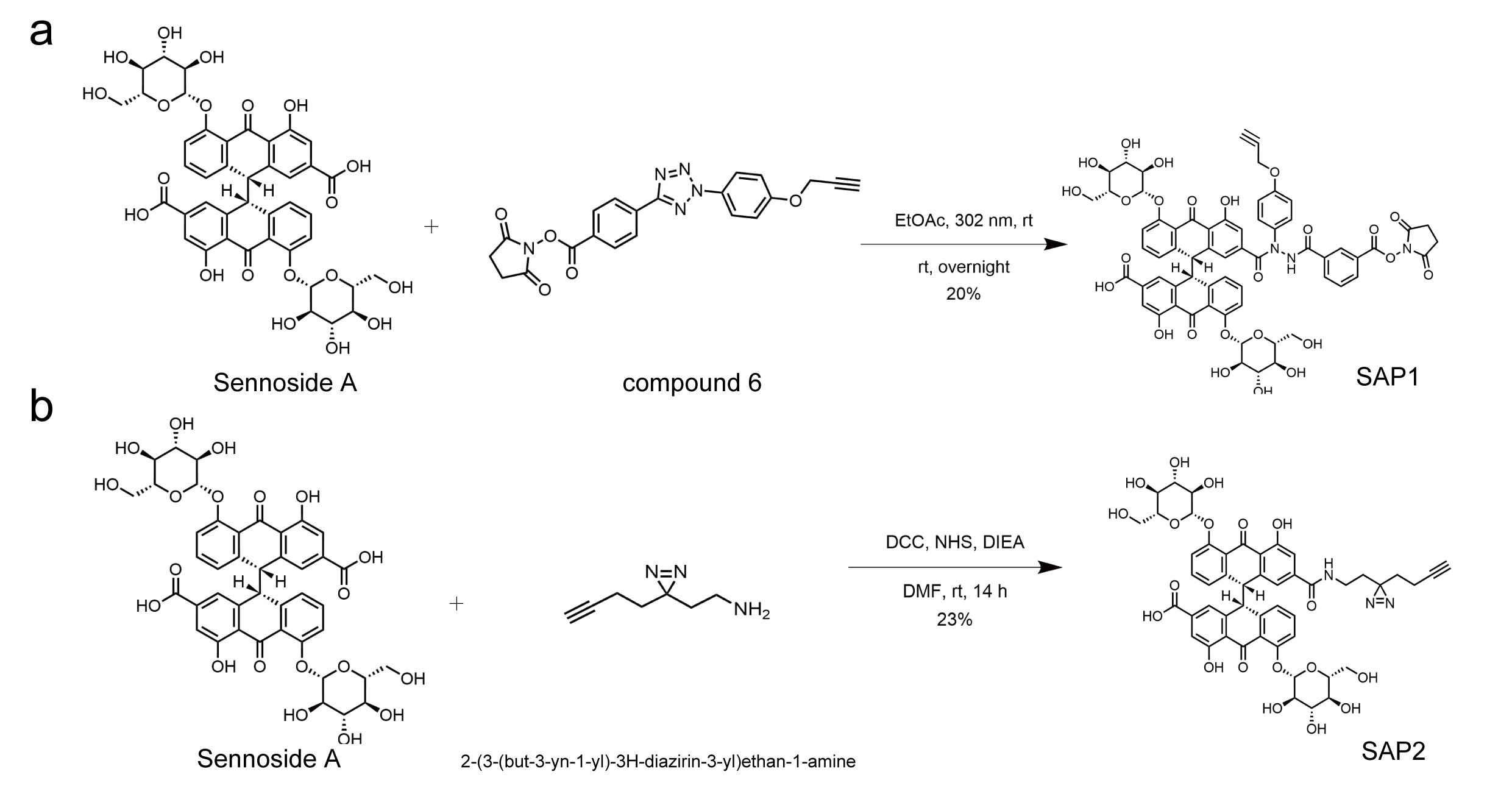


**Figure S4. Synthetic routes for activity-based probes.**

(a) Synthetic route of SAP1. (b) Synthetic route of SAP2. Abbreviations: EtOAc, ethyl ethanoate; rt, room temperature; DCC, *N*,*N*’-dicyclohexylcarbodiimide; NHS, *N*-hydroxysuccinimide; DIEA, *N*,*N*’-diisopropylethylamine; DMF, *N*,*N*-dimethylformamide.


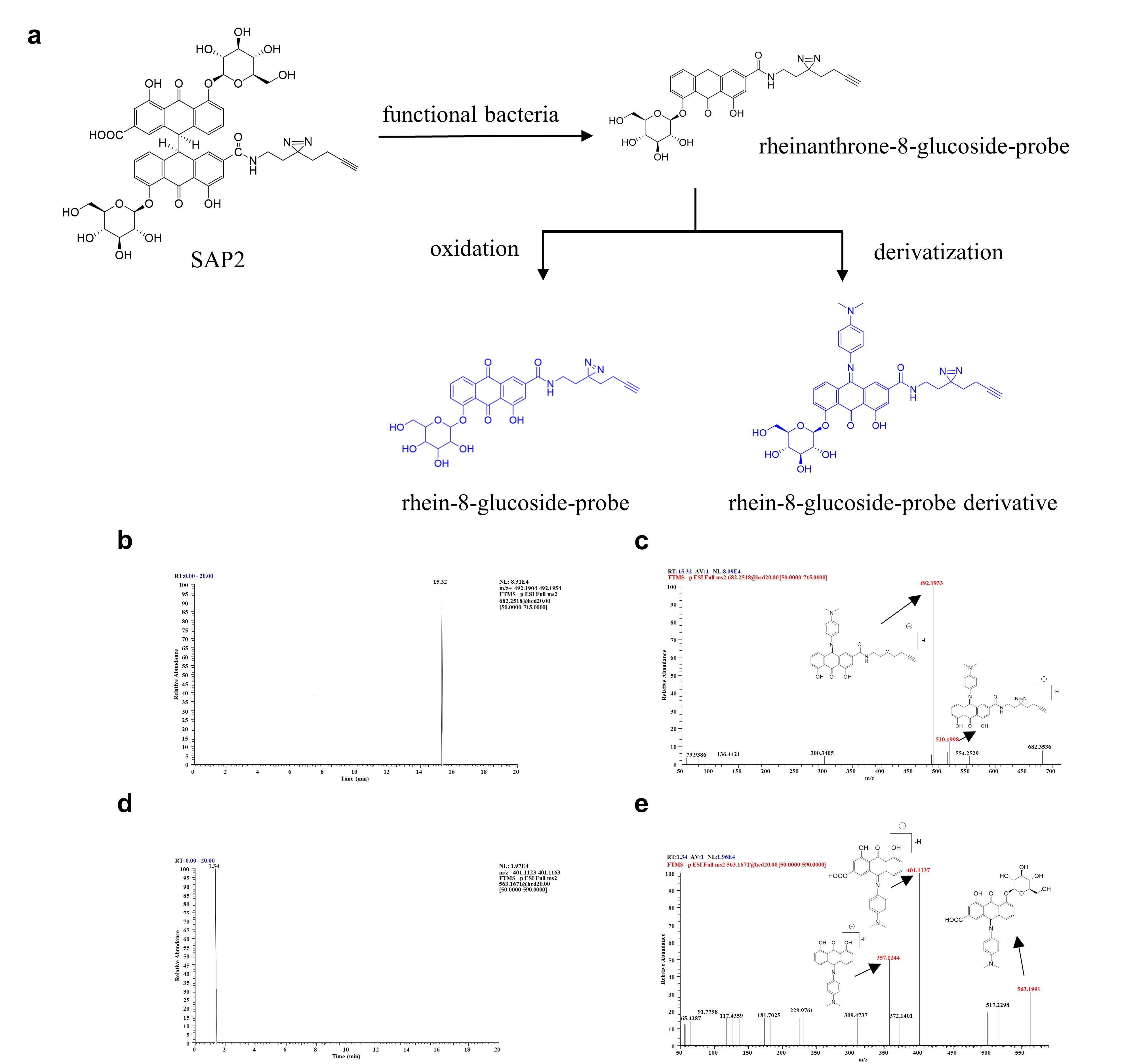


**Figure S5.** **Sennoside A-like bioactivity of SAP2 in functional bacteria.**

(a) Reductive metabolism of SAP2 by *Bifidobacterium pseudocatenulatum*: EIC and MS^2^ spectrum of SAP2 metabolites after 24 h incubation. (b) EIC of rheinanthrone-8-glucoside–probe derivative. (c) MS^2^ spectrum of rheinanthrone-8-glucoside–probe derivative. (d) EIC of rheinanthrone-8-glucoside derivative. (e) MS^2^ spectrum of rheinanthrone-8-glucoside derivative.


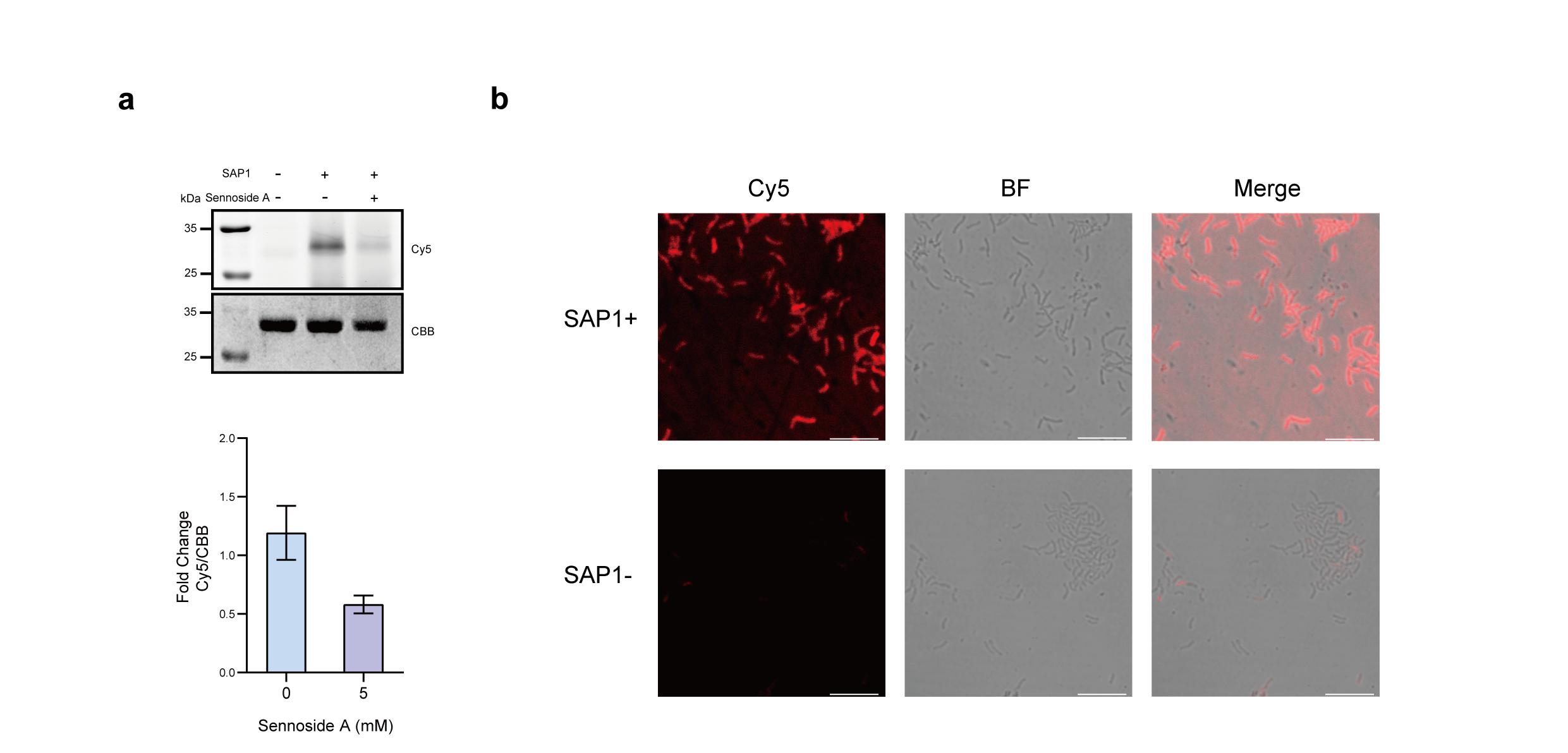


**Figure S6. SAP1 labeling of model enzyme and bacteria.**

(a) Substrate competition assay of SAP1. Purified BpNfrA was preincubated with or without 5 mM of sennoside A, then labeled with SAP1. Data represent mean ± s.d. (*n =* 3). (b) Confocal fluorescence imaging of *Bifidobacterium pseudocatenulatum* with or without SAP1. Bacteria stained with SAP1 are shown in red. Scale bar, 10 μm.


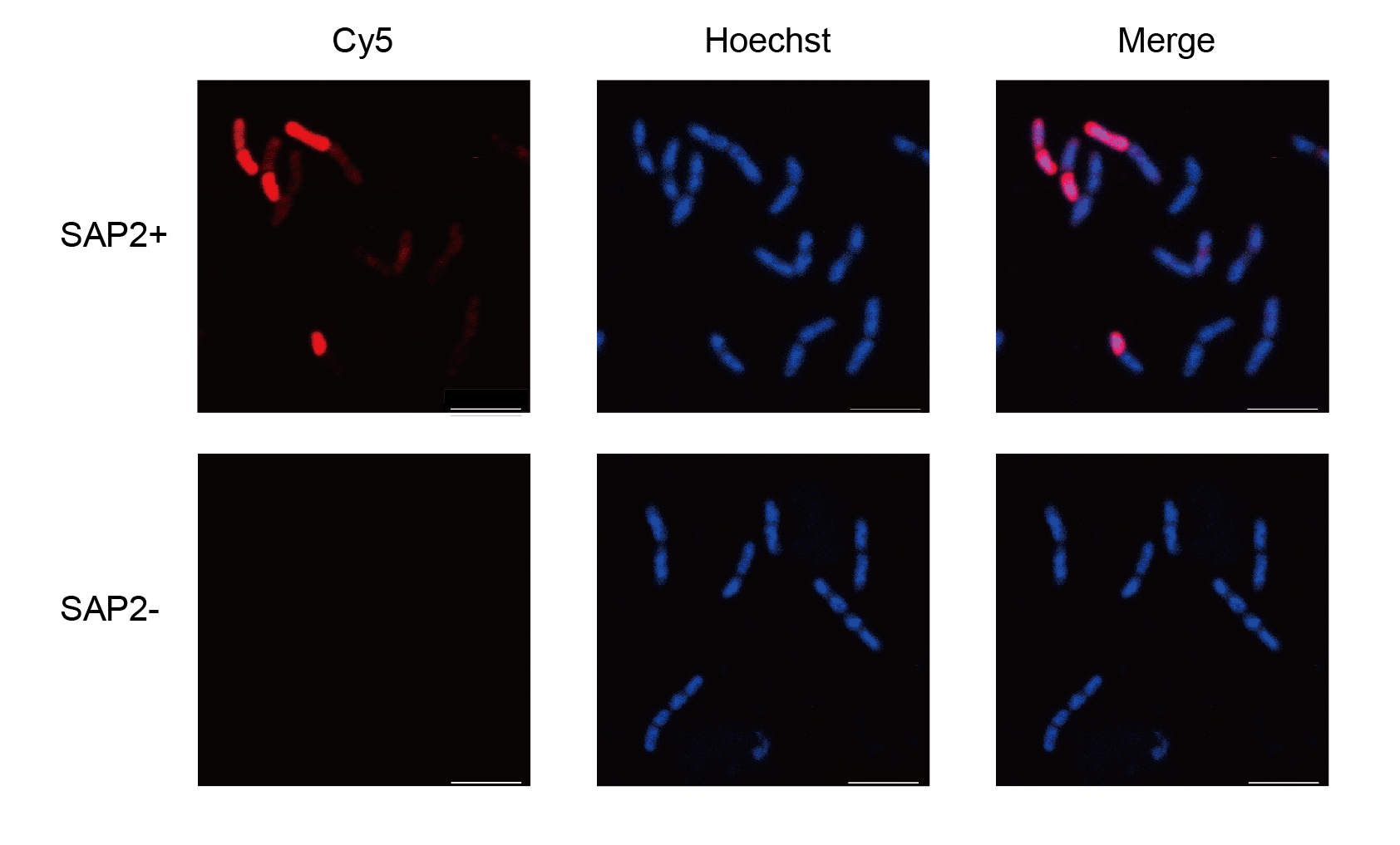


**Figure S7. Confocal fluorescence imaging of *Bifidobacterium pseudocatenulatum* labeled with SAP2.**

Bacteria stained with SAP2 (red) and Hoechst 33342 (blue). Scale bar, 10 μm.


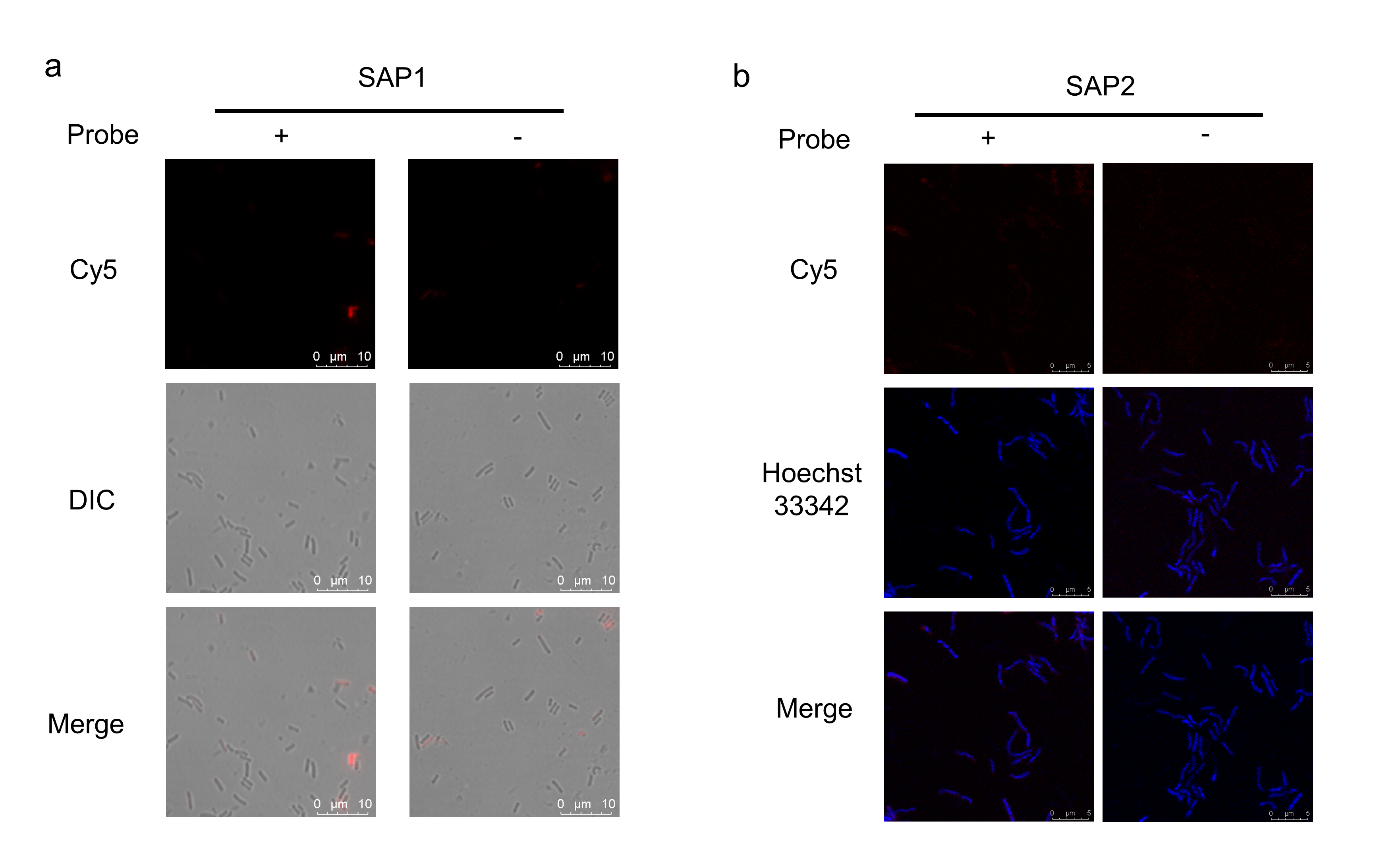


**Figure S8. Confocal fluorescence imaging of *Lactobacillus brevis* labeled with SAP probes.**

(a) SAP1 labeling (red). (b) SAP2 labeling (red) and Hoechst 33342 (blue). Scale bars, 10 μm for (a) and 5 μm for (b).


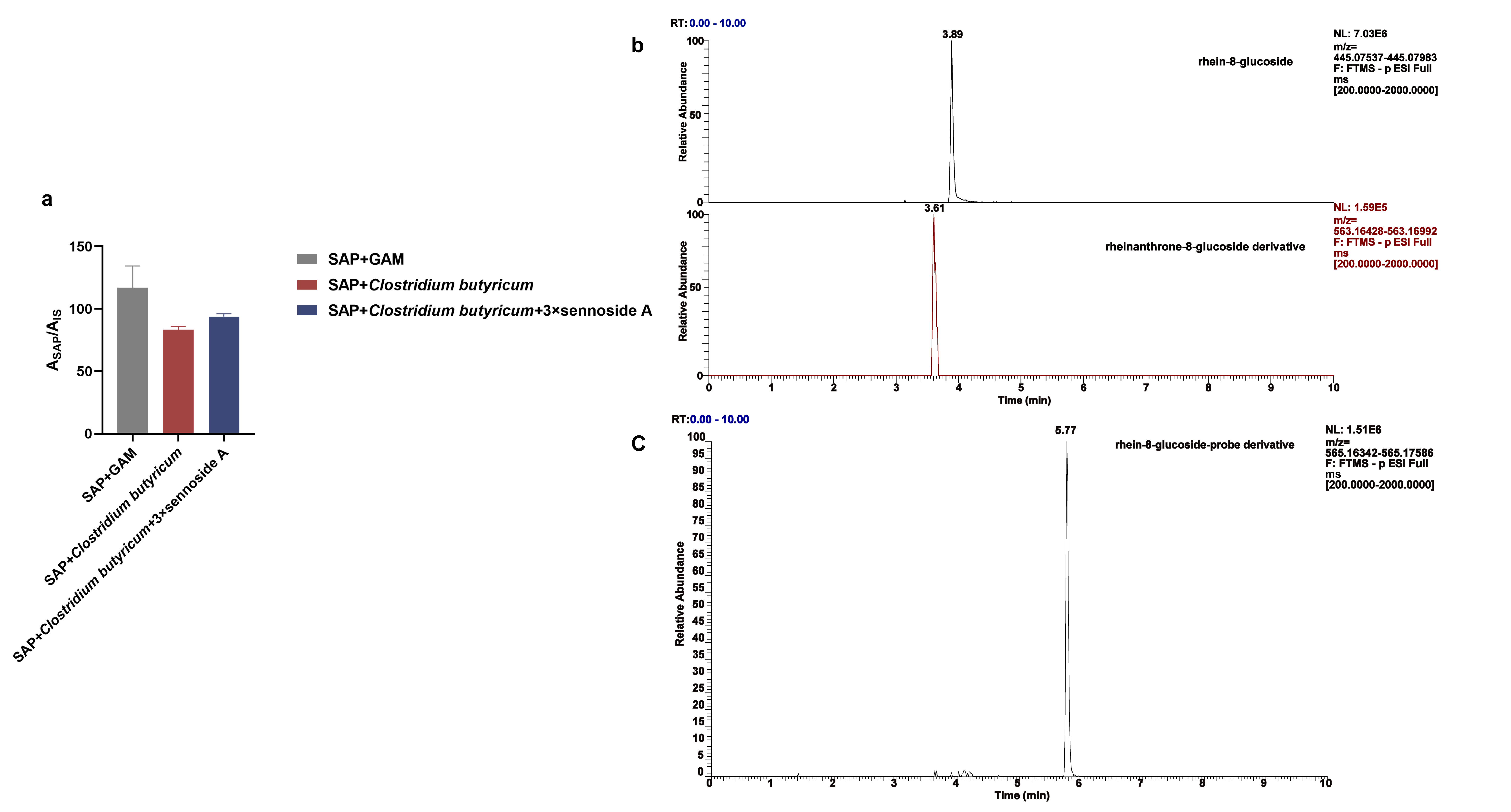


**Figure S9. Substrate competition of SAP2 in *Clostridium butyricum*.**

(a) Peak area ratio of SAP2 after 24 h incubation. (b) EICs of sennoside A–reduced products in the competition group (24 h). (c) EICs of SAP2-reduced products in the probe group (24 h). In (a), data represent mean ± s.d. (*n =* 3 independent experiments).


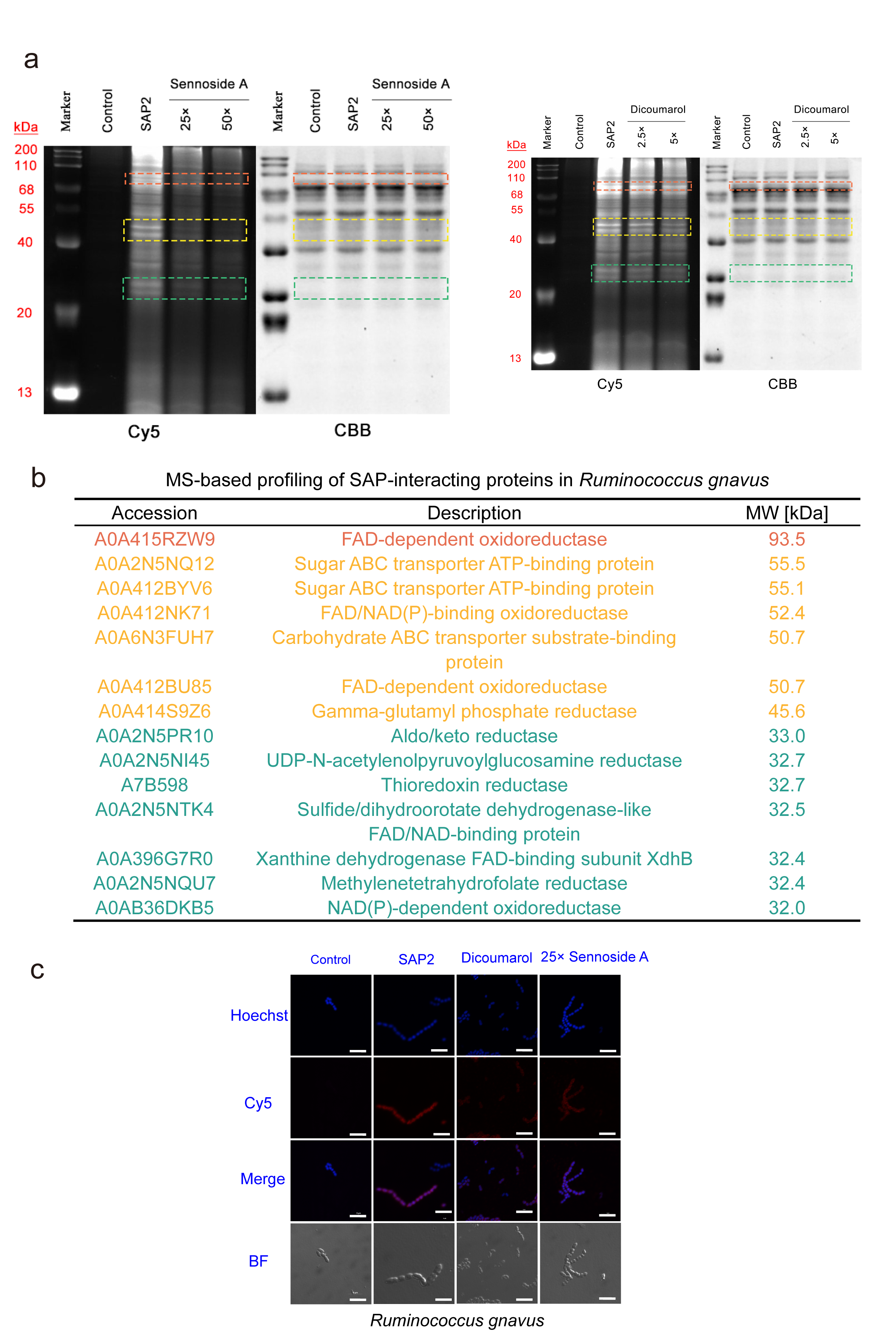
**Figure S10**. **Proteomic profiling of SAP2-interacting proteins in *Ruminococcus gnavus*.**

(a) Gel-based analysis of SAP2-labeled proteins in *Ruminococcus gnavus* with sennoside A competition or dicoumarol inhibition. Bacteria were pre-treated with sennoside A (25×, 50×) or dicoumarol (2.5×, 5×) for 1 h, labeled with 100 μM SAP2 for 1 h, and conjugated with Cy5. CBB-stained gels confirm equal loading. Representative results are shown (*n =* 3 biological replicates). Red, yellow, and green boxes indicate protein bands (90–100, 45–55, and 30–35 kDa) with significant inhibition. (b) MS-based ABPP profiling of SAP2-interacting proteins in *Ruminococcus gnavus*; proteins with significant changes identified from (a) are listed in the accompanying table. (c) Confocal fluorescence imaging of *Ruminococcus gnavus* after SAP2 labeling, including control, SAP2, inhibition, and competition groups. Bacteria were pre-treated with 5× dicoumarol or 25× sennoside A and labeled with 100 μM SAP2 for 1 h, then conjugated with Cy5. Scale bar, 10 μm. Abbreviations: CBB, Coomassie Brilliant Blue; ABPP, activity-based protein profiling.


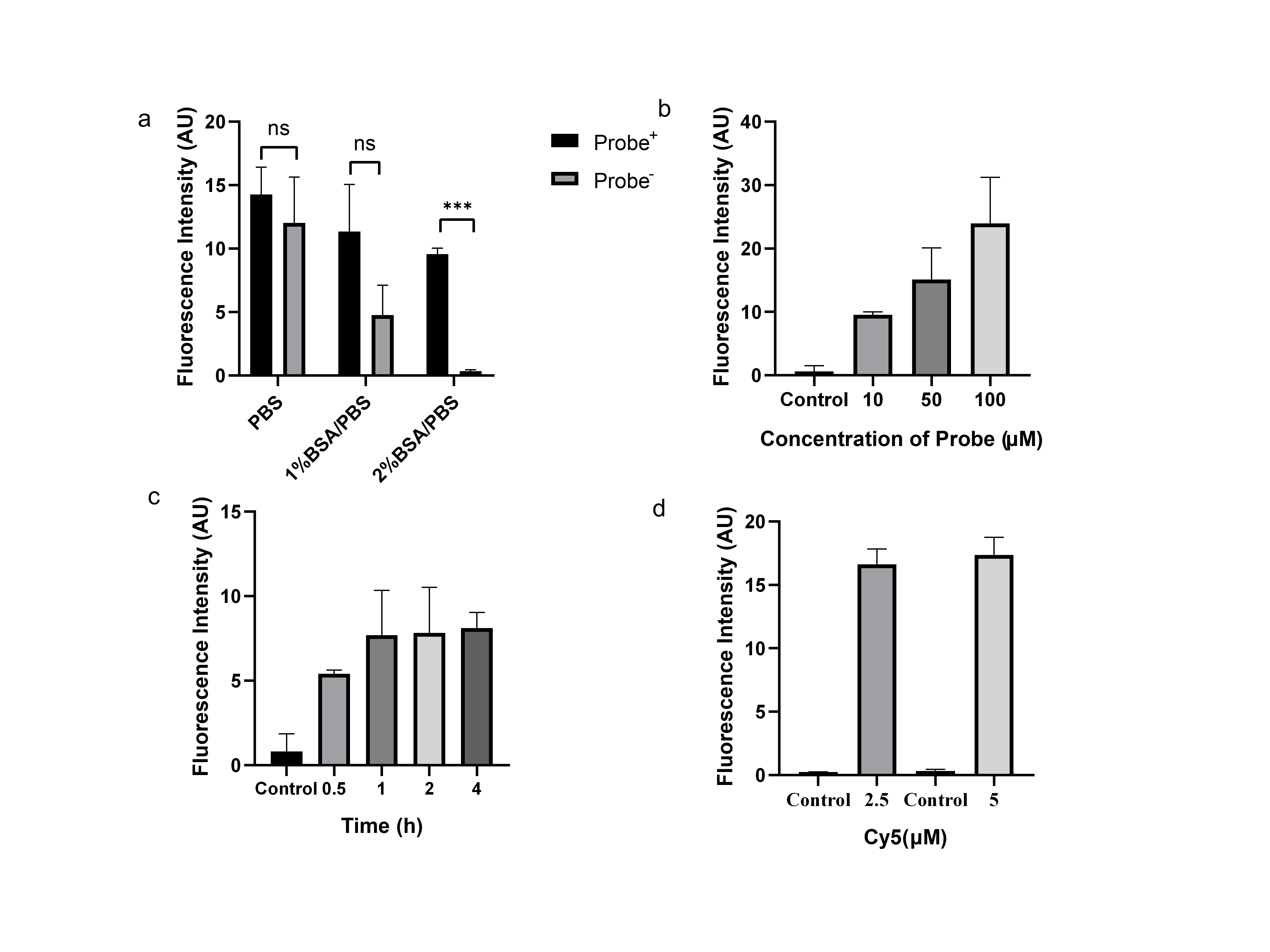


**Figure S11. Optimization of SAP2 labeling conditions in *Bifidobacterium pseudocatenulatum*.**

(a) Effect of washing solvent on fluorescence intensity. (b) Effect of SAP2 concentration (0, 10, 50, 100 μM). (c) Effect of incubation time (0.5, 1, 2, 4 h). (d) Effect of Cy5 concentration (2.5, 5 μM). Data represent mean ± s.d. (*n =* 3). ns, not significant. ****p* < 0.001.


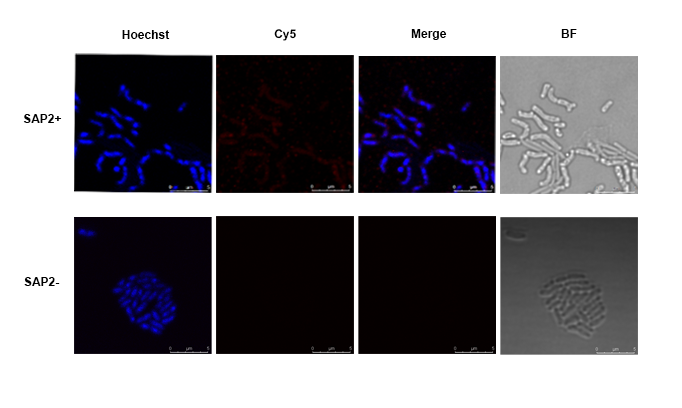


**Figure S12. SAP2 labeling of heat-killed *Bifidobacterium pseudocatenulatum*.**

Bacteria stained with SAP2 (red) and Hoechst 33342 (blue). Scale bar, 10 μm.


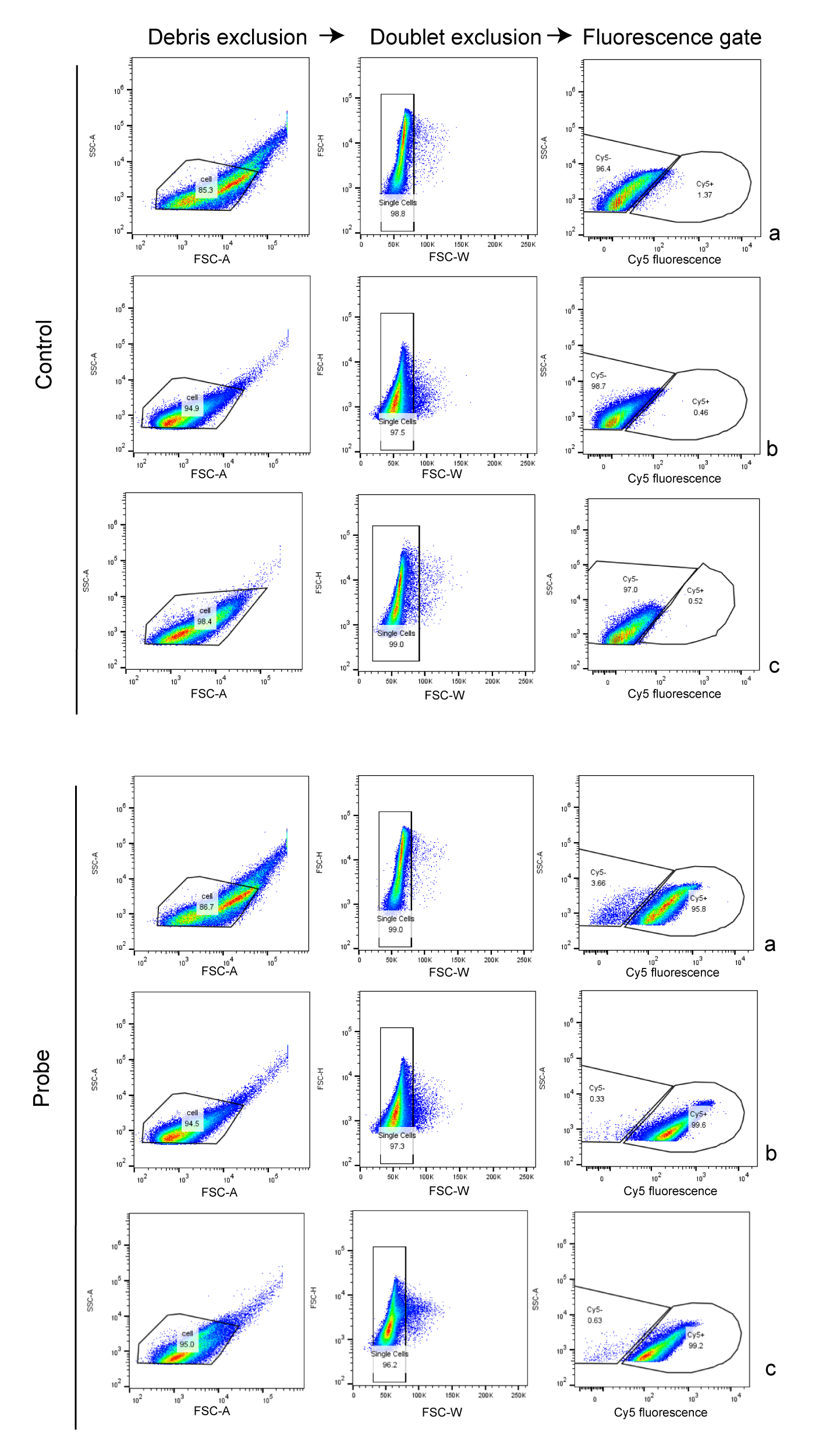
**Figure S13. Flow cytometric analysis of sennoside A–reducing bacteria labeled with SAP2.**

(a) *Clostridium butyricum*; (b) *Bifidobacterium breve*; (c) *Ruminococcus gnavus*. Bacteria were pre-gated to exclude debris and doublets, and analyzed for Cy5 fluorescence signal. Cy5^–^: unlabeled; Cy5^+^: labeled.


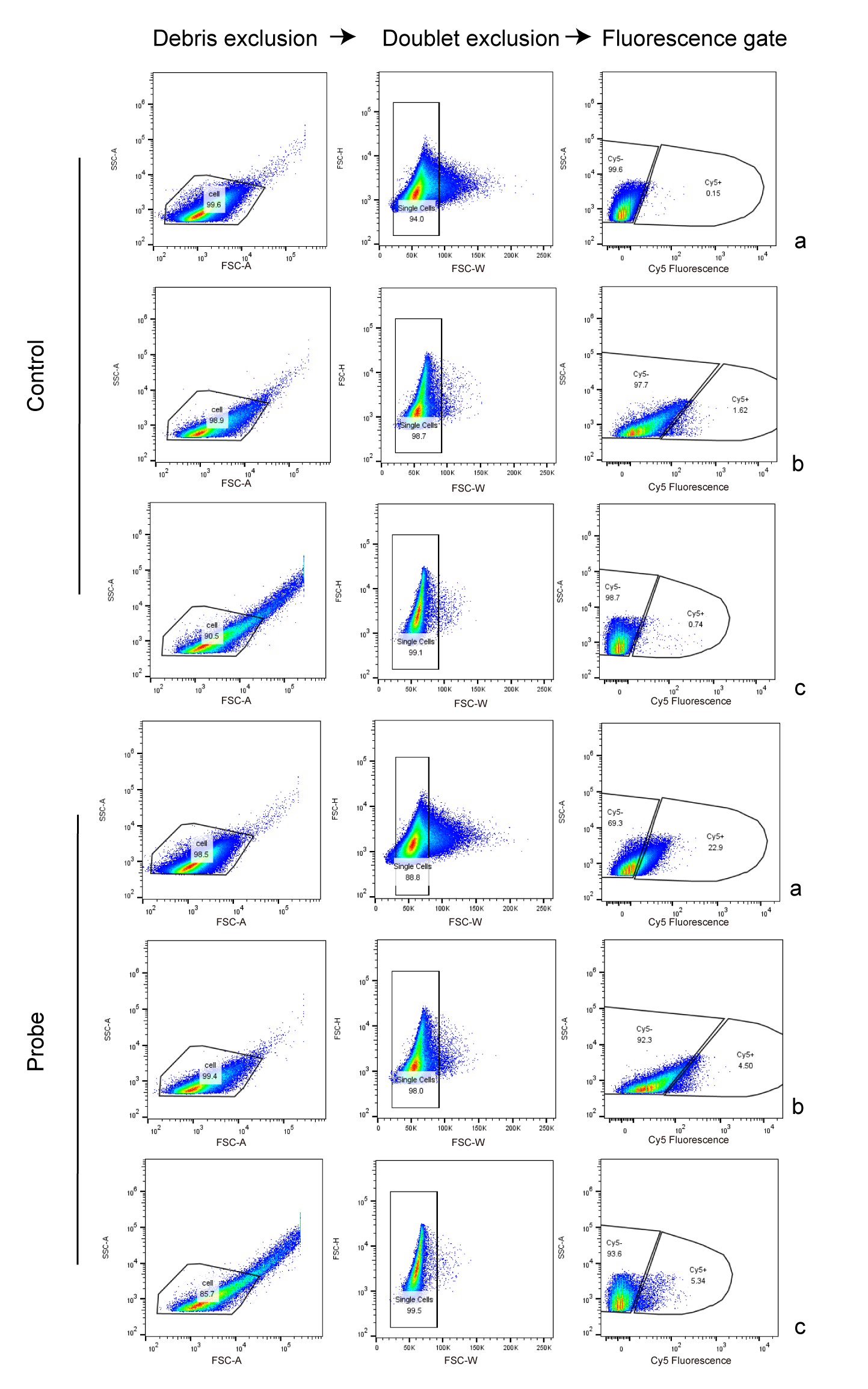
**Figure S14. Flow cytometric analysis of non-sennoside A–reducing bacteria labeled with SAP2.**

(a) *Akkermansia muciniphila*; (b) *Escherichia coli*; (c) *Lactobacillus brevis*. Bacteria were pre-gated to exclude debris and doublets, and analyzed for Cy5 fluorescence signal. Cy5^–^: unlabeled; Cy5^+^: labeled.


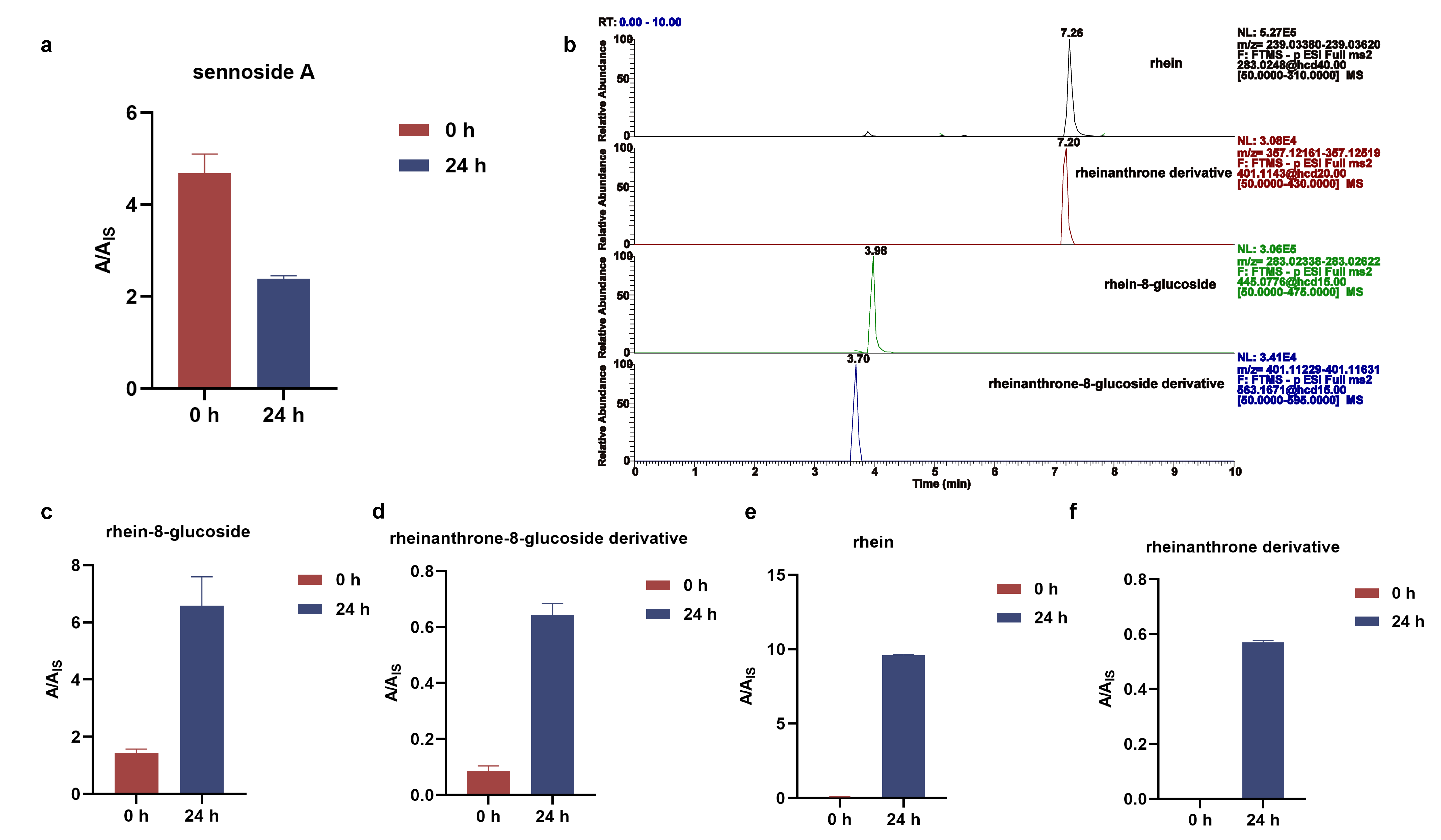


**Figure S15.** **Identification of sennoside A reduction products by *Lactobacillus reuteri*.**

(a) Sennoside A peak area after incubation with *Lactobacillus reuteri*, assessed by LC-MS/MS. (b) EICs of oxidized or derivative products. (c–f) Quantification of rhein-8-glucoside (c), rheinanthrone-8-glucoside derivative (d), rhein (e), and rheinanthrone derivative (f). In (a) and (c–f), data represent mean ± s.d. (*n =* 3).

**
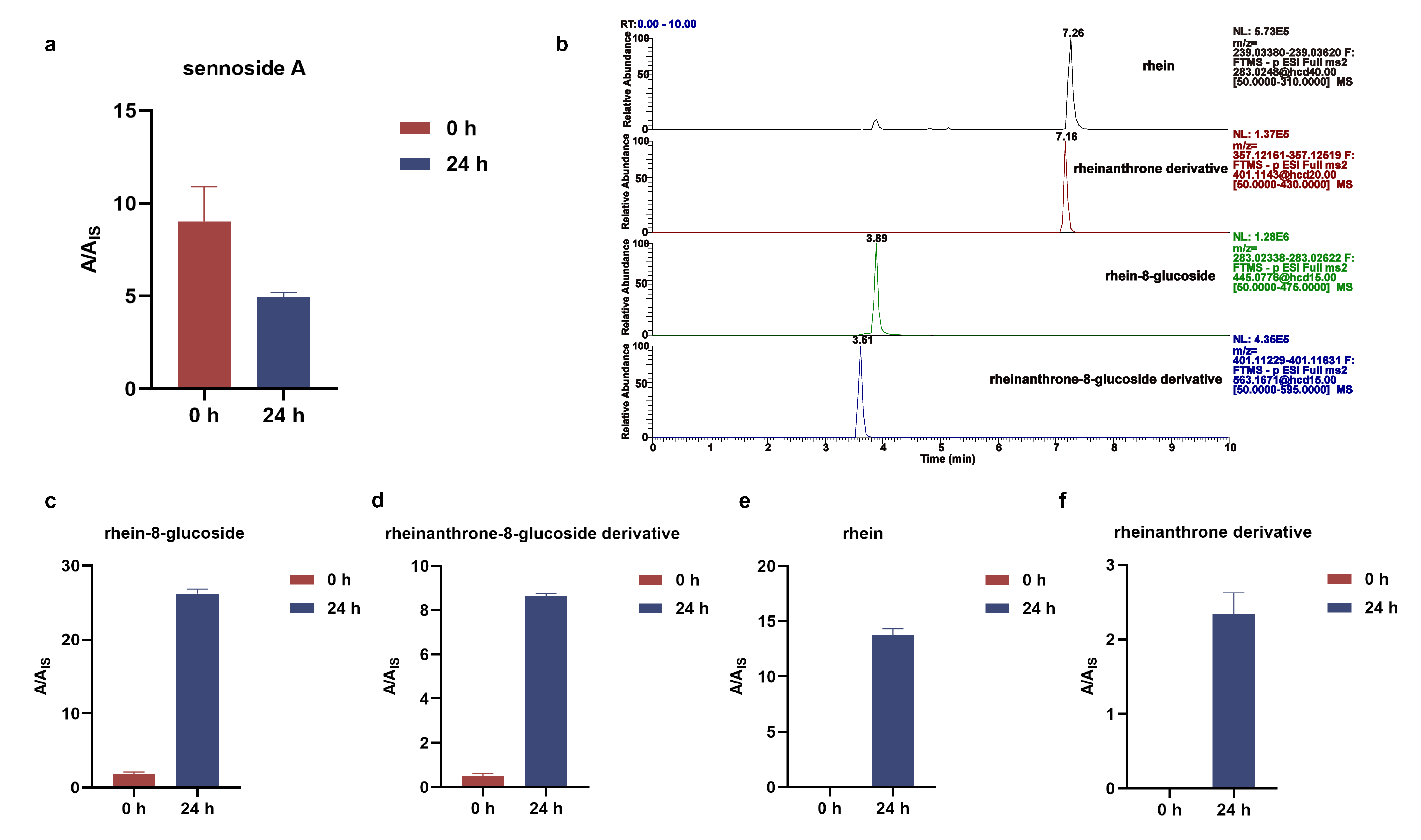
**

**Figure S16. Identification of sennoside A reduction products by** ***Bacteroides ovatus*.**

(a) Sennoside A peak area after incubation with *Bacteroides ovatus*, assessed by LC-MS/MS. (b) EICs of oxidized or derivative products. (c–f) Quantification of rhein-8-glucoside (c), rheinanthrone-8-glucoside derivative (d), rhein (e), and rheinanthrone derivative (f). In (a) and (c–f), data represent mean ± s.d. (*n =* 3).


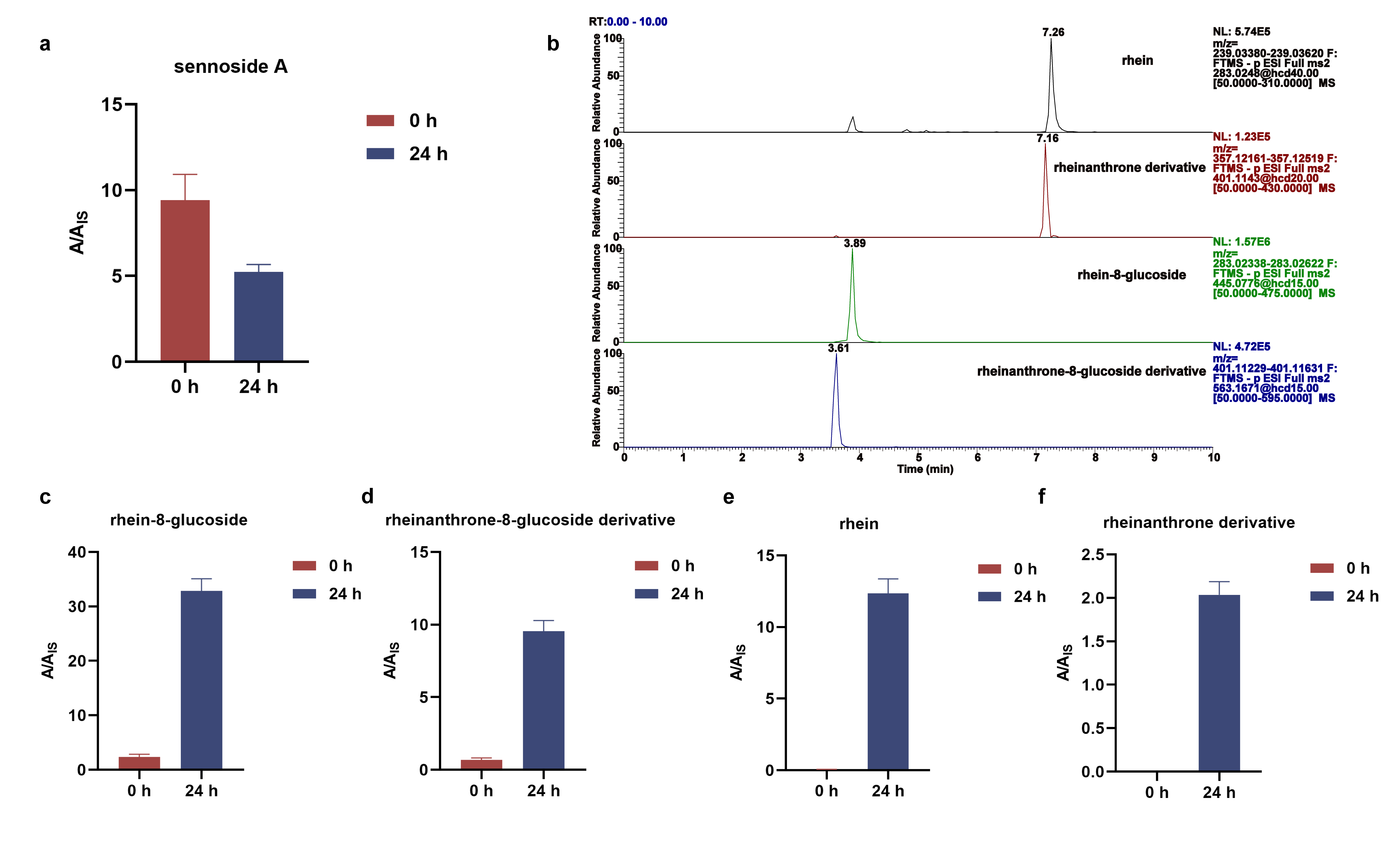


**Figure S17. Identification of sennoside A reduction products by *Bacteroides plebeius*.**

(a) Sennoside A peak area after incubation with *Bacteroides plebeius*, assessed by LC-MS/MS. (b) EICs of oxidized or derivative products. (c–f) Quantification of rhein-8-glucoside (c), rheinanthrone-8-glucoside derivative (d), rhein (e), and rheinanthrone derivative (f). In (a) and (c–f), data represent mean ± s.d. (*n =* 3).


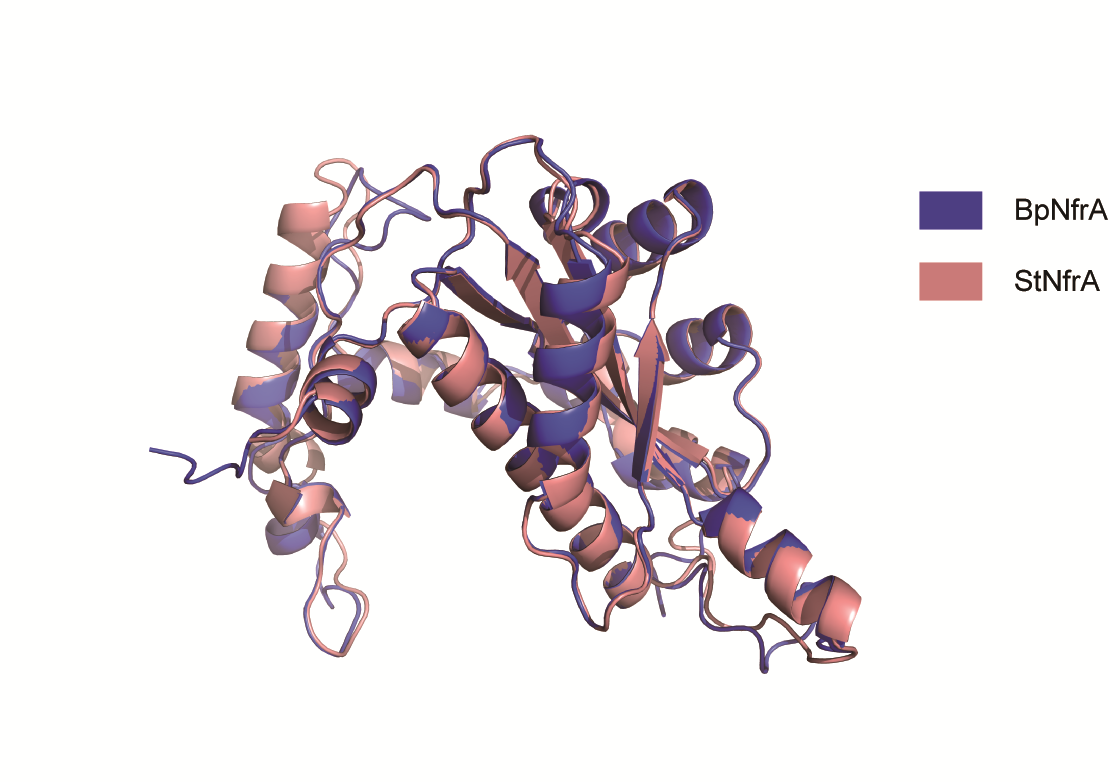


**Figure S18. Structural overlay of BpNfrA and StNfrA.**


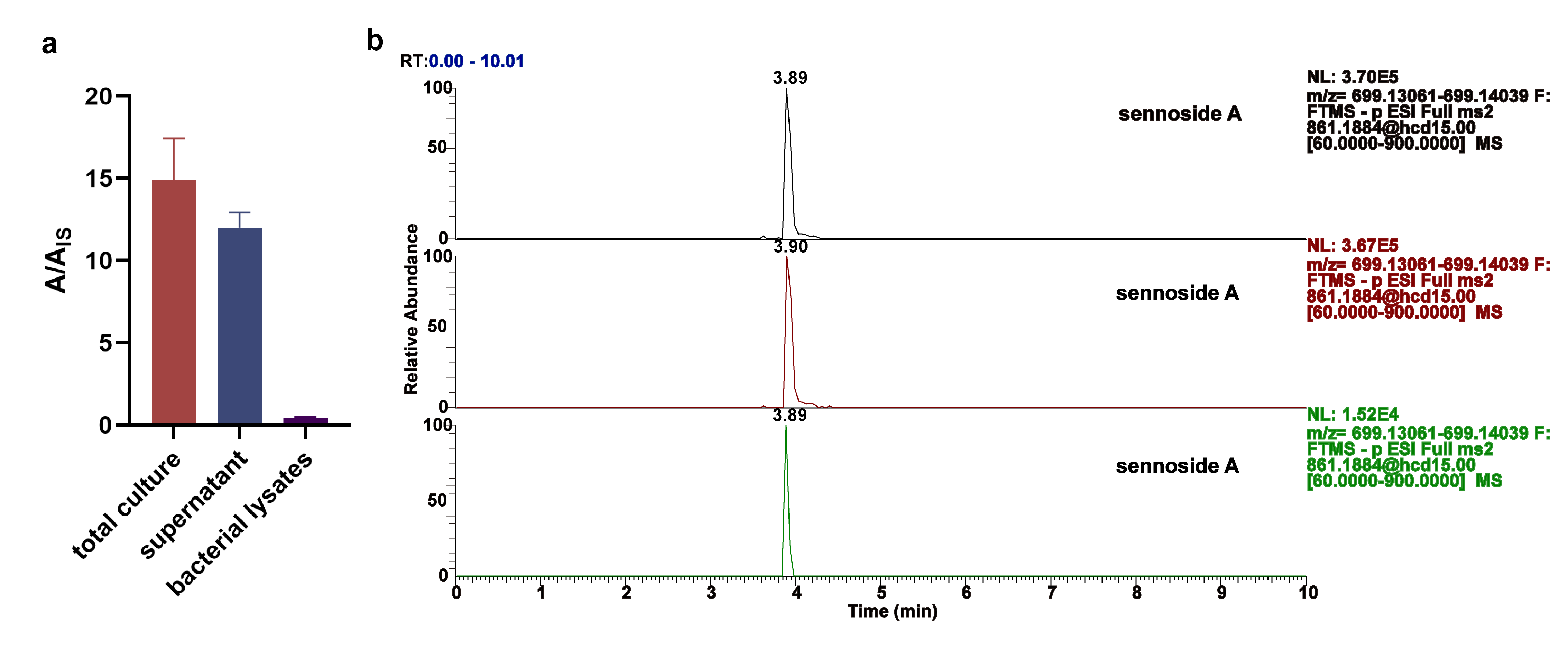


**Figure S19. Quantitative analysis of sennoside A in *Escherichia coli* MG1655 by LC-MS.**

(a) Recovery of sennoside A from total culture (medium plus bacteria), supernatant, and bacterial lysate. (b) EICs of sennoside A from total culture (top), supernatant (middle), and bacterial lysate (bottom). In (a), data represent mean ± s.d. (*n =* 3).

**Preparation of SAP1**

**
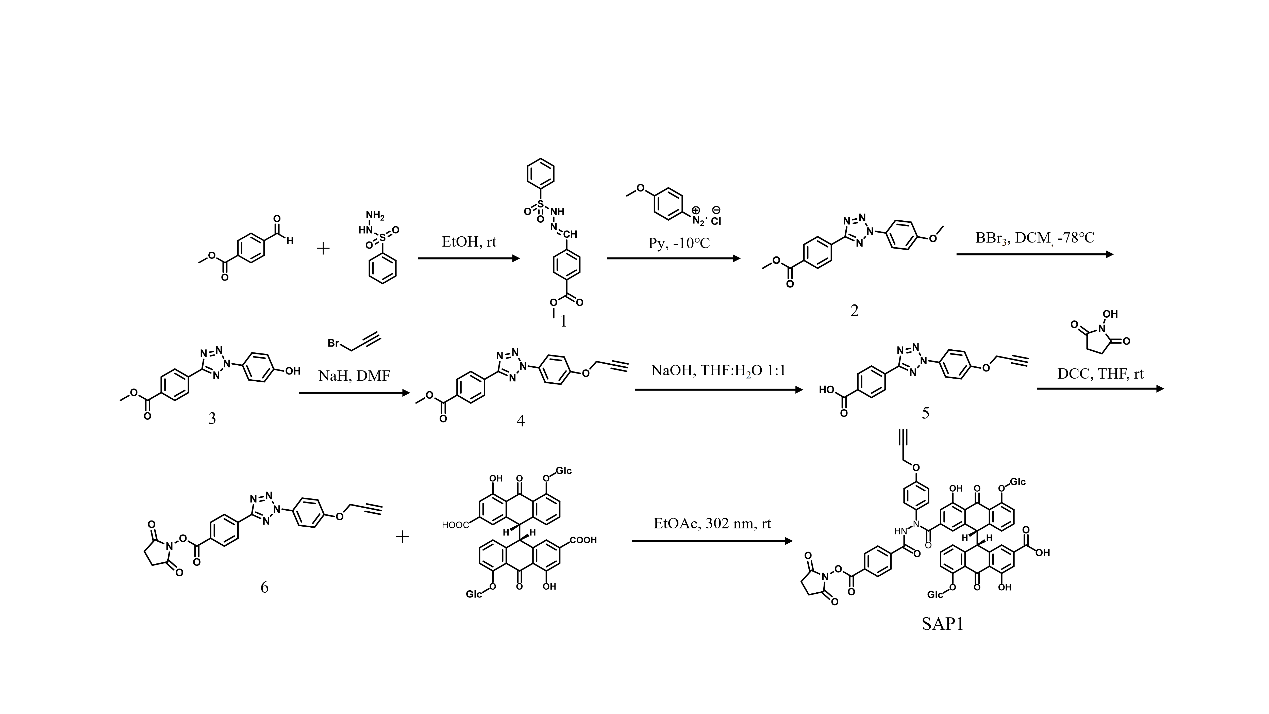
**

Scheme S1. Synthesis of SAP1.


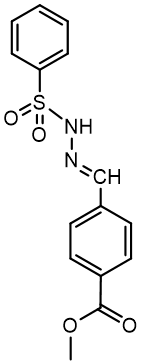


Compound 1^[1]^

To a solution of 2.4601g methyl 4-formylbenzoate (15 mmol) in 113 mL EtOH was added 2.5808 g benzenesulfonohydrazide (15 mmol). The reaction mixture was stirred at room temperature for 5 h. Precipitate was filtered to give 4.2889 g product (yield, 90%). ^1^H NMR (400 MHz, DMSO-D_6_): δ 3.88 (3H, s, OCH_3_), 7.60-7.71 (5H, m, CH), 7.88-7.99 (5H, m, CH), 11.80 (1H, s, NH).


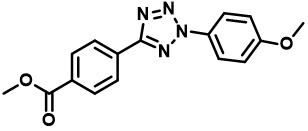


Compound 2^[1]^

To 0.99 g 4-methoxyaniline (8 mmol) and 2.4 mL concentrated HCl in 16 mL 50% ethanol was added 0.5656 g NaNO_2_ in 2 mL water dropwise at 0℃. The obtained 4-methoxybenzenediazonium chloride solution was then slowly added to a solution of 2.5445 g compound 1 (8 mmol) in 48 mL pyridine over a period of 30minutes at -10℃. The reaction was subsequently quenched upon the addition of 80 mL DCM and water. The DCM layer was separated, washed with 160 mL 3M HCl and concentrated. The residue was then purified by flash column chromatography on silica gel using HEX /EtOAc as the eluent to give 1.862 g product (yield, 75%). ^1^H NMR (400 MHz, CDCl_3_)：δ 3.90 (3H, s, OCH_3_), 3.96 (3H, s, CH3), 7.06-7.08 (2H, d, *J* =9.6Hz, CH), 8.10-8.12 (2H, d, *J* = 9.6Hz, CH), 8.18-8.19 (2H, d, *J* =8.8Hz, CH), 8.31-8.33(2H, d, *J* =8.8Hz, CH).


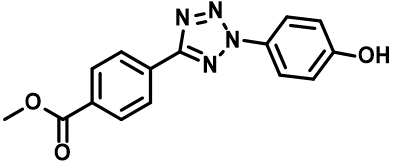


Compound 3^[2]^

Under the N_2_ atmosphere, 776 mg compound 2 (2.5 mmol) was dissolved in 100 mL dried DCM at -78 ℃, and then added BBr_3_ (10 equiv, diluted with dried DCM) slowly. After raising the temperature to RT gradually, the solution was stirred overnight. The reaction mixture was quenched with saturated sodium bicarbonate, extracted with EtOAc and dried over sodium sulfate. Then the organic solvent was removed under vacuum, and the resulting residue was purified by flash column chromatography on silica gel using HEX/EtOAc as the eluent to give 296 mg product (yield, 40%) ^1^H NMR (400 MHz, DMSO-D_6_): δ 10, 26 (H, s, OH); 8.27-8.29 (2H, d, *J* = 8.36, CH), 8.15-8.17 (2H, d, *J* =8.39, CH); 7.96-7.98 (2H, d, *J* =8.93, CH), 7.02-7.04 (2H, d, *J* = 8.96, CH), 3.91 (3H, s, CH_3_)


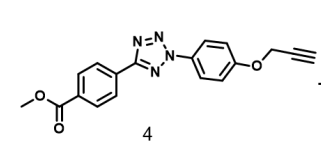


Compound 4^[2]^

Under a N_2_ atmosphere, to a 6 mL dry DMF solution of 180 Compound 3 (0.61 mmol) at 0℃ were added 18 mg NaH (0.73 mmol) and 81 µL propargyl bromide (0.73 mmol) slowly. After raising the reaction temperature to RT gradually, the solution was stirred overnight. The reaction mixture was quenched with saturated ammonium chloride, extracted with EtOAc and dried over sodium sulfate. Then the organic solvent was removed under vacuum, and the crude product goes directly to the next step of the reaction.


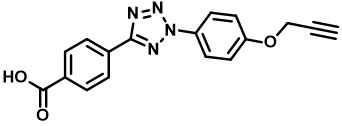


Compound 5^[1]^

To a solution of 31.17 mg crude Compound 4 in 1.2 mL THF was added 32 mg NaOH (0.8 mmol) in 1.2 mL H_2_O. The reaction mixture was stirred vigorously at room temperature for overnight. The reaction was quenched by partitioning the reaction mixture between 3 mL EA and 3mL water. The aqueous layer was the separated washed with 3 mL EA, and acidified with 2N HCl to pH =1. Precipitate was collected to give 25mg product (yield, 97%). ^1^H NMR (400 MHz, DMSO-D_6_): 8.26-8.28 (2H, d, *J* = 8.40, CH), 8.14-8.17 (2H, d, *J* =8 40, CH); 8.11-8.13 (2H, d, *J* = 9.11, CH), 7.28-7.30 (2H, d, *J* =9.13, CH), 4.95-4.96 (2H, d, *J* = 2.34, CH2), 3.65-3.67 (1H, t, *J* = 2.33, CH).


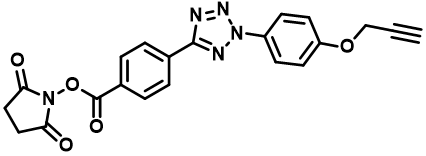


Compound 6^[1]^

To 11.52 mg Compound 5 (0.036 mmol) in 2 mL dry THF was added 8.17 mg DCC (0.0392 mmol) and 6.45 mg *N*-hydroxysuccinimide (0.056 mmol). The reaction mixture was stirred at room temperature for 24 h. Precipitate was removed by filtration. The filtrate was subsequently diluted with 6mL water, and the precipitate was purified by flash column chromatography on silica gel using HEX/EtOAc as the eluent to give 12.51 mg product (yield, 83%). ^1^H NMR (400 MHz,DMSO-D_6_): 8.43-8.45 (2H, d, *J* = 8.51, CH), 8.32-8.34 (2H, d, *J* = 8.51,CH), 8.12-8.15 (2H, d, *J* =9.09, CH), 7.29-7.31 (2H, d, *J* = 9.13, CH), 4.95-4.96 (2H,d,*J* = 2.35,CH_2_), 3.65-3.67 (1H, t, *J* =2.33, CH), 2.93 (4H, s, CH2); ^13^C NMR (400 MHz, DMSO-D_6_): δ 170.73, 163.58, 161.81, 158.98, 133.14, 131.59, 130.51, 127.98, 126.64, 122.36, 116.63, 79.30, 79.18, 56.45, 26.06.


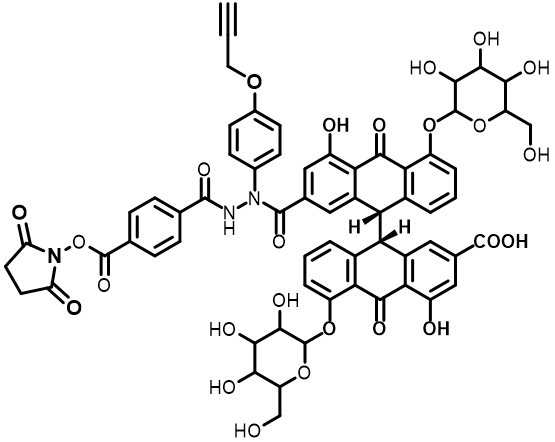


SAP1^[1]^

32.24 mg SA (0.037 mmol) and 7.31 mg Compound 6 (0.017 mmol) were mixed in DMSO:EtOAc=1:1. The reaction mixture was then irradiated by UV at 302 nm for 3 h .The reaction mixture was subsequently purified by flash column chromatography on C18 to give 8.33 mg SAP1 (yield, 30%), ^1^H NMR (700 MHz, DMSO-D_6_): 12.74 (1H, s, OH), 11.47 (1H, s, NH), 8.04-8.20 (4H, m, CH), 7.76 (H, s, CH), 7.25-7.51 (7H, m, CH), 6.55-7.12 (6H, m, CH), 6.55-7.12 (6H, m, CH), 5.30-5.31 (2H, d, *J* = 3.52, CH), 5.02-5.04 (2H, m, OH), 4.78-4.84 (4H, m, OH/CH), 4.62-4.67 (4H, m, OH), 4.25 (1H, t, *J* = 7, 05, OH), 3.98 (2H, s, OH), 3.78-3.82 (2H, m, CH), 3.64-3.67 (6H, m, CH/CH2), 3.57-3.59(2H, m, CH2), 3.48 (H, s, CH), 2.59 (4H, s, CH_2_); ^13^C NMR (700 MHz, DMSO-D_6_)：δ 186.22, 183. 69, 172.79, 160.30, 157.63, 129.45, 127.50, 121.28, 115.06, 110.56, 97.17, 85.39,.78.61, 69.92, 68.30, 65.71, 63.19, 60.47, 25.24.

**MS spectra and MS/MS spectra of SAP1**

**Compound 1**


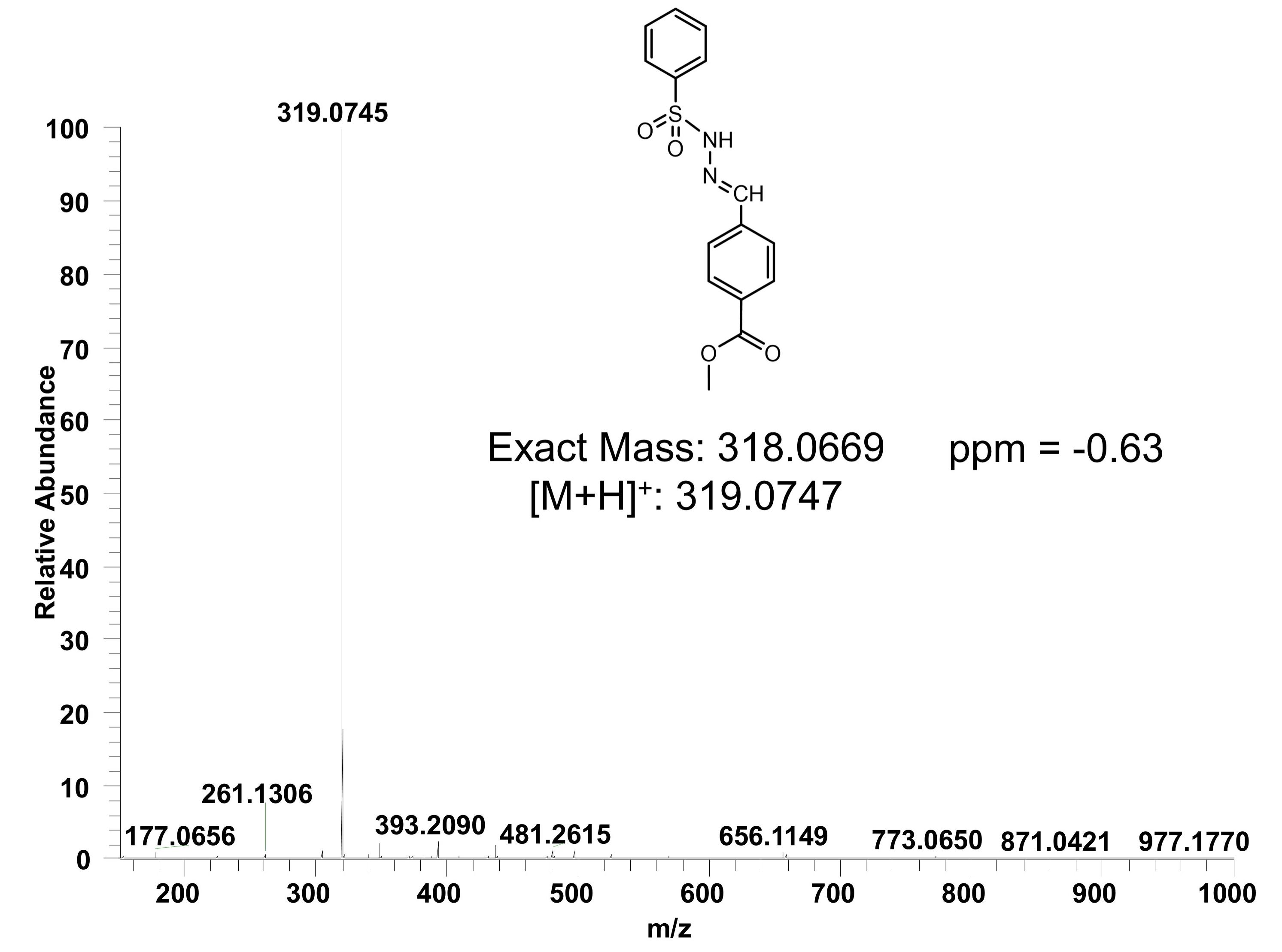

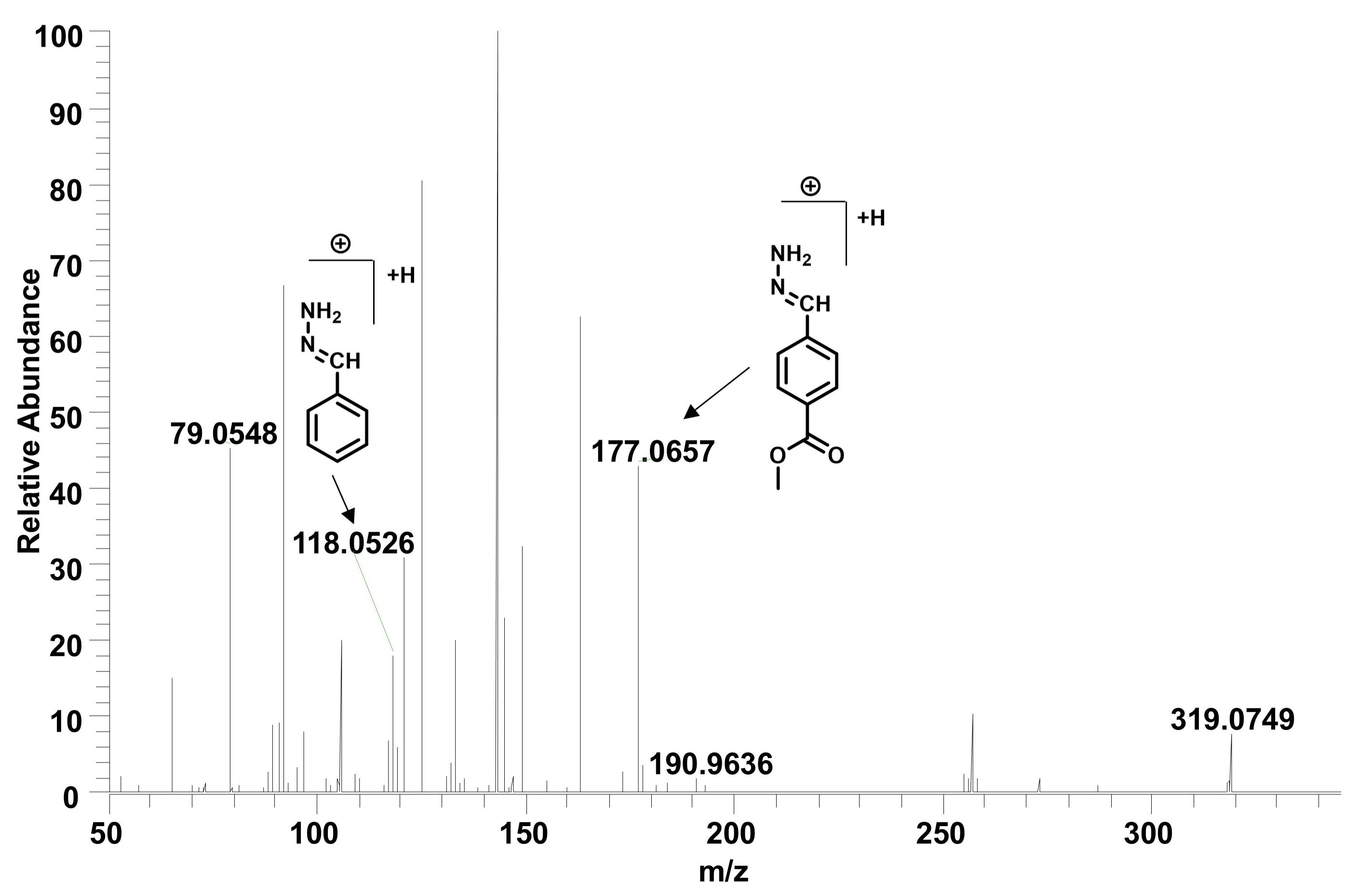


**Compound 2**


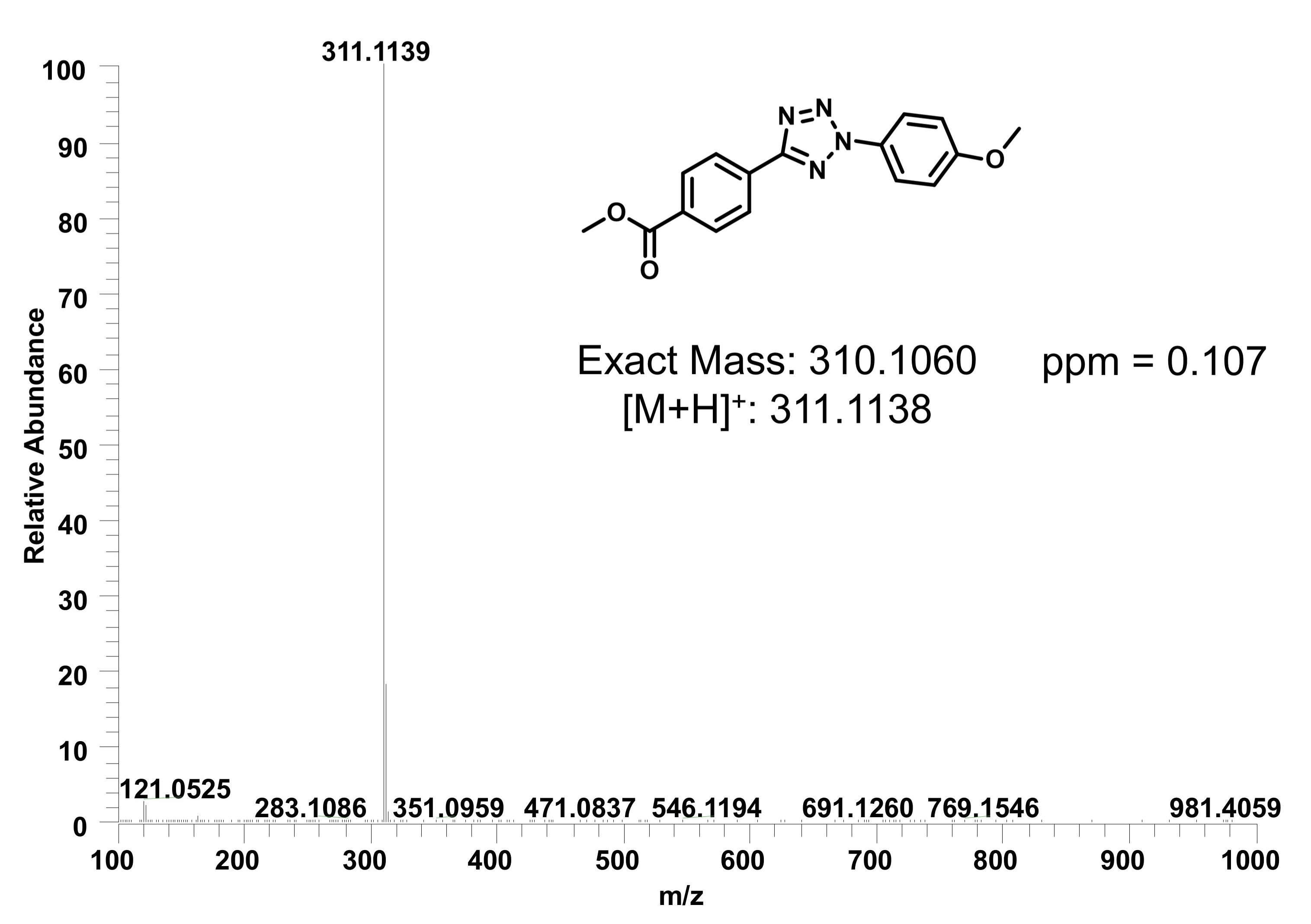

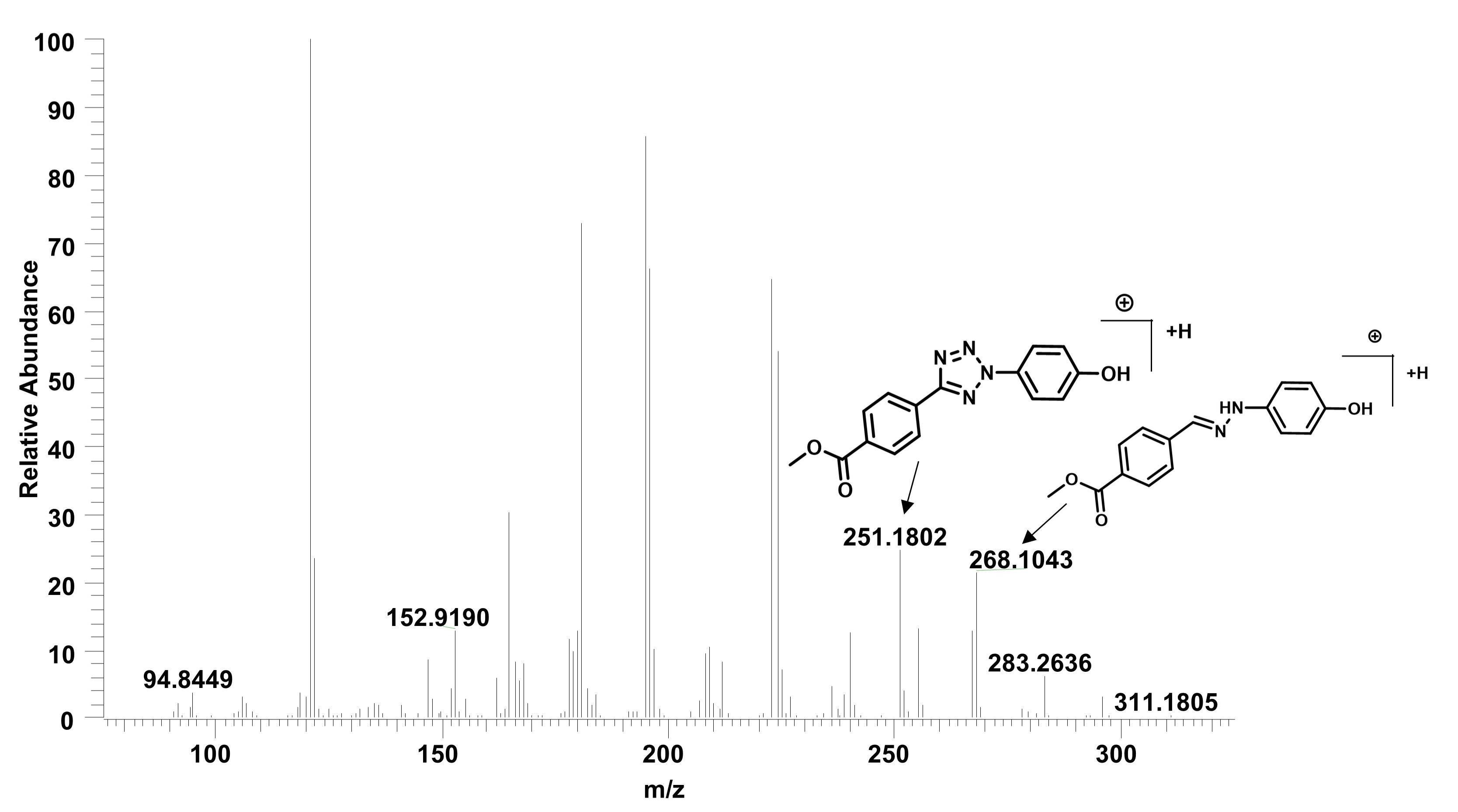


**Compound 3**


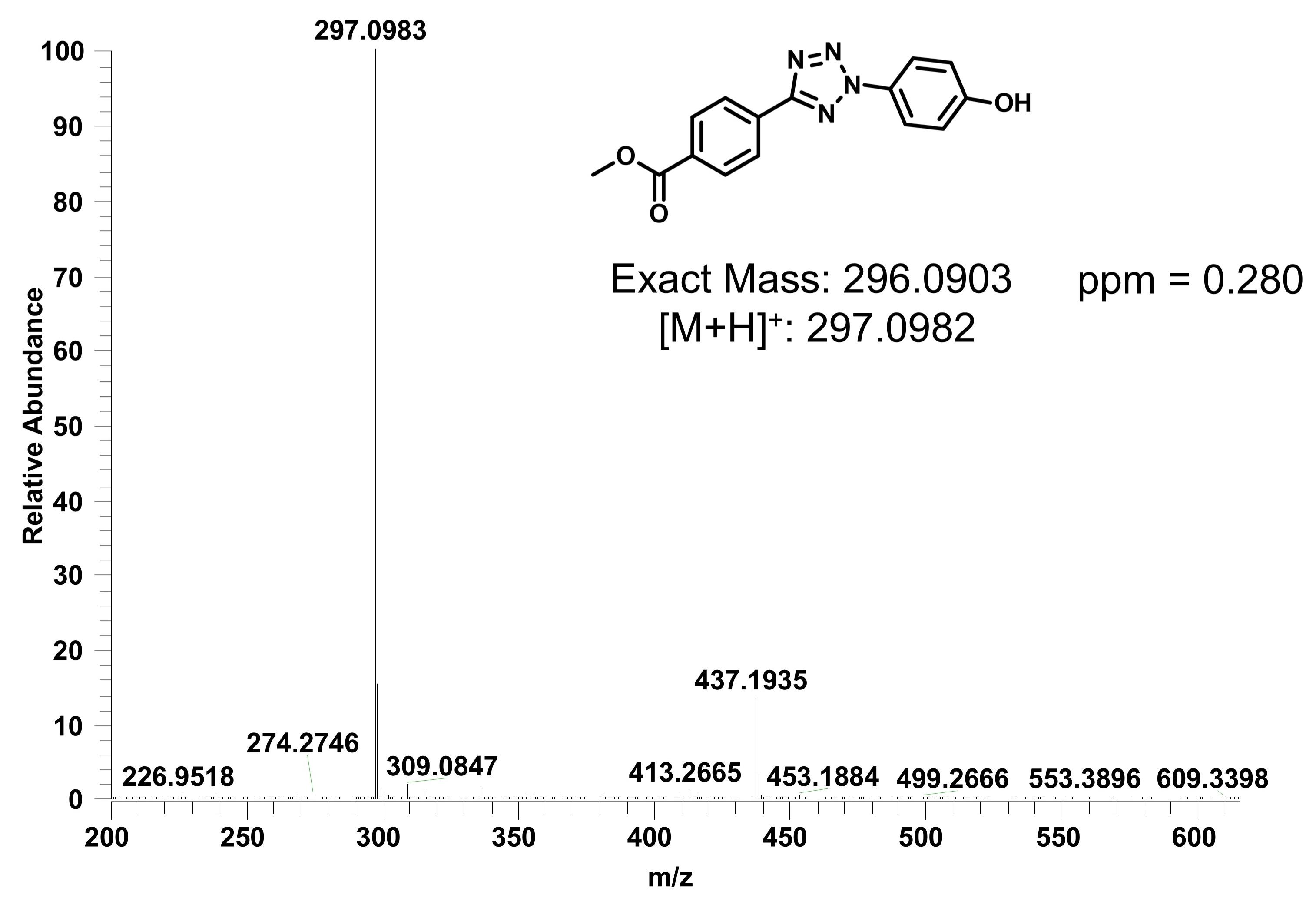


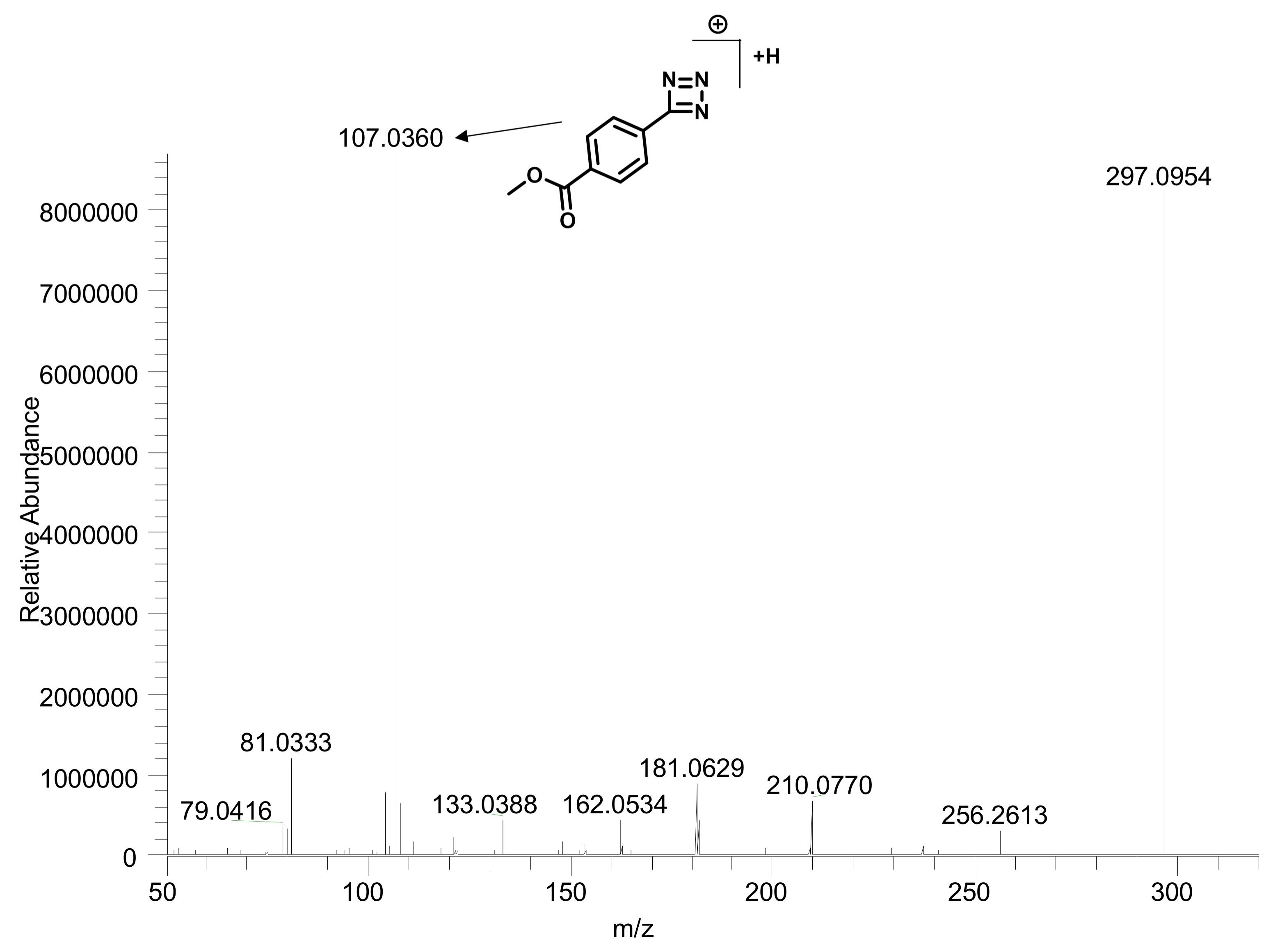


**Compound 4**


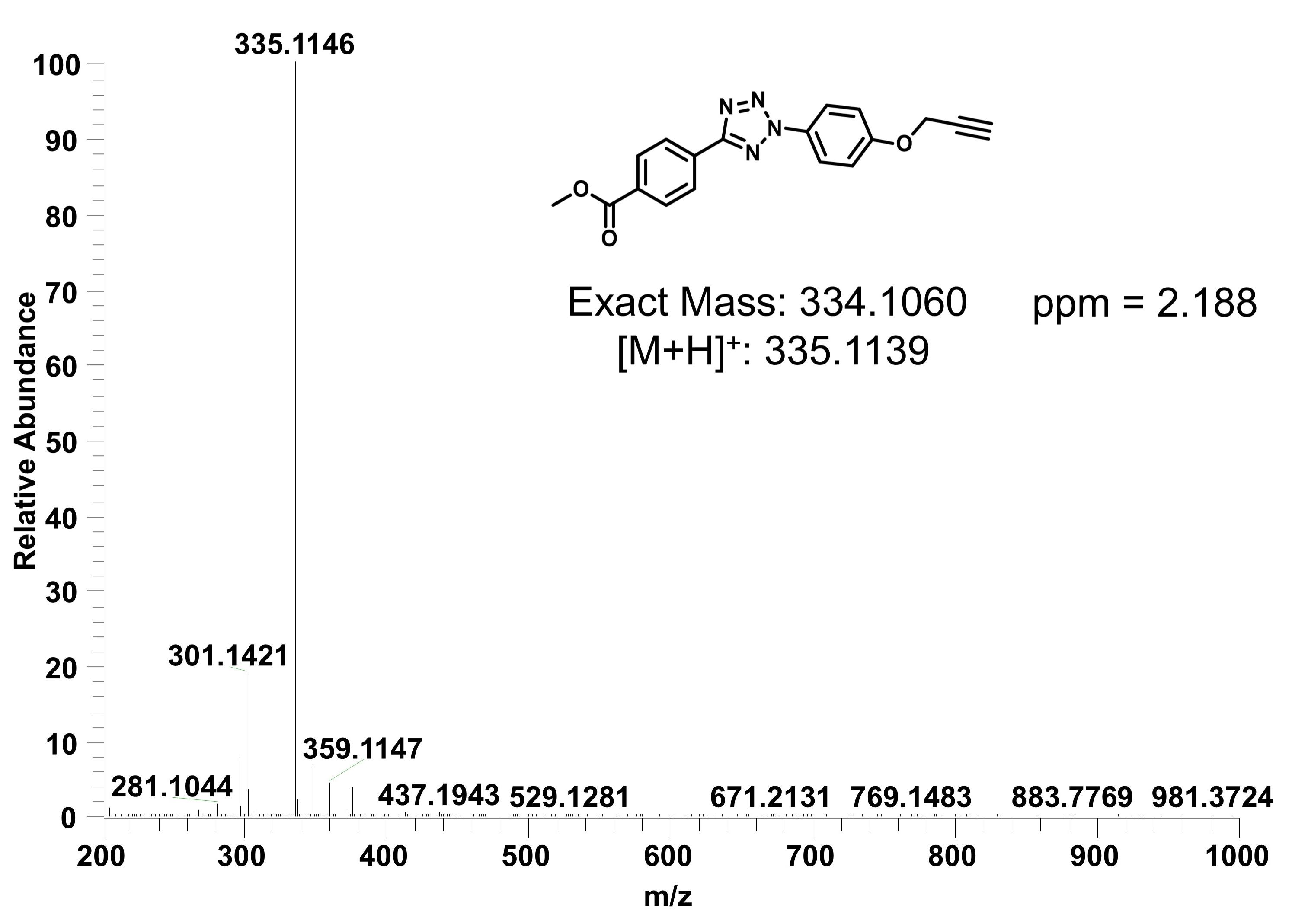

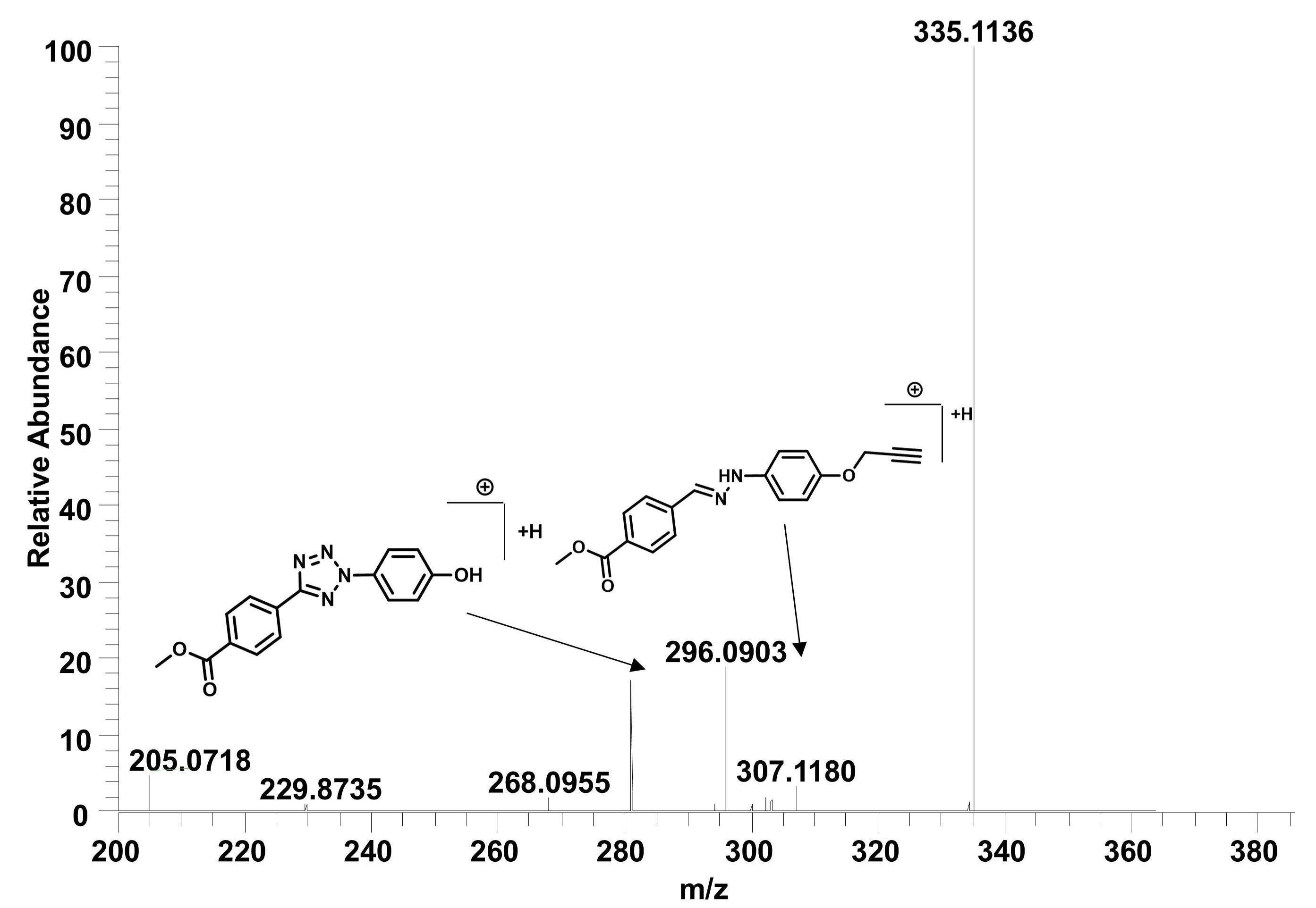


**Compound 5**


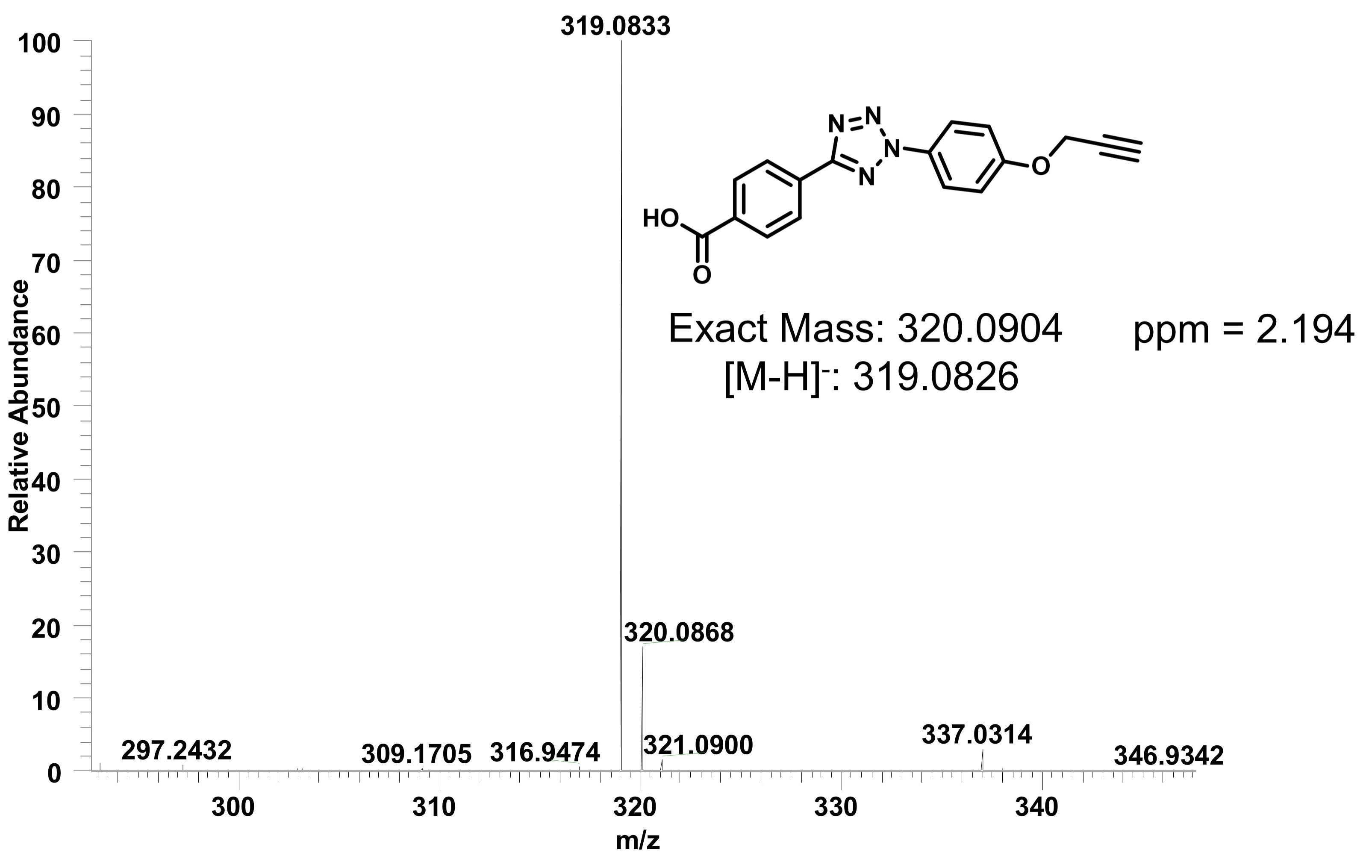


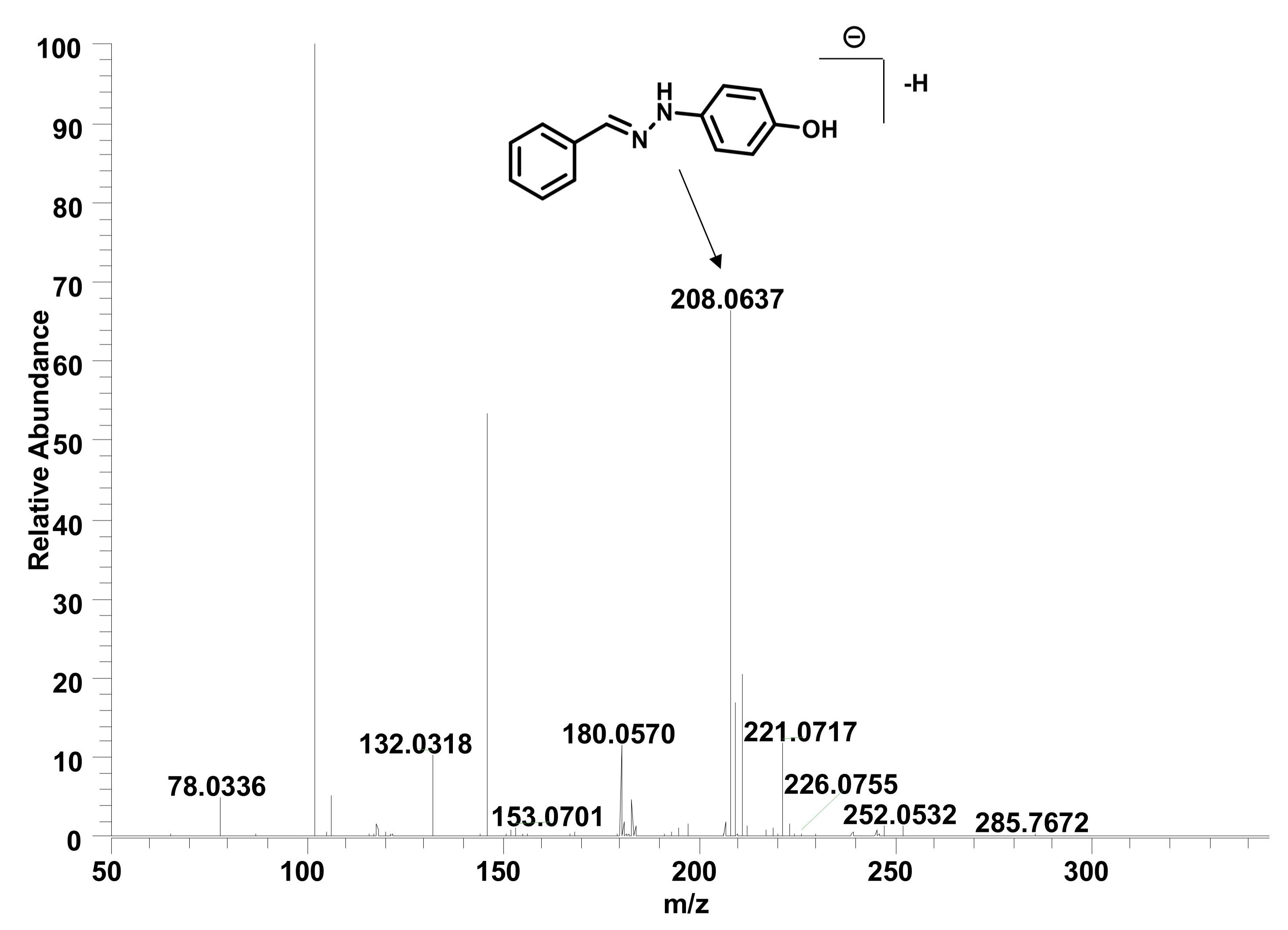


**Compound 6**


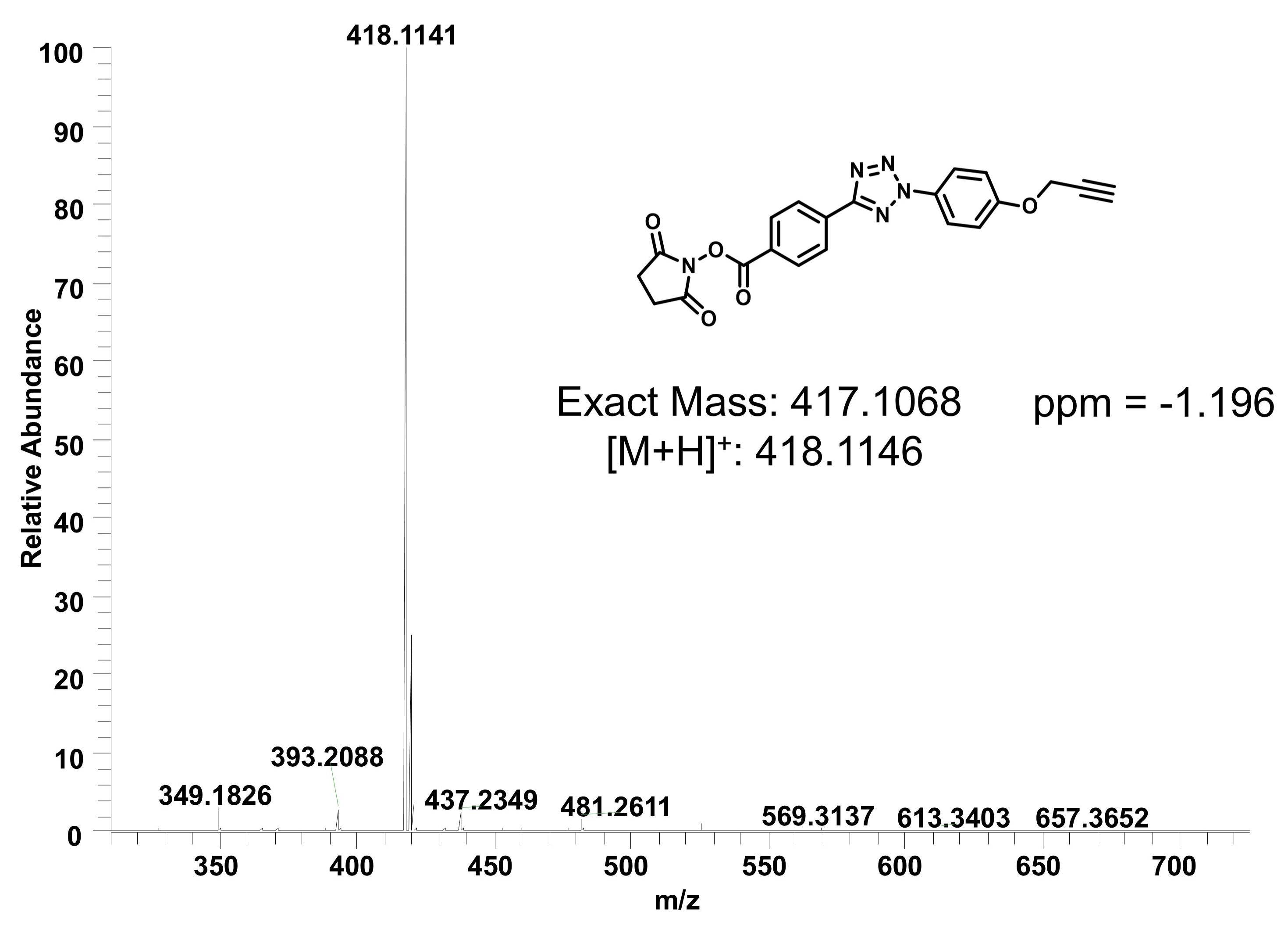

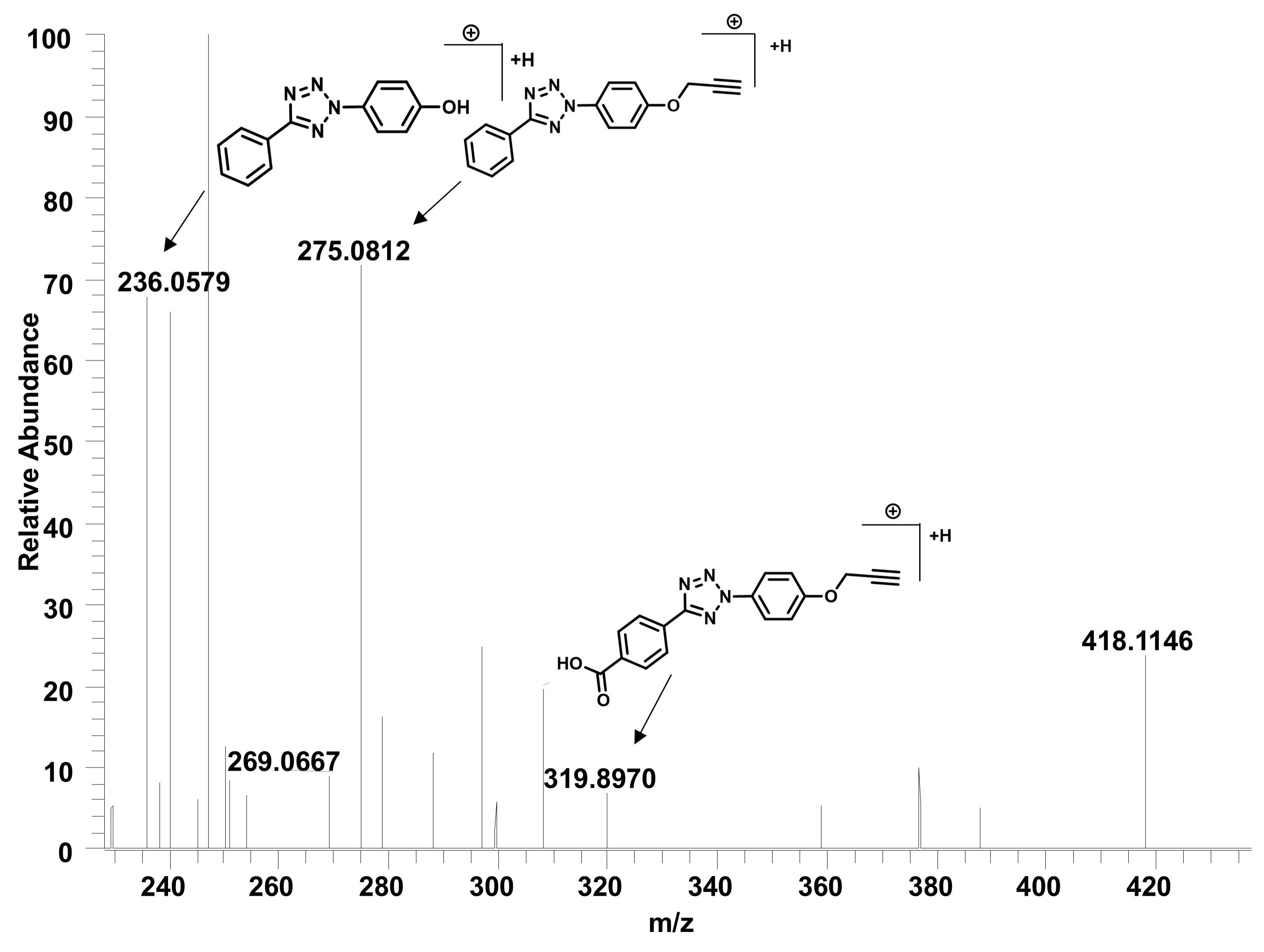


**SAP1**


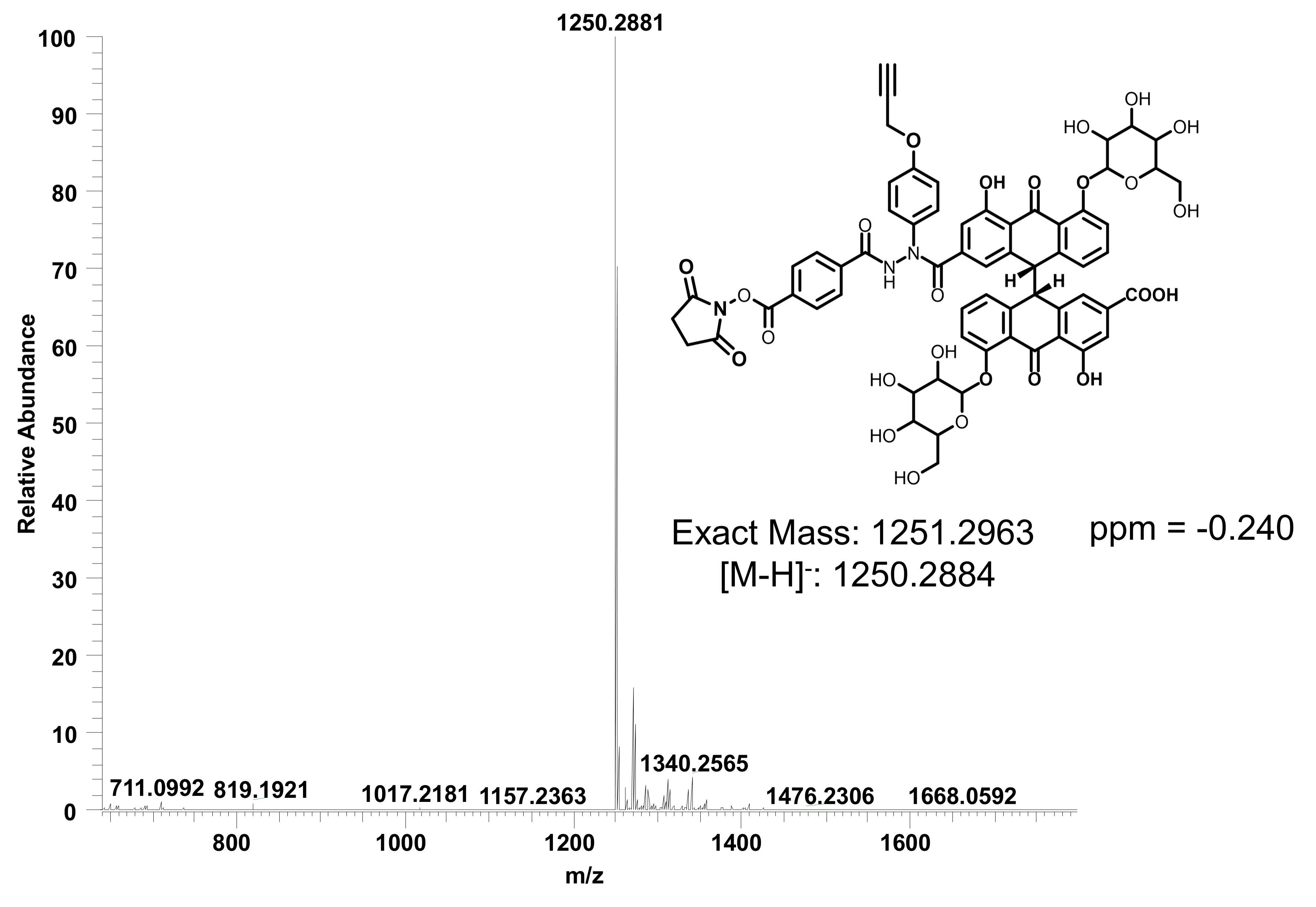

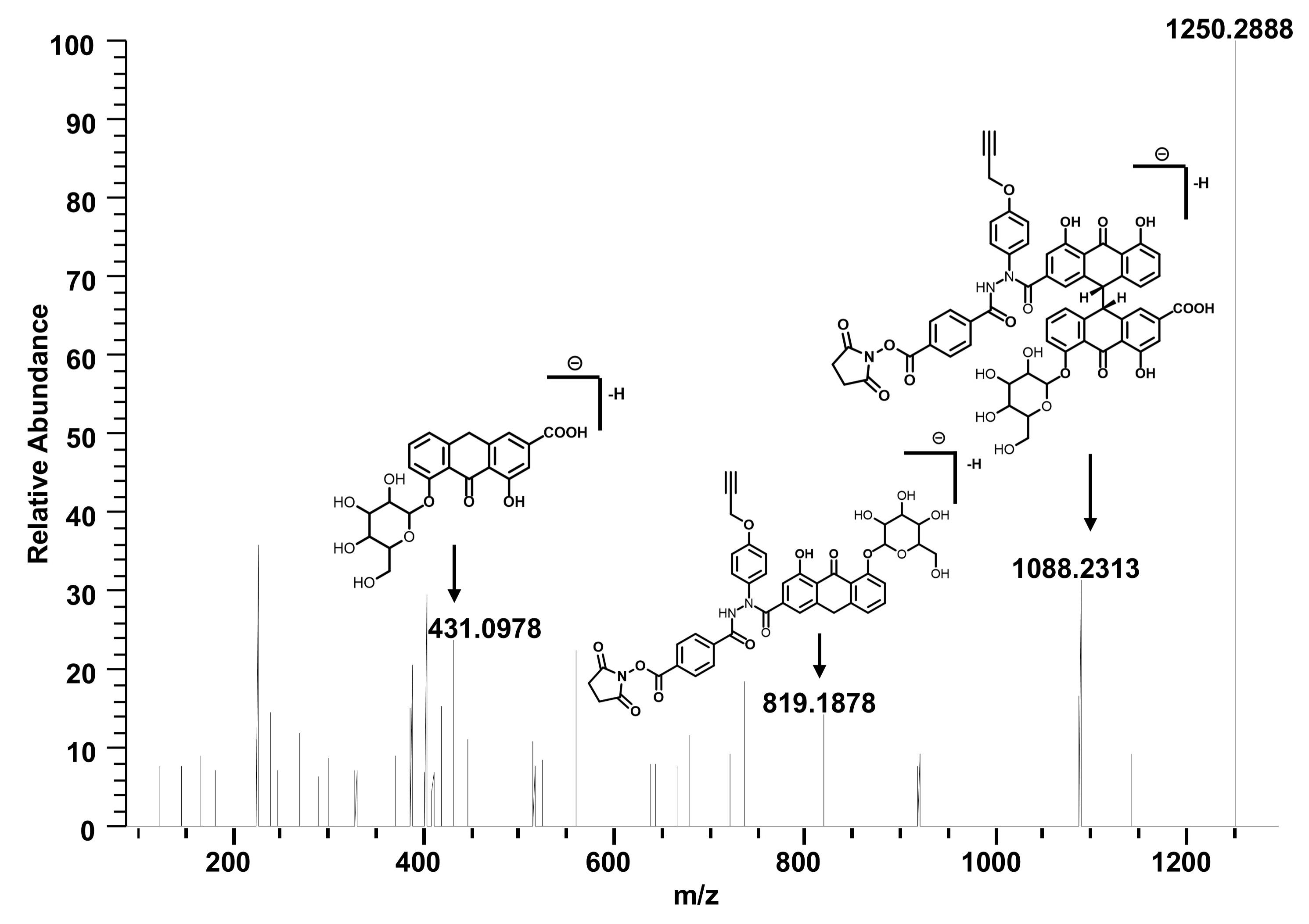


**NMR characterization data of SAP1**


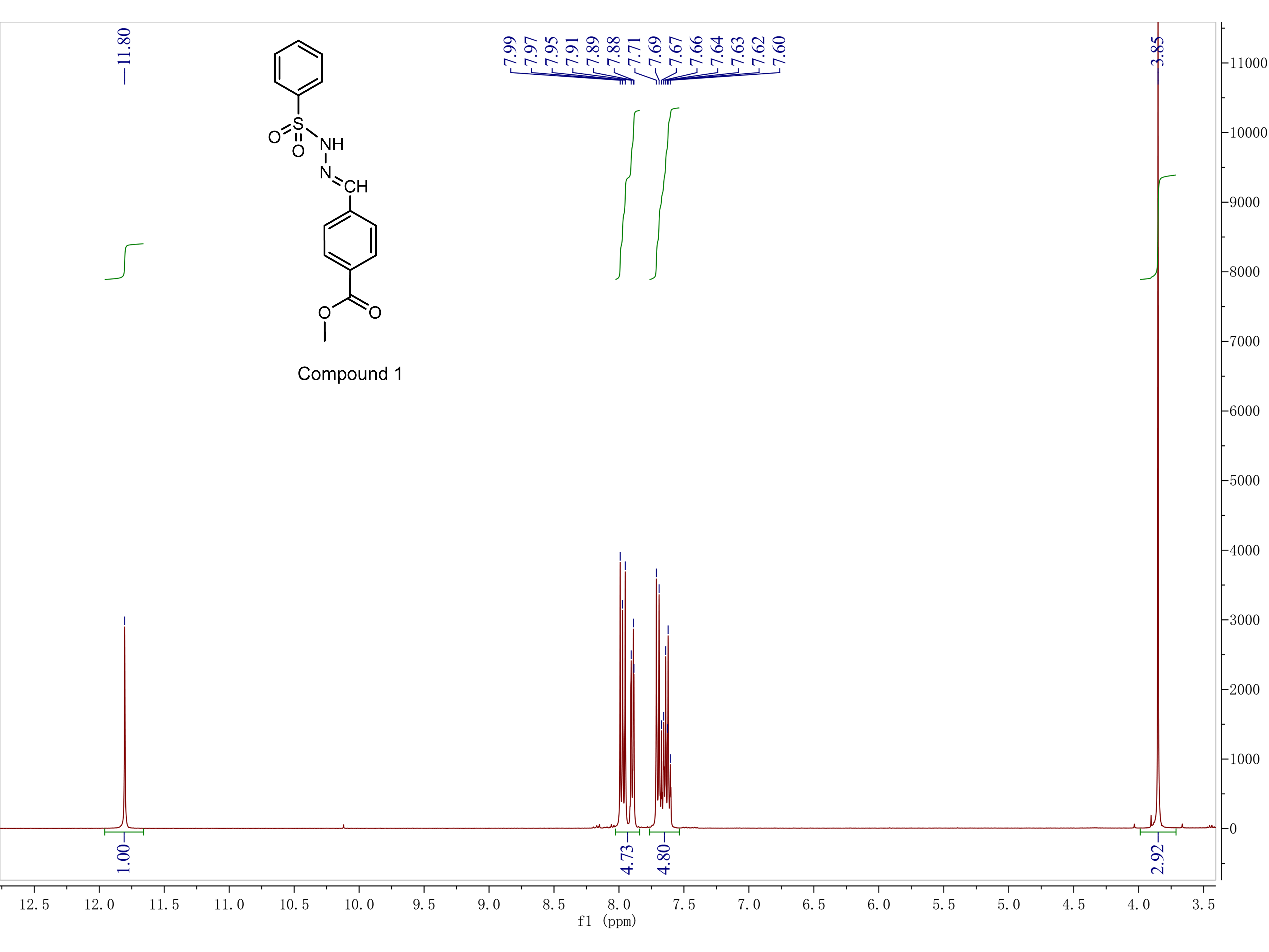

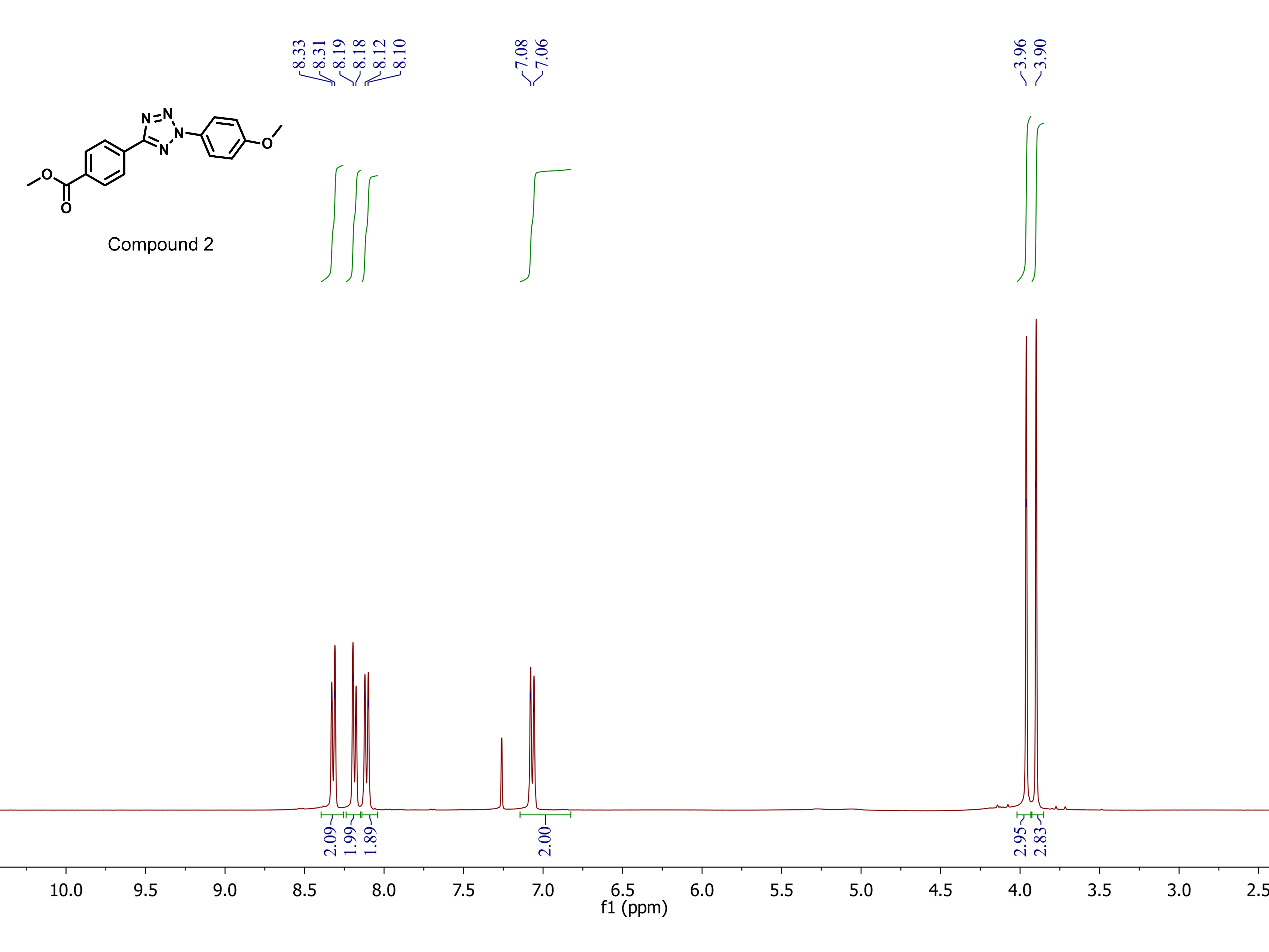

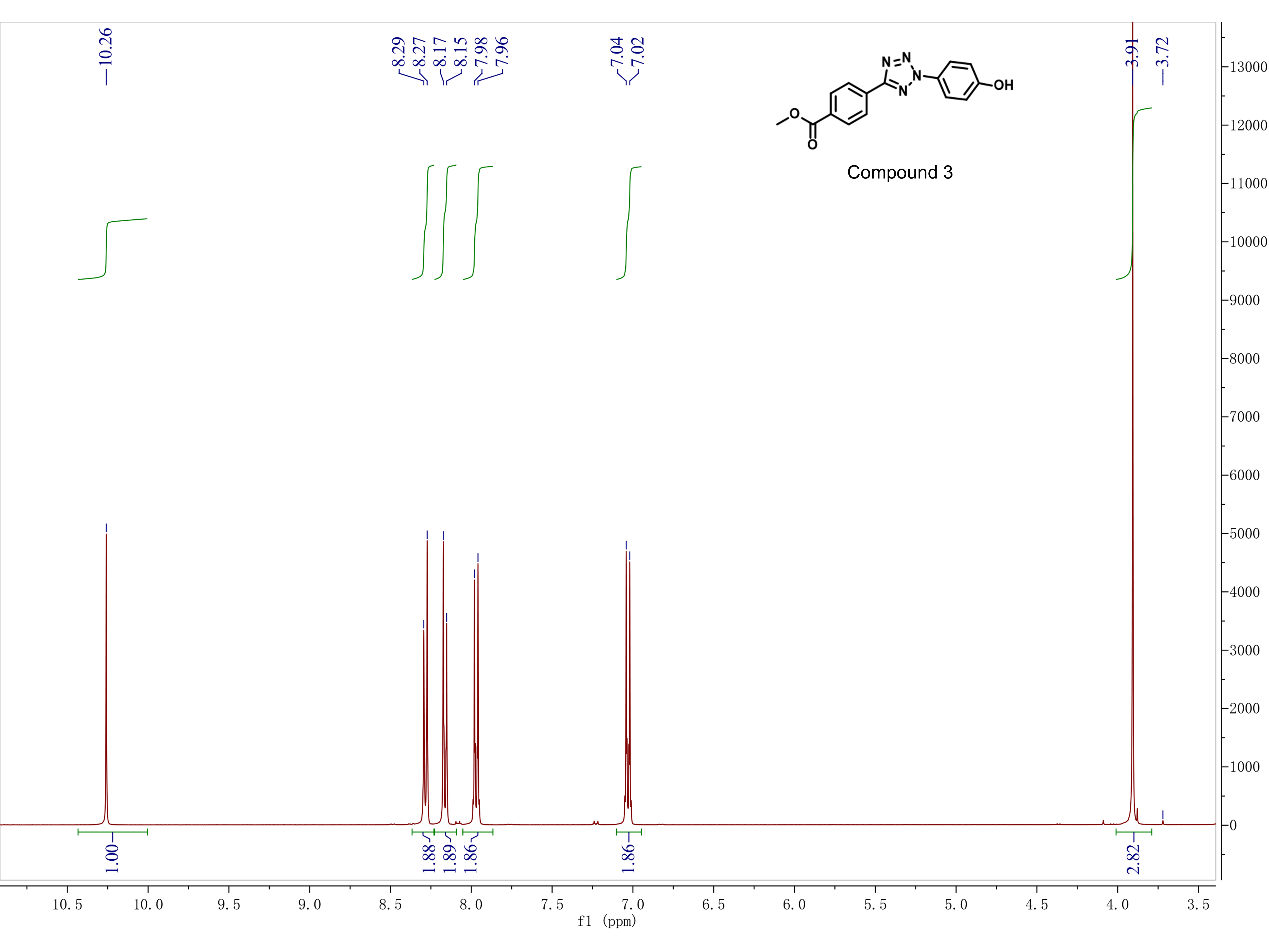

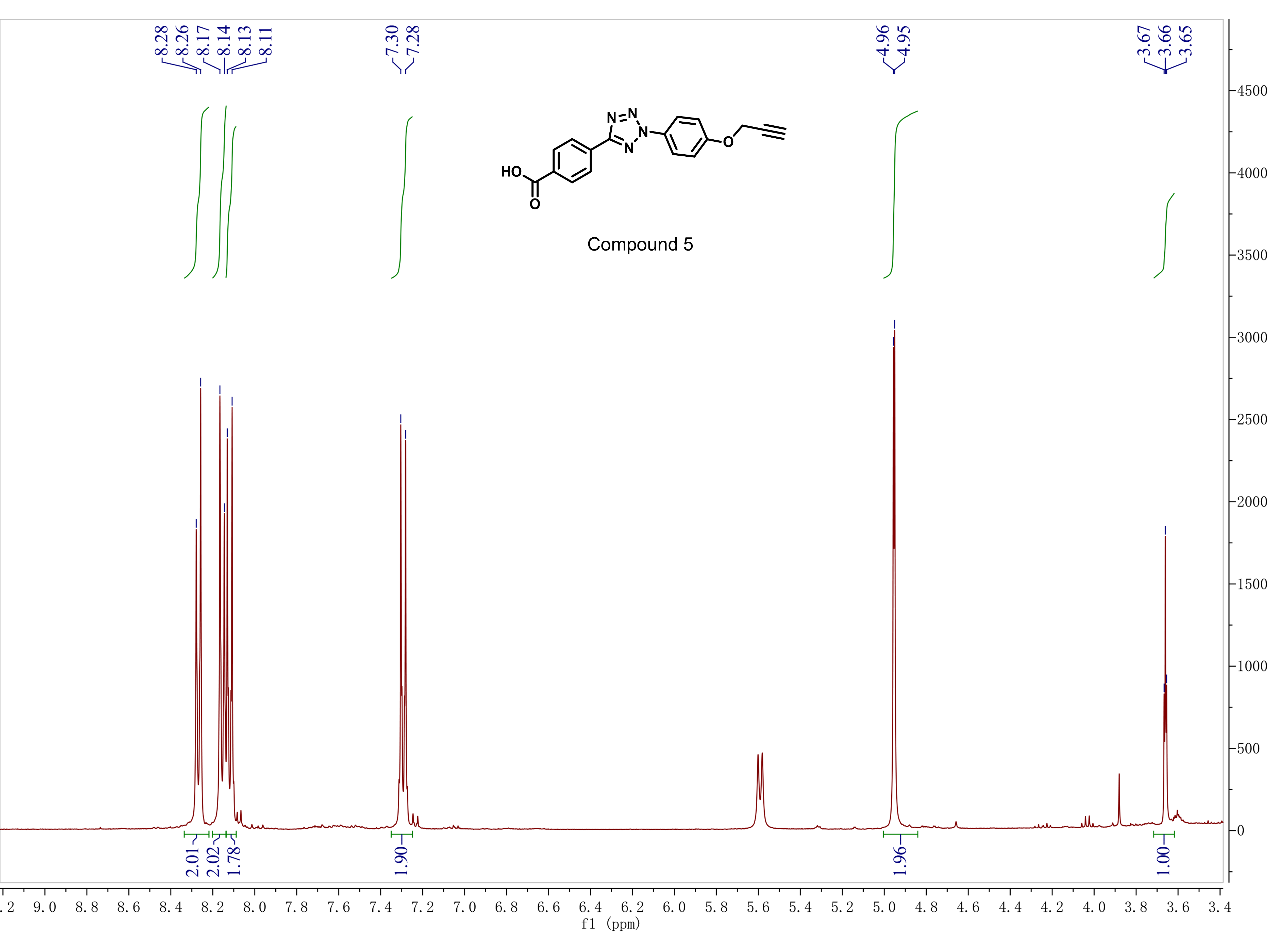

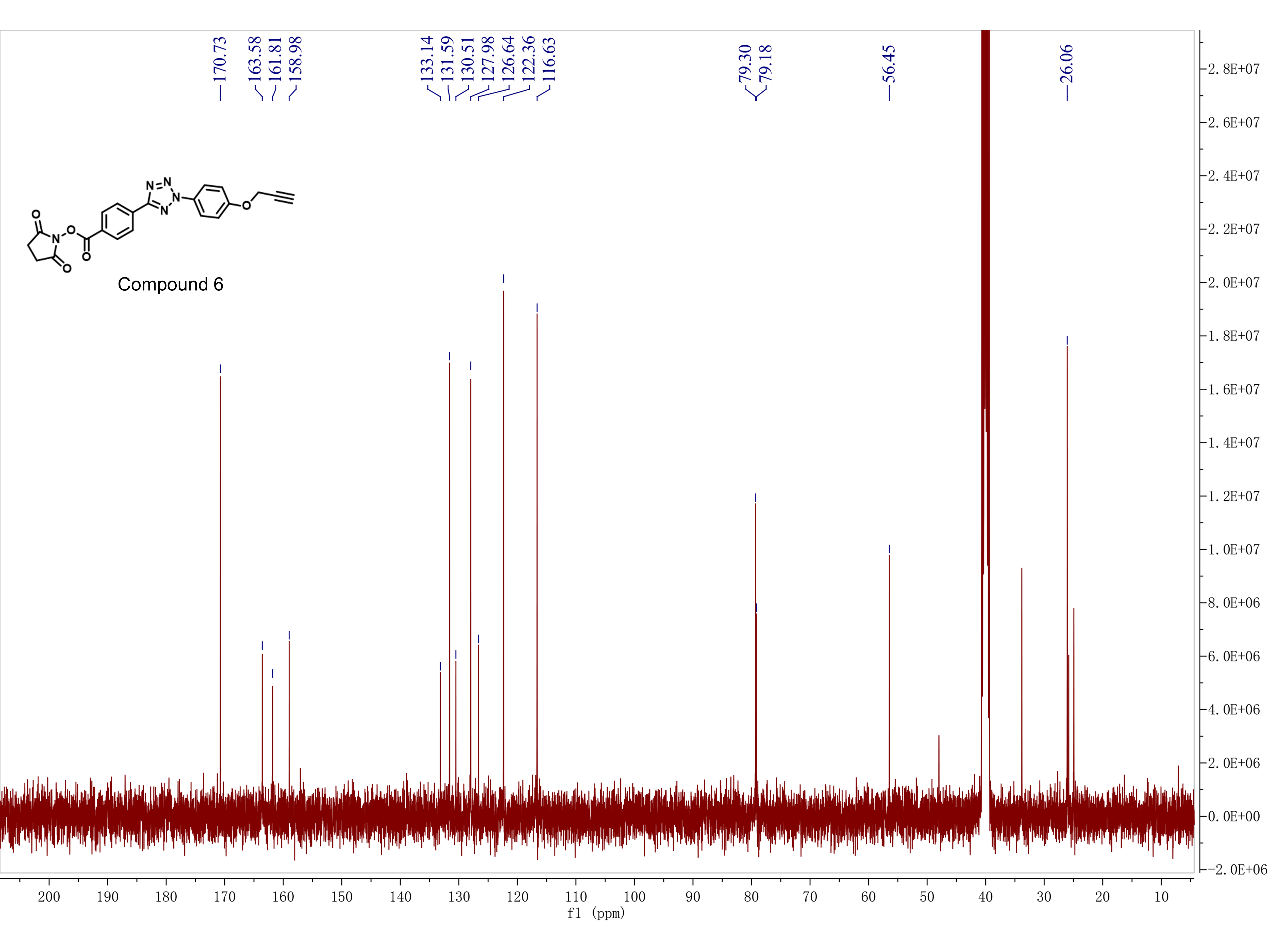

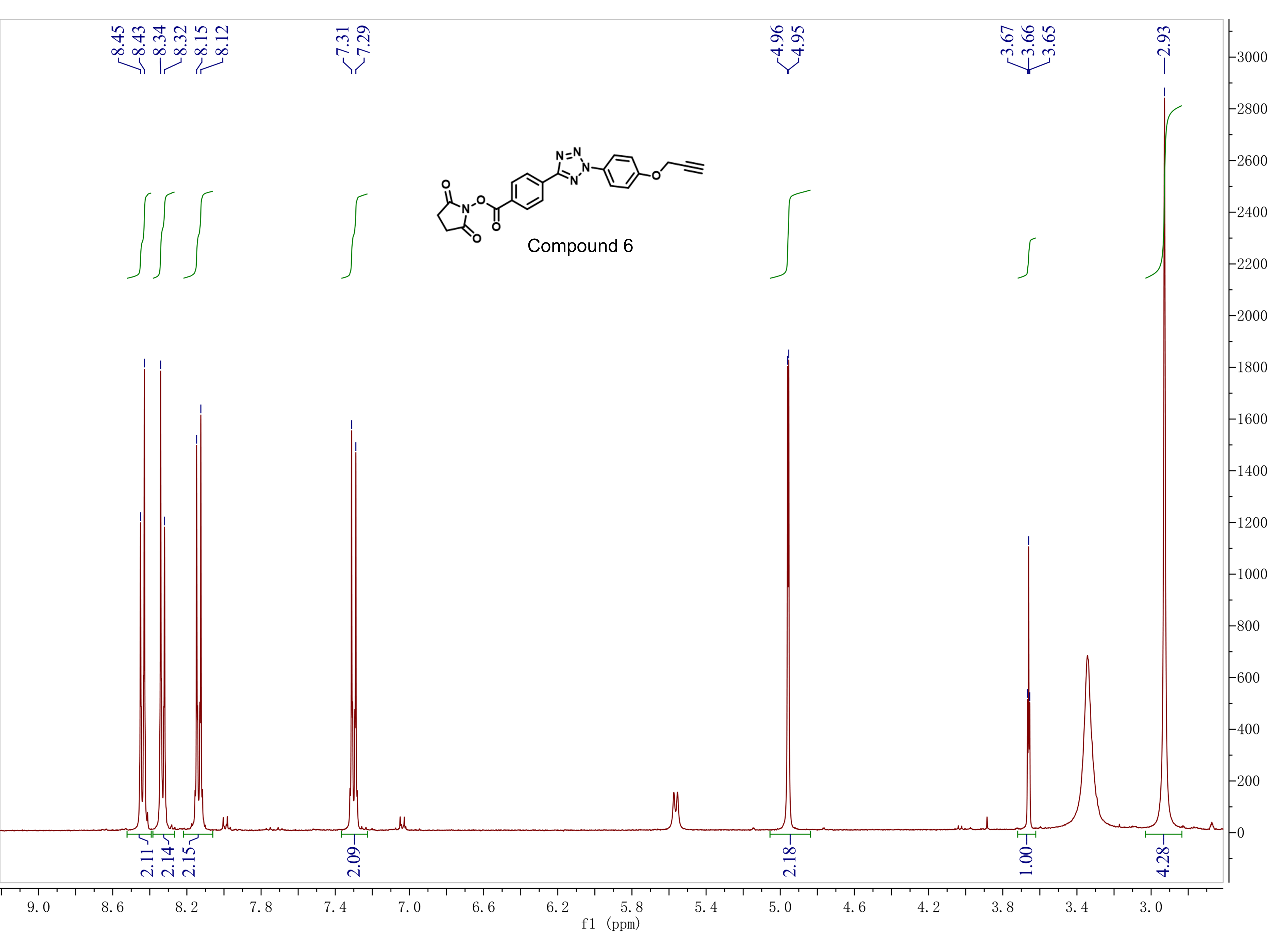


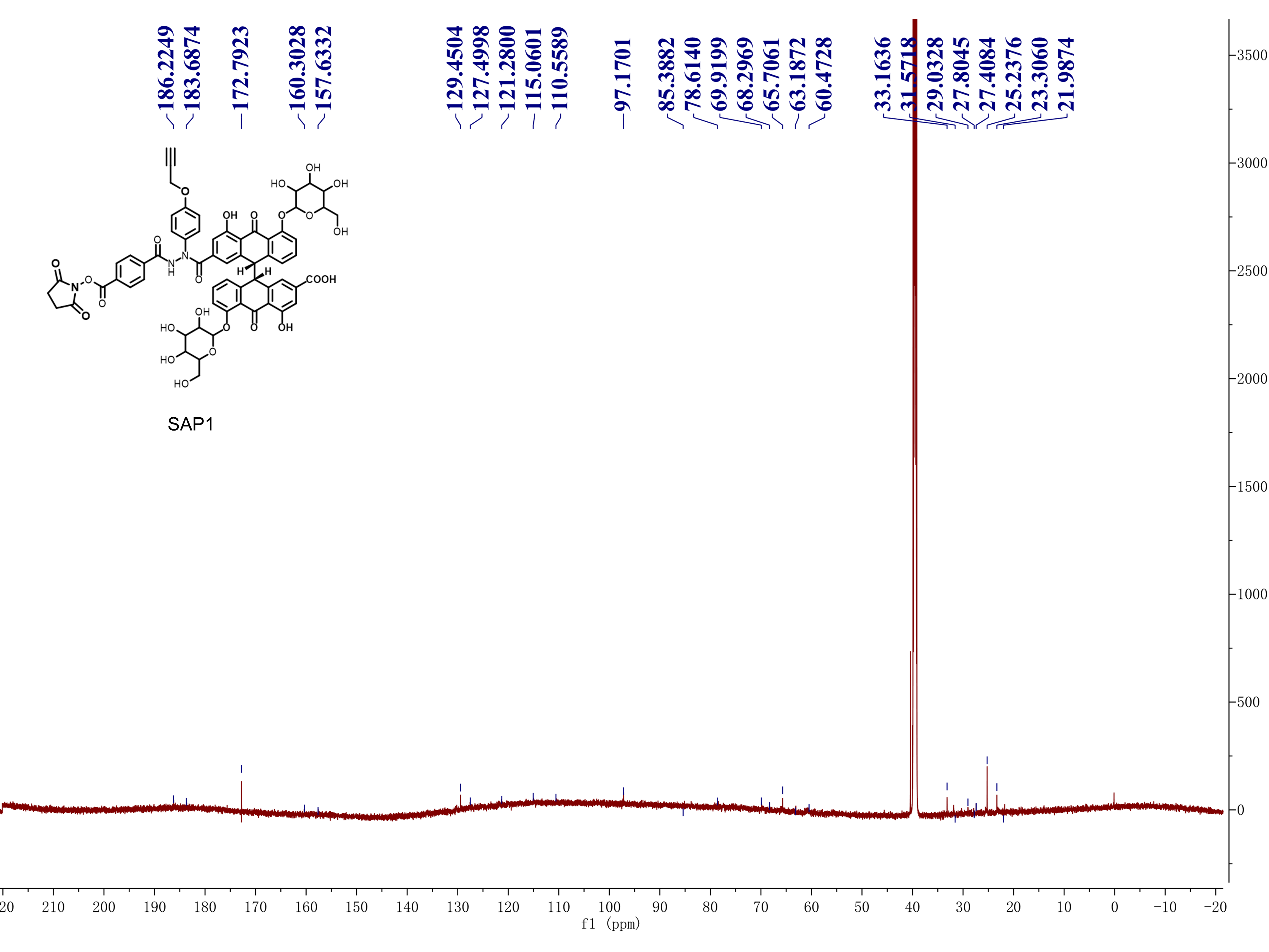


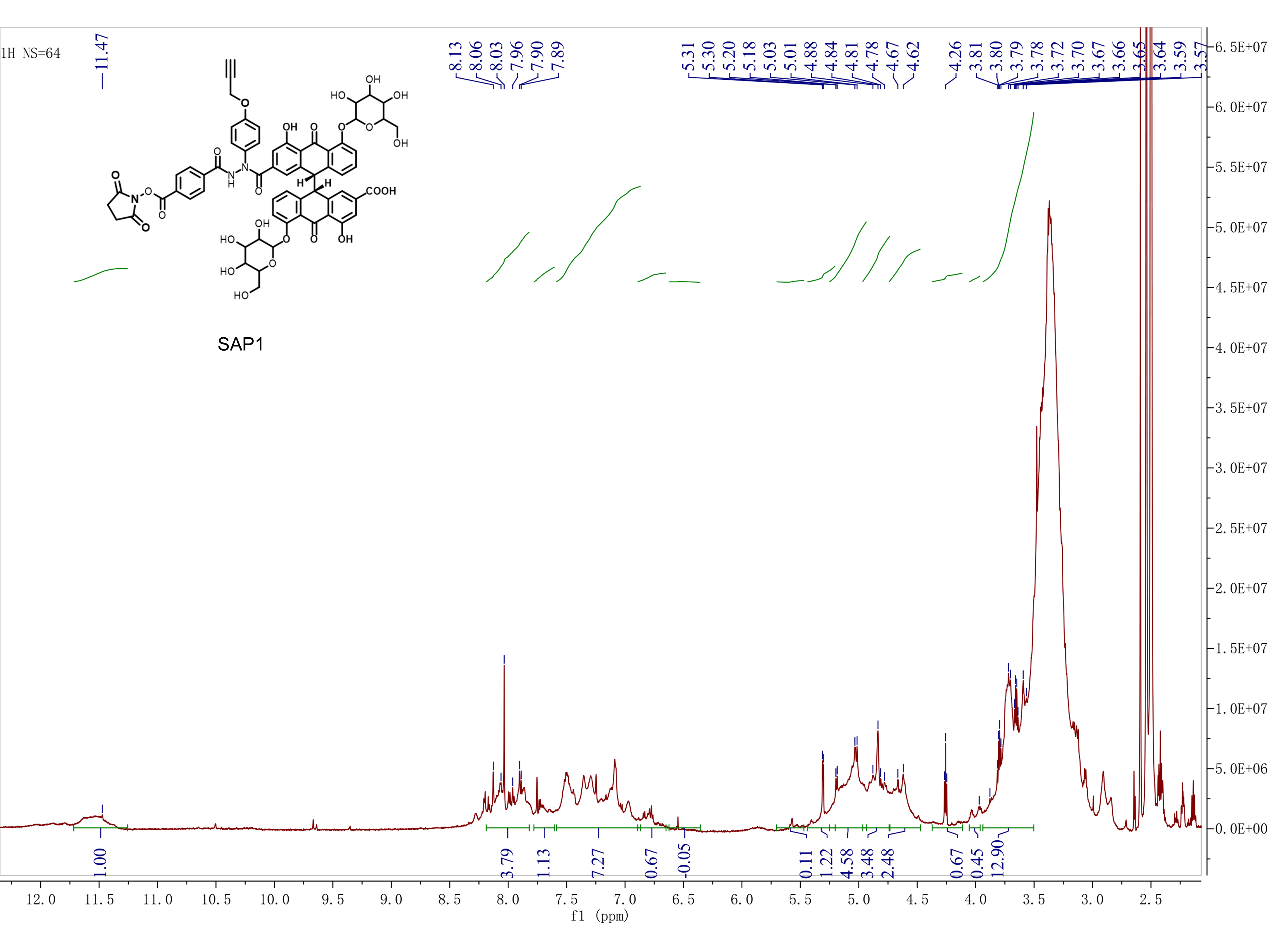


**MS spectra and MS/MS spectra of SAP2**


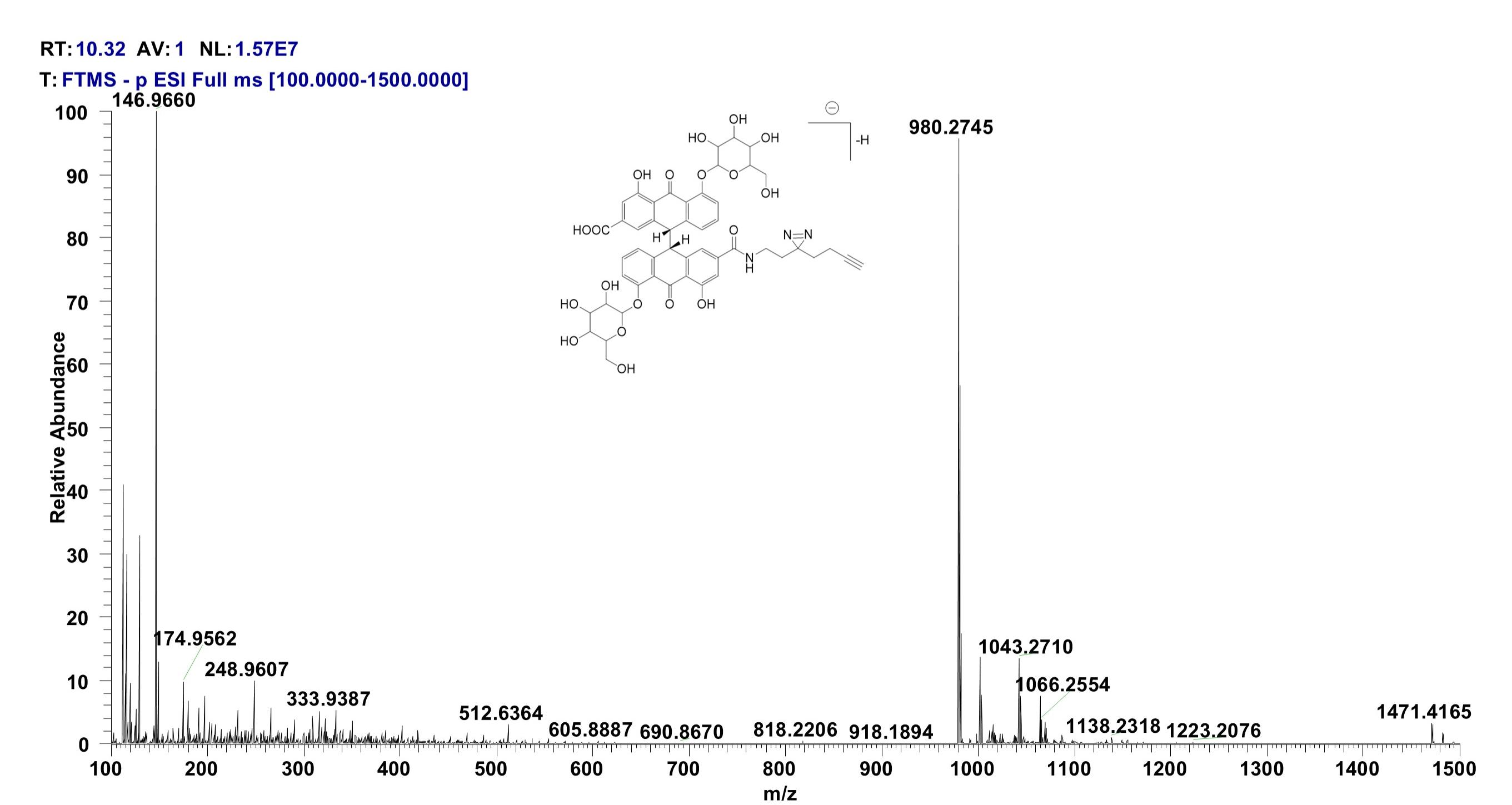

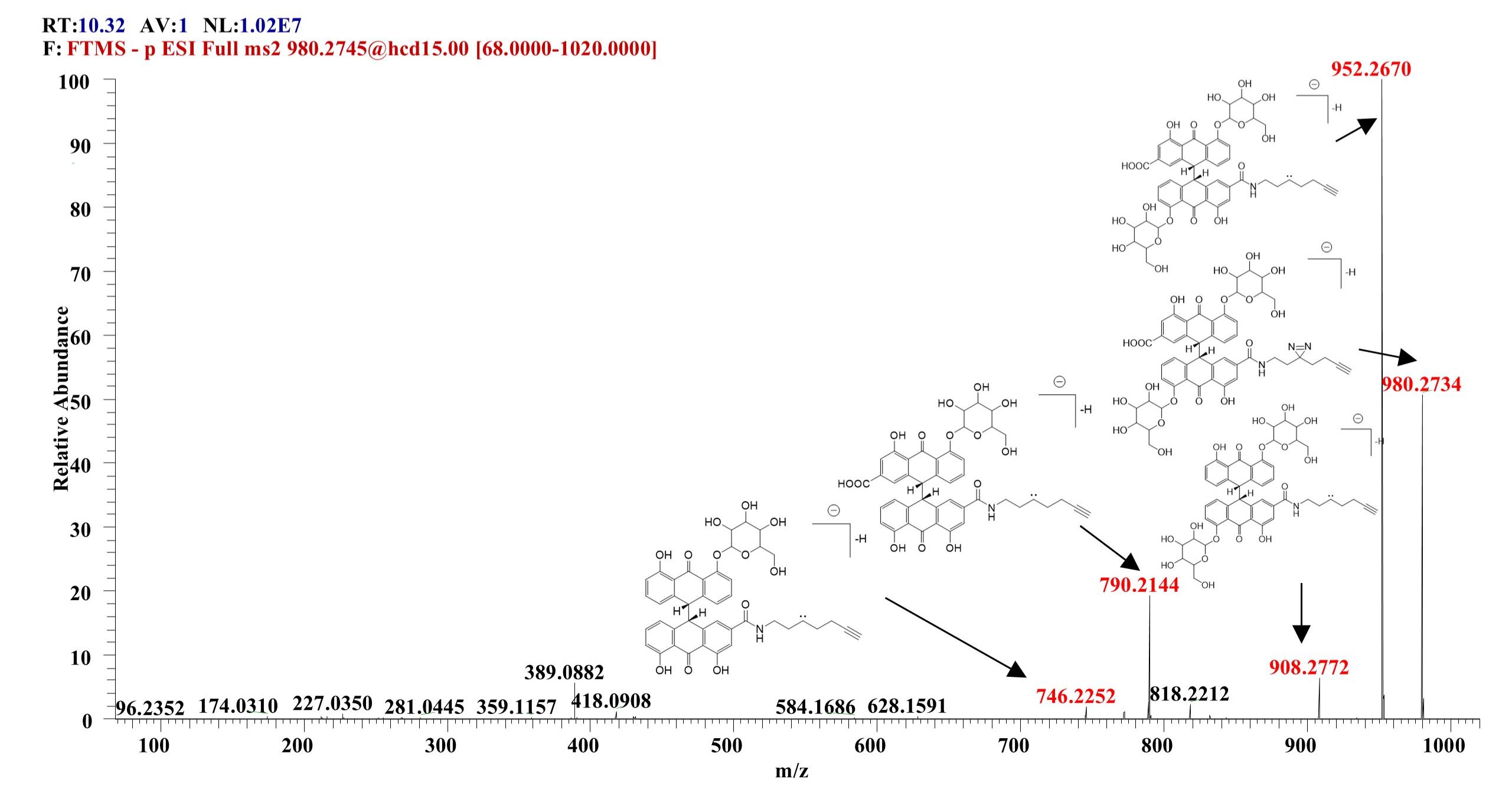


**^1^H NMR (600 MHz, DMSO-d_6_) and ^13^C NMR (151 MHz, DMSO) spectra of SAP2**

^1^H NMR (600 MHz, DMSO-d_6_) δ 11.55 (s, 2H, -OH), 8.49 (s, 1H, -OH), 7.59 (s, 2H, -OH), 7.39 (dd, *J* = 12.6, 8.4 Hz, 2H, Ar-H), 7.29 (d, *J* = 1.7 Hz, 2H, Ar-H), 7.18-6.31 (m, 6H, Ar-H), 5.15-5.03 (m, 3H, CH), 5.00 (d, *J* = 4.2 Hz, 1H, CH), 4.95 (d, *J* = 4.2 Hz, 1H, -OH), 4.88 (d, *J* = 7.6 Hz, 1H, -OH), 4.82 (d, *J* = 7.9 Hz, 1H, -OH), 4.74-4.58 (m, 3H, -OH), 3.83-3.69 (m, 2H, CH), 3.51-3.41 (m, 2H, CH), 3.27-3.20 (m, 2H, CH), 3.15 (dt, *J* = 8.9, 4.6 Hz, 2H, CH), 2.83 (t, *J* = 2.7 Hz, 1H, CH), 2.53-2.51 (m, 2H, CH_2_), 2.02 (td, *J* = 7.4, 2.7 Hz, 1H, CH_2_), 1.65 (dt, *J* = 19.4, 7.4 Hz, 3H, CH_2_), 1.23 (s, 1H, CH_2_)

^13^C NMR (151 MHz, DMSO) δ 187.25 (C=O), 186.98 (C=O), 167.34 (C=O), 159.69 (C=O), 158.25 (C=O), 158.03 (C=O), 118.98 (Ar-C), 118.31 (Ar-C), 116.93 (Ar-C), 113.54 (Ar-C), 103.21(-Glu), 83.67 (C), 78.02 (CH), 76.23 (CH), 75.94 (CH), 74.21 (CH), 72.30 (CH), 70.34 (C), 61.22 (CH_2_), 54.70 (CH), 35.01 (CH_2_), 32.28 (CH_2_), 31.73 (CH_2_), 27.78 (CH_2_), 13.19 (CH_2_)

**References**

[1] Sun L, Ding J, Xing W, Gai Y, Sheng J, Zeng D. Novel Strategy for Preparing Dual-Modality Optical/PET Imaging Probes via Photo-Click Chemistry. Bioconjugate Chem. 2016;27:1200-1204.

[2] Zhao S, Dai J, Hu M, Liu C, Meng R, Liu X, Wang C, Luo T. Photo-induced coupling reactions of tetrazoles with carboxylic acids in aqueous solution: application in protein labelling. Chem Commun. 2016;52:4702-4705.
